# Supplementary material for: Recommendations for hand hygiene in community settings: a scoping review of current international guidelines
Source: BMJ Open. 2023 Jun 21;13(6):e068887. doi: 10.1136/bmjopen-2022-068887 (PMC10314431; doi:10.1136/bmjopen-2022-068887)
Supplement: Supplementary data [file bmjopen-2022-068887supp001.pdf]

SUPPLEMENTAL MATERIALS

Table A1. The completed Preferred Reporting Items for Systematic reviews and Meta-Analyses extension for Scoping Reviews (PRISMA-ScR) checklist.[25]

SECTION	ITEM	PRISMA-ScR CHECKLIST ITEM	REPORTED ON PAGE #
**TITLE**			
Title	1	Identify the report as a scoping review.	2
**ABSTRACT**			
Structured summary	2	Provide a structured summary that includes (as applicable): background, objectives, eligibility criteria, sources of evidence, charting methods, results, and conclusions that relate to the review questions and objectives.	3
**INTRODUCTION**			
Rationale	3	Describe the rationale for the review in the context of what is already known. Explain why the review questions/objectives lend themselves to a scoping review approach.	5
Objectives	4	Provide an explicit statement of the questions and objectives being addressed with reference to their key elements (e.g., population or participants, concepts, and context) or other relevant key elements used to conceptualize the review questions and/or objectives.	5
**METHODS**			
Protocol and registration	5	Indicate whether a review protocol exists; state if and where it can be accessed (e.g., a Web address); and if available, provide registration information, including the registration number.	5
Eligibility criteria	6	Specify characteristics of the sources of evidence used as eligibility criteria (e.g., years considered, language, and publication status), and provide a rationale.	6
Information sources	7	Describe all information sources in the search (e.g., databases with dates of coverage and contact with authors to identify additional sources), as well as the date the most recent search was executed.	6
Search	8	Present the full electronic search strategy for at least 1 database, including any limits used, such that it could be repeated.	Supplemental Material – Table A3
Selection of sources of evidence	9	State the process for selecting sources of evidence (i.e., screening and eligibility) included in the scoping review.	6
Data charting process	10	Describe the methods of charting data from the included sources of evidence (e.g., calibrated forms or forms that have been tested by the team before their use, and whether data charting was done independently or in duplicate) and any processes for obtaining and confirming data from investigators.	7
Data items	11	List and define all variables for which data were sought and any assumptions and simplifications made.	7 and Supplemental Material – Table A5

SECTION	ITEM	PRISMA-ScR CHECKLIST ITEM	REPORTED ON PAGE #
Critical appraisal of individual sources of evidence	12	If done, provide a rationale for conducting a critical appraisal of included sources of evidence; describe the methods used and how this information was used in any data synthesis (if appropriate).	7
Synthesis of results	13	Describe the methods of handling and summarizing the data that were charted.	7
**RESULTS**			
Selection of sources of evidence	14	Give numbers of sources of evidence screened, assessed for eligibility, and included in the review, with reasons for exclusions at each stage, ideally using a flow diagram.	7 – 8
Characteristics of sources of evidence	15	For each source of evidence, present characteristics for which data were charted and provide the citations.	Supplemental material Table A7
Critical appraisal within sources of evidence	16	If done, present data on critical appraisal of included sources of evidence (see item 12).	15
Results of individual sources of evidence	17	For each included source of evidence, present the relevant data that were charted that relate to the review questions and objectives.	8 – 15
Synthesis of results	18	Summarize and/or present the charting results as they relate to the review questions and objectives.	8 – 15
**DISCUSSION**			
Summary of evidence	19	Summarize the main results (including an overview of concepts, themes, and types of evidence available), link to the review questions and objectives, and consider the relevance to key groups.	15 – 18
Limitations	20	Discuss the limitations of the scoping review process.	17 – 18
Conclusions	21	Provide a general interpretation of the results with respect to the review questions and objectives, as well as potential implications and/or next steps.	18
**FUNDING**			
Funding	22	Describe sources of funding for the included sources of evidence, as well as sources of funding for the scoping review. Describe the role of the funders of the scoping review.	20

Table A2. List of websites of international organisations known to work on hand hygiene searched.

Agency or resource hub	Website
Africa Centers for Disease Control and Prevention (CDC)	https://africacdc.org/
Global Handwashing Partnership	https://globalhandwashing.org/
Global WASH Cluster	www.washcluster.org
International Red Cross and Red Crescent (ICRC)	www.icrc.org
International Federation of the Red Cross (IFRC)	www.ifrc.org
International Labour Organization (ILO)	https://www.ilo.org
International Centre for Diarrhoeal Disease Research Bangladesh (ICDDR'B)	www.icddrb.org
Oxfam	www.oxfam.org.uk
Sanitation Learning Hub	https://sanitationlearninghub.org/
Save the Children	www.nrc.no
SNV Netherlands Development Organisation	https://snv.org/
Sustainable Sanitation Alliance	https://www.susana.org/en/
United Nations Human Settlement Programme (UN Habitat)	https://unhabitat.org/
United Nations High Commissioner for Refugees (UNHCR)	www.unhcr.org
United Nations Children's Fund (UNICEF)	www.unicef.org
US Centers for Disease Control and Prevention (CDC)	www.cdc.gov
WASH'Em	https://www.washem.info/
WaterAid	https://www.wateraid.org/us/
World Bank	https://www.worldbank.org/en/home
Water, Education and Development Centre (WEDC)	www.wedc.lboro.ac.uk
Water and Sanitation for the Urban Poor (WSUP)	https://www.wsup.com/
Deutsche Gesellschaft für Internationale Zusammenarbeit (GIZ)	https://www.giz.de
United States Agency for International Development (USAID)	https://www.usaid.gov/

Table A3. Search terms.

Concept	Search terms
Hand hygiene	Handwash* OR hygiene OR hand wash* OR with soap OR hand hygiene* OR clean hands OR WASH
Community settings	community OR household OR domestic OR public space OR institution* OR work* OR occupation* OR school OR prison OR market* OR relig* OR education* OR detention*
Guideline	guideline OR guide OR handbook OR manual OR toolkit

Table A4. Hand Hygiene for All Initiative core partners**.**

United Nations Children's Fund
International Labour Organization
World Bank
United Nations High Commission for Refugees
COVID-19 Hygiene Hub
Sanitation and Water for All
Global Handwashing Partnership
International Federation of Red Cross
WaterAid

Table A5. Data extraction template.

#	Hand hygiene parameter	Guideline title 1 (author, year)		Guideline title 2 (author, year)		Guideline title 3 (author, year)	
		Recommendation	Evidence level[1]	Recommendation	Evidence level	Recommendation	Evidence level
**General**							
1	Community setting(s)						
2	Other guideline specification (e.g., COVID-19)						
**Effective hand hygiene**							
3	Hand hygiene definition						
4	Effective/safe hand hygiene definition						
5	Terminology for defining when to practice hand hygiene						
6	Key moments						
7	Basic handwashing knowledge						
8	Basic hand hygiene knowledge						
**Minimum requirements for effective hand hygiene**							
**Handwashing with soap**							
10	Soap						
11	Soap quantity						
12	Handwashing services						
13	Handwashing with soap duration						

14	Water quality						
15	Water quantity						
16	Alternative water sources for handwashing						
17	Wastewater management						
18	Location of handwashing stations						
19	Spacing and number of users per handwashing station						
**Hand drying**							
20	Hand drying materials						
21	Disposal of hand drying materials						
22	Conditional recommendations						
**Handwashing with soap alternatives**							
23	Alcohol-based hand rub and alcohol content						
24	Hand hygiene duration						
25	Other materials for hand hygiene						
26	Conditional recommendations						
27	Alternatives not recommended						
28	Location of hand hygiene stations						
**Hand hygiene station design considerations**							
29	Design principles						
30	Technical considerations						
31	Accessibility						
32	Local procurement and production						
33	Supply chain						
34	Cost and affordability						
35	Durability						
36	COVID-19 adaptations						
37	Other adaptations						

38	Operation and maintenance – responsibility						
39	Operation and maintenance – actions						
**Behaviour change**							
40	Behaviour change techniques						
41	Content of behaviour change messaging						
42	Behaviour change delivery channel						
43	Person or group tasked with delivering behaviour change intervention						
44	Frequency of behaviour change interventions						
45	Formative research						
46	Behaviour change models or frameworks						
47	Behaviour change approaches						
48	Hand hygiene determinants						
**Government measures – at-scale interventions**							
49	Strengthening markets for hand hygiene products and services						
50	Hygiene promotion practices						
51	At-scale behaviour change interventions						
**Government measures – SWA framework**							
52	Sector policy and strategy						
53	Institutional arrangements						
54	Sector financing						
55	Planning, monitoring, and review						
56	Capacity development						
57	Equity						

[1] One of the five levels of evidence used for the review: 1) systematic review or meta-analysis, 2) randomised controlled trial or controlled trial without randomisation (e.g., quasi-experimental), 3) guideline (either developed from systematic reviews or not developed from systematic reviews), 4) observational studies (e.g., cohort, cross-sectional, case-control studies), and 5) expert opinion or anecdotal information (e.g., programme documentation).

Table A6. Excluded documents with reasons.

Author	Guideline title	Year	Reason for exclusion
ICRC	Water, sanitation, and hygiene, and habitat in prisons	2005	Historical version of guideline
World Bank	The Handwashing Handbook: a guide for developing a hygiene promotion program to increase handwashing with soap	2005	Historical version of guideline
IRC	Strengthening Water, Sanitation and Hygiene in schools: a WASH guidance manual with a focus on South Asia	2010	Not a guideline
ICRC	Water, sanitation, and hygiene, and habitat in prisons: supplementary guidance	2012	No recommendation on hand hygiene
GIZ	Field guide: hardware for handwashing in schools	2013	Not a guideline
WEDC	Guidelines for handwashing with soap	2013	Not issued by an international agency
NRC	Water, sanitation and hygiene manual: WASH training for hygiene promotion staff	2015	Not a guideline
ILO	WASH@Work: self-training handbook	2016	Historical version of guideline
WSUP	School hygiene manual	2016	Not for international audience
Rotary	A guide to WASH in schools	2017	Not a guideline
Global Handwashing Partnership	Clean hands for all: a toolkit for hygiene advocacy	2018	Not a guideline
COOPI	WASH handbook for teachers and facilitators	2019	Not for international audience
WASH’Em	How to design handwashing facilities that change behaviour	2019	Does not target a community setting
Africa CDC	Hand washing facility options for resource limited settings	2020	Not a guideline
British Psychological Society	Behavioural science and disease prevention psychological guidance: encouraging hand hygiene in the community	2020	Not a guideline

Coultas et al.	Handwashing compendium for low-resource settings: a living document, 3rd edition	2020	Not a guideline
Global Handwashing Partnership	The Handwashing Handbook	2020	Not a guideline
Smart Centre Group	Smart hygiene solutions: affordable options for household level	2020	Not a guideline
UN Habitat	UN-Habitat guidance on COVID-19 and public spaces	2020	No recommendation on hand hygiene
UNHCR	COVID-19 refugees return to schooling guidelines	2020	No recommendation on hand hygiene
UNICEF et al.	Panduan Cuci Tangan Pakai Sabun	2020	Not in English or French
WaterAid	COVID-19 guidance: prioritising hygiene for workforce health and business resilience	2020	No recommendation on hand hygiene
WaterAid Nepal	Technical brief on contactless handwashing stations	2020	Not a guideline
Bolton, L.	WASH in schools for student return during the COVID-19 pandemic	2021	Not issued by an international agency
ILO	WASH@Work: self-training handbook (revised)	2021	Not a guideline
Government of Vietnam	A guide to integrated hand washing with soap communication	n/d	Not for international audience
WaterAid	Technical manual on community water supply, hygiene and sanitation facilities	n/d	Not a guideline
WaterAid Bangladesh	Handwashing stations: an easy-to-use technological and context-based handwashing stations manual	n/d	Not a guideline

Table A7. List of included guidelines.

Author	Guideline	Year	Community setting	COVID-19 response
UNICEF	A manual on hygiene promotion	1999	Public	No
WHO	WHO recommended strategies for the prevention and control of communicable diseases	2001	Domestic & public	No
WHO	Health villages: a guide for communities and community health workers	2002	Public	No
WHO	Water, sanitation, and hygiene standards for schools in low-cost settings	2009	Institutional	No
ICRC	Water, sanitation, hygiene, and habitat in prisons	2013	Institutional	No
ICRC	Health care in detention	2015	Institutional	No
WHO	Public health for mass gatherings: key considerations	2015	Public	No
SNV	Behaviour change communication guidelines	2016	Public	No
UNICEF, GIZ	Scaling up group handwashing in schools: compendium of group washing facilities across the globe	2016	Schools	No
Malteser	WASH guidelines for field practitioners	2017	Multiple settings	No
Oxfam	Handwashing technical briefing note	2017	Public	No
UNHCR	UNHCR hygiene promotion guidelines	2017	Public	No
WHO	Guidelines on sanitation and health	2018	Domestic & public	No
WHO	Recommendations to Member States to improve hand hygiene practices to help prevent COVID-19	2020	Public	Yes
UNICEF	Handwashing stations and supplies for the COVID-19 response	2020	Domestic & public	Yes
ILO	Hand hygiene at the workplace: an essential occupational safety and health prevention and control measure against COVID-19	2020	Workplace	Yes
GOAL	WASH and IPC measures in households and public spaces	2020	Domestic & public	No
WHO	Considerations for community hand hygiene practices in low-resource situations	2020	Public	No
WHO	Overview of public health and social measures in the context of COVID-19	2020	Public	Yes
GWC	COVID-19 response guidance note - 15 April 2020	2020	Domestic & public	Yes
GWC	Health and hygiene promotion guidance document for COVID-19	2020	Public	Yes
ILO	Safe return to work: guide for employers on COVID-19 prevention	2020	Institutional	Yes
SNV	Practical options for hand-washing stations: a guide for promoters and producers	2020	Multiple settings	No
UNHCR	Technical WASH guidance for COVID-19 preparedness and response	2020	Public	Yes
UNICEF	COVID-19 emergency preparedness and response: WASH and infection prevention and control measures in schools	2020	Schools	Yes
UNICEF	COVID-10 handwashing with soap (HWWS) facilities: compendium of indicative layouts, designs, and cost estimates	2020	Public	Yes

UNICEF et al.	Handwashing with soap in schools: a technical options manual	2020	Schools	No
UNICEF, WHO, IFRC	Key messages and actions for COVID-19 prevention and control in schools	2020	Schools	Yes
UNICEF	Understanding Hygiene promotion in the context of Risk Communication & Community Engagement (RCCE) and Infection Control and Prevention (IPC) for the COVID-19 outbreak	2020	Domestic & public	Yes
UNICEF	WASH and Infection Prevention and Control (IPC) measures in households and public spaces	2020	Domestic & public	Yes
UNICEF, WHO, IFRC	Interim guidance for COVID-19 prevention and control in schools	2020	Institutional	Yes
WASH4WORK	Hand hygiene protocol for the workplace	2020	Institutional	Yes
Water Mission	Handwashing station program: requirements and recommendations	2020	Public	Yes
WaterAid	Technical guide for handwashing facilities in public spaces and buildings	2020	Public	Yes
WHO	Actions for consideration in the case and protection of vulnerable populations from COVID-19	2020	Vulnerable populations	Yes
WHO	Considerations for public health and social measures in the workplace in the context of COVID-19: Annex to Considerations in adjusting public health and social measures in the context of COVID-19	2020	Workplace	Yes
WHO	Considerations for school-related public health measures in the context of COVID-19: Annex to Considerations in adjusting public health and social measures in the context of COVID-19	2020	Institutional	Yes
WHO	Guidance on COVID-19 for the care of older people and people living in long-term care facilities, other non- acute care facilities and home care	2020	Institutional	Yes
WHO	Interim recommendations on obligatory hand hygiene against transmission of COVID-19	2020	Public spaces	Yes
WHO	Practical considerations and recommendations for religious leaders and faith-based communities in the context of COVID-19	2020	Public spaces	Yes
WHO	Water, sanitation, hygiene, and waste management for SARS-CoV-2, the virus that causes COVID-19: interim guidance	2020	Domestic & public	Yes
CDC	When and how to wash your hands	2021	Public	No
CDC	Hand hygiene at school	2021	Institutional	No
CDC	Hand hygiene at work	2021	Institutional	No
CDC	Interim guidance on management of coronavirus disease 2019 (COVID-19) in correctional and detention facilities	2021	Institutional	Yes
CDC	Interim guidance on people experiencing unsheltered homelessness	2021	Vulnerable populations	Yes
WHO	Key planning recommendations for mass gatherings in the context of COVID-19: interim guidance	2021	Public	Yes

WHO	Preparedness, prevention, and control of COVID-19 in prisons and other places of detention: interim guidance	2021	Institutional	Yes
WHO, ILO	Preventing and mitigating COVID-19 at work	2021	Institutional	Yes
WHO	Safe Eid al Adha practices in the context of COVID-19: interim guidance	2021	Public	Yes
WHO	Safe Ramadan practices in the context of COVID-19: interim guidance	2021	Public	Yes

Table A8. Number of recommendations per parameter across all community settings.

Parameters	Number of recommendations	Number of guidelines with recommendations
**Effective hand hygiene**		
Guidelines that define hand hygiene	3	3
Guidelines that define safe hand hygiene	2	2
Terminology for defining when to practice safe hand hygiene	38	38
Critical control points	145	38
Basic handwashing knowledge	8	8
Basic hand hygiene knowledge	0	0
**Handwashing with soap & hand drying**		
Handwashing infrastructure	33	24
Handwashing materials	68	46
Amount of soap	7	6
Handwashing services	37	30
Handwashing with soap duration	17	17
Water quality	19	16
Water quantity	9	10
Alternative water sources for handwashing	15	16
Wastewater management	23	13
Location of handwashing stations	59	30
Spacing and number of users per handwashing station	9	8
Hand drying materials	36	19
Disposal of hand drying materials	9	9
Conditional recommendations	7	7
**Handwashing with soap alternatives**		
Alcohol-based hand rub	32	32
Hand rubbing duration	3	3
Other materials for hand hygiene	39	17
Conditional recommendations	29	22
Alternatives not recommended	11	7
Location of hand hygiene stations	21	12
**Hand hygiene station design considerations**		
Design principles	10	6
Technical considerations	5	4
Accessibility	19	14
Local procurement and production	11	9
Supply chain	3	3
Cost and affordability	7	7
Durability	8	6
COVID-19 adaptations	26	16
Other adaptations	17	10
Operation and maintenance – responsibility	8	6
Operation and maintenance – actions	9	8
**Changing behaviour**		
Behaviour change techniques	29	14
Content of behaviour change messaging	27	25
Behaviour change delivery channels	42	25
Person or group tasked with delivery behaviour change intervention	13	10
Frequency of behaviour change interventions	2	2
Formative research	5	4

Behaviour change models or frameworks	15	3
Behaviour change approaches	9	2
Hand hygiene determinants	11	3
**Government interventions**		
Strengthening markets for hand hygiene products and services	2	2
Hygiene promotion practices	0	0
At-scale behaviour change interventions	0	0
Sector policy and strategy	0	0
Institutional arrangements	5	5
Sector financing	0	0
Planning, monitoring, and review	2	2
Capacity development	1	1
Equity	1	1
