# Supplementary material for: Recommendations for hand hygiene in community settings: a scoping review of current international guidelines
Source: BMJ Open. 2023 Jun 21;13(6):e068887. doi: 10.1136/bmjopen-2022-068887 (PMC10314431; doi:10.1136/bmjopen-2022-068887)
Supplement: Author's manuscript [file bmjopen-2022-068887.draft_revisions.pdf]

BMJ Open

BMJ Open is committed to open peer review. As part of this commitment we make the peer review history of every article we publish publicly available.

When an article is published we post the peer reviewers' comments and the authors' responses online. We also post the versions of the paper that were used during peer review. These are the versions that the peer review comments apply to.

The versions of the paper that follow are the versions that were submitted during the peer review process. They are not the versions of record or the final published versions. They should not be cited or distributed as the published version of this manuscript.

BMJ Open is an open access journal and the full, final, typeset and author-corrected version of record of the manuscript is available on our site with no access controls, subscription charges or pay-per-view fees (http://bmjopen.bmj.com).

If you have any questions on BMJ Open's open peer review process please email info.bmjopen@bmj.com

BMJ Open

Improving hand hygiene in community settings: a scoping review of current international guidelines

Journal:	*BMJ Open*
Manuscript ID	bmjopen-2022-068887
Article Type:	Original research
Date Submitted by the Author:	04-Oct-2022
Complete List of Authors:	MacLeod, Clara; London School of Hygiene and Tropical Medicine Faculty of Infectious and Tropical Diseases, Department of Disease Control<br>Braun, Laura; London School of Hygiene and Tropical Medicine Faculty of Infectious and Tropical Diseases, Department of Disease Control<br>Caruso, Bethany; Emory University, Hubert Department of Global Health, Rollins School of Public Health<br>Chase, Claire; World Bank Group<br>Chidziwisano, Kondwani; Malawi University of Business and Applied Sciences, Department of Environmental Health and WASHTED<br>Chipungu, Jenala; Center for Infectious Disease Research in Zambia<br>Dreibelbis , Robert; London School of Hygiene and Tropical Medicine Faculty of Infectious and Tropical Diseases, Department of Disease Control<br>Ejemot-Nwadiaro, Regina; University of Calabar, Department of Public Health, College of Medical Sciences<br>Gordon, Bruce ; World Health Organization, Water, Sanitation, Hygiene and Health Unit<br>Esteves Mills, Joanna; World Health Organization, Water, Sanitation, Hygiene and Health Unit<br>Cumming, Oliver; London School of Hygiene and Tropical Medicine Faculty of Infectious and Tropical Diseases, Department of Disease Control
Keywords:	Public health < INFECTIOUS DISEASES, PUBLIC HEALTH, COVID-19

SCHOLARONE™
Manuscripts

I, the Submitting Author has the right to grant and does grant on behalf of all authors of the Work (as defined in the below author licence), an exclusive licence and/or a non-exclusive licence for contributions from authors who are: i) UK Crown employees; ii) where BMJ has agreed a CC-BY licence shall apply, and/or iii) in accordance with the terms applicable for US Federal Government officers or employees acting as part of their official duties; on a worldwide, perpetual, irrevocable, royalty-free basis to BMJ Publishing Group Ltd ("BMJ") its licensees and where the relevant Journal is co-owned by BMJ to the co-owners of the Journal, to publish the Work in this journal and any other BMJ products and to exploit all rights, as set out in our licence.

The Submitting Author accepts and understands that any supply made under these terms is made by BMJ to the Submitting Author unless you are acting as an employee on behalf of your employer or a postgraduate student of an affiliated institution which is paying any applicable article publishing charge ("APC") for Open Access articles. Where the Submitting Author wishes to make the Work available on an Open Access basis (and intends to pay the relevant APC), the terms of reuse of such Open Access shall be governed by a Creative Commons licence – details of these licences and which Creative Commons licence will apply to this Work are set out in our licence referred to above.

Other than as permitted in any relevant BMJ Author's Self Archiving Policies, I confirm this Work has not been accepted for publication elsewhere, is not being considered for publication elsewhere and does not duplicate material already published. I confirm all authors consent to publication of this Work and authorise the granting of this licence.

For peer review only

Title

Improving hand hygiene in community settings: a scoping review of current international guidelines

Authors

Clara MacLeod[1,*], Laura Braun[1], Bethany A. Caruso[2], Claire Chase[3], Kondwani Chidziwisano[4], Jenala Chipungu[5], Robert Dreibelbis[1], Regina Ejemot-Nwadiaro[6], Bruce Gordon[7], Joanna Esteves Mills[7], Oliver Cumming[1]

Affiliations

[1] Department of Disease Control, London School of Hygiene and Tropical Medicine, Keppel St, London, WC1E 7HT, UK
[2] Hubert Department of Global Health, Rollins School of Public Health, Emory University, Atlanta, Georgia,
[3] The World Bank, Washington, DC 20433, United States of America
[4] Department of Environmental Health and WASHTED, Malawi University of Business and Applied Sciences, Blantyre, Malawi
[5] Centre for Infectious Disease Research in Zambia, Lusaka, Zambia
[6] Department of Public Health, College of Medical Sciences, University of Calabar, Calabar, Nigeria
[7] Water, Sanitation, Hygiene and Health Unit, World Health Organization, Avenue Appia 20, 1202 Geneva, Switzerland

* Correspondence to Clara MacLeod: clara.macleod@lshtm.ac.uk

For peer review only

ABSTRACT

Objectives: Hand hygiene is an important measure to prevent disease transmission in community settings, such as households, public spaces, workplaces, and schools. There exist various international guidelines with recommendations on how to improve hand hygiene in these settings, but no review to date has been conducted to summarise these recommendations and assess to what extent they are consistent and evidence-based.

Methods: To identify international guidelines with recommendations on hand hygiene in community settings, categorised as either domestic, public, and institutional, we performed electronic and grey literature searches and contacted expert organisations and individuals. Recommendations extracted from included guidelines were mapped to four areas related to hand hygiene: i) effective hand hygiene; ii) minimum requirements; iii) behaviour change; and iv) government measures. We assessed if recommendations were supported by peer-reviewed literature and checked their consistency and concordance across settings.

Results: We identified 51 guidelines published between 1999 and 2021 by multilateral agencies and international non-governmental organisations containing 923 recommendations. Handwashing with soap is consistently recommended as the preferred method for hand hygiene across all community settings. Most guidelines specifically recommend handwashing with plain soap and running water for at least 20 seconds; single-use paper towels for hand drying; and alcohol-based hand rub (ABHR) as a complement or alternative to handwashing. There are inconsistent and discordant recommendations for water quality for handwashing, affordable and effective alternatives to soap and ABHR, and the design of handwashing stations. Further, there are gaps in recommendations on soap and water quantity, behaviour change approaches, and government measures required for effective hand hygiene. Overall, less than 10% of recommendations are supported by evidence.

Conclusion: While current international guidelines consistently recommend handwashing with soap in domestic, public, and institutional settings, the lack of consistent, evidence-based recommendations may constrain global efforts to ensure effective hand hygiene across community settings.

For peer review only

Strengths and limitations of this study

- This is the first scoping review to synthesise current recommendations for hand hygiene community settings and assess to what extent they are consistent and evidence-based among international guidelines.
- We identified 51 guidelines with over 900 recommendations for hand hygiene in community settings published by multilateral agencies and international non-governmental organisations.
- The search was limited to guidelines published in English or French and therefore may have excluded relevant guidelines published in other languages.
- The quality of the included guidelines was not assessed, although we did consider the extent to which recommendations were based on evidence.

For peer review only

INTRODUCTION

Hand hygiene, including handwashing with soap and other methods such as alcohol-based hand rubs (ABHRs), is an important public health measure that can prevent the transmission of a range of diseases.[1] Handwashing with soap has been found to be a cost-effective intervention[2] that can reduce the risk of both diarrhoeal disease and acute respiratory infections (ARIs) by over 20%.[3–9] Handwashing with soap has also been linked to the reduction of certain neglected tropical diseases (NTDs), including trachoma and some soil-transmitted helminth infections.[10,11] Recently, handwashing with soap and the use of ABHRs were advised as one of the key control measures during the COVID-19 pandemic[12,13] and were found to be effective.[14]

This scoping review focuses on hand hygiene in non-healthcare settings, which we collectively refer to as "community settings". Using the definition set out in the Ottawa Charter, we consider settings as where "health is created and lived by people within the settings of their everyday life; where they learn, work, play and love",[15] and include domestic, public, and institutional settings. The practice of hand hygiene – and access to the facilities which enable this – is often limited in these community settings, particularly in low- and middle-income countries.[16] In the domestic setting, 30% of the global population does not have access to a basic handwashing facility with soap and water at home,[17,18] with three quarters of those living in low-income countries.[17] In institutional settings, an estimated 43% of schools worldwide do not have access to basic hand hygiene facilities[18] but there is limited data for other institutional settings, such as the workplace and prisons and places of detention, and public settings, such as markets, transportation hubs, and places of worship.[18]

Despite the international recognition of hand hygiene as a critical public health measure, a recent global assessment of government policies, planning, and financing for hygiene found that while the majority of surveyed countries reported having national policies for hand hygiene, less than 10% had sufficient financing to implement them.[19] Various international guidelines with recommendations on hand hygiene for non-healthcare settings exist[13,20–22] but it is unclear whether current international guidelines are comprehensive, consistent, and based on the most rigorous evidence available. This review aims to summarise current international recommendations for hand hygiene in community settings, identify areas of consistency and concordance, and assess whether recommendations are evidence-based.

METHODS

This review follows the six stages of the Arksey and O'Malley methodological framework for scoping reviews.[23–25] Expert consultation (stage 6) was integrated throughout the scoping review process[24] to obtain feedback on the scope and conceptual framework and to identify any additional guidelines beyond those identified during the electronic search. Our review is described according to the Preferred Reporting Items for Systematic reviews and Meta-Analyses extension for Scoping Reviews (PRISMA-ScR)[26] and a PRISMA-ScR checklist is included in the supplementary materials. (Table A1). The protocol was pre-registered with OSF Registries.[27]

Identifying the research question (stage 1)

To identify and refine the research question, a conceptual framework was developed for this review and built around three key concepts: i) hand hygiene, ii) non-healthcare settings, and iii) current international guidelines (Figure 1). In this review, we define hand hygiene as any action of hand cleansing for the purpose of removing or deactivating pathogens from hands.[28] Effective hand hygiene is defined as any practice which removes or deactivates pathogens from hands and thereby limits disease transmission.[28]

For peer review only

The first key concept in the conceptual framework – hand hygiene – covers four areas: effective hand hygiene, minimum requirements, behaviour change, and government measures (Figure 1). Effective hand hygiene refers to definitions and practices. Minimum requirements refer to the materials, services, and infrastructure required for effective hand hygiene. Behaviour change denotes the appropriate behavioural performances that promote and sustain effective hand hygiene. Government measures concern actions taken by governments to ensure effective hand hygiene, which are categorised according to an established framework[29] as follows: policy and strategy; institutional arrangements; sector financing; planning, monitoring, and review; capacity development; and equity.

The second key concept – community settings – is defined as settings where healthcare is not routinely delivered,[28] broadly spanning all places where people "learn, play, work and love"[15] and specifically including domestic, public, and institutional settings. As these settings may not exclusively refer to physical settings,[30] the review also includes recommendations for vulnerable populations (e.g., people experiencing homelessness) who may reside permanently or semi-permanently in public spaces. Nursing homes and long-term care facilities are also included in the review under institutional settings, as the boundary between healthcare and non-healthcare settings is often less clear.

The third concept – guideline – is defined as a published document where the primary purpose is to provide specific guidance, in the form of recommendations, towards a course of action. A recommendation is defined as a statement designed to assist a targeted actor to take informed decisions on whether, when, and how to undertake a specific action.[31] Our review is limited to international guidelines to identify generalisable recommendations of global relevance.

Identifying relevant studies (stage 2)

To identify relevant guidelines, we searched the WHO Institutional Repository for Information Sharing (IRIS) database and grey literature (Google search engine and websites of international organisations known to work on hand hygiene) (Table A2) using pre-specified search terms related to hand hygiene, non-healthcare settings, and guidelines (Table A3). Expert consultations were also conducted with 'Hand Hygiene for All' Initiative core partners (Table A4) to identify potentially relevant guidelines and reference lists of guidelines were hand-searched. The search was limited to English and French languages and publication date was restricted to 1990 onwards to identify current guidelines.

Study selection (stage 3)

Documents meeting the following criteria were included: i) international guideline, ii) offers one or more recommendations on hand hygiene, iii) targets one or more community setting, as defined in the conceptual framework, iv) published by an international NGO, multilateral agency, or public health agency, v) published in English or French, and vi) published between 1 January 1990 and 1 November 2021. The review excludes guidelines for humanitarian settings, as internationally agreed guidance on hand hygiene in humanitarian settings and complex emergencies is available through the Sphere standards for water, sanitation, and hygiene promotion.[32] Only the most recent versions of guidelines were included, with previous versions of the same guidelines excluded. Country-specific guidelines were also excluded.

All documents retrieved from electronic searches and expert consultations were transferred to Mendeley[33] for de-duplication. Inclusion was completed in two stages: (1) title and abstracts were screened for eligibility by one reviewer (CM); and (2) then full text for all potentially eligible documents were retrieved and independently assessed for inclusion by two reviewers (CM and LB). Disagreement between reviewers on inclusion was resolved through arbitration by a third reviewer (OC).

For peer review only

Charting the data (stage 4)

Guideline characteristics and recommendations from included guidelines were double-extracted by two reviewers (CM and LB) using a standardised data extraction template in MS Excel[34] and then cross-checked for accuracy. As with inclusion, a third reviewer (OC) provided arbitration if agreement on extraction could not be reached. The data extraction form included information on guideline characteristics, such as author, year of publication, target setting, and COVID-19 response, as well as 57 specific parameters related to the four areas of hand hygiene described in the conceptual framework (Figure 1). Recommendations for each parameter were extracted from included guidelines where possible.

Collating, summarising, and reporting the results (stage 5)

Guideline recommendations were first summarised for each parameter across community settings and then disaggregated by domestic, public, or institutional setting where relevant. Definitions and recommendations for hand hygiene were assessed for consistency, concordance, and whether supported by evidence. Recommendations were classified as consistent, fairly consistent, or inconsistent if they featured in 10 or more, four to nine, or less than four guidelines, respectively. Concordance is here defined as parameters with no consistent or fairly consistent recommendations at odds with each other. We also assessed whether extracted recommendations were supported by one of four types of evidence, including peer-reviewed literature, grey literature, other guideline, or other sources (e.g., programme documentation). A recommendation was considered evidence-based if the guideline provided a specific citation for the recommendation. Each evidence-based recommendation was then coded according to the type of evidence cited. Finally, evidence gaps were defined as parameters with very few recommendations (i.e., less than 10 recommendations, equivalent to less than 20% of guidelines providing a recommendation).

Patient and public involvement

There was no public or patient involvement in the course of this project.

RESULTS

Search results

Electronic searches were conducted on 15 November 2021 identifying 2,492 records (2,432 from the WHO IRIS database, 32 from Google, 28 from international agency websites) (Table A2) and a further 11 records identified through expert consultation. Following de-duplication, a total of 2,264 records were screened by title and abstract, and 125 documents were sought for retrieval for full-text screening, with one document not accessible. Finally, 51 guidelines are included in the review (Figure 2). The 73 documents excluded during the full-text review are listed in the Appendix with reasons for exclusion (Table A5).

Description of included guidelines

The 51 included guidelines were published in English between 1999 and 2021, with 38 published in 2020 or later and 31 providing COVID-19-specific guidance. Among the 51 included guidelines, 67% are published by multilateral agencies (WHO, UNICEF, UNHCR, and ILO), 23% by international NGOs, and 10% by the United States Centers for Disease Control and Prevention (US CDC) (Table A6). Most guidelines target public and institutional settings, while none exclusively target the domestic setting.

For peer review only

More specifically, 43% (n= 22) target the public setting, 43% (n= 22) the institutional setting, 4% (n= 2) the domestic and public setting, and 10% (n= 5) more than one setting (e.g., domestic, public, and/or institutional). Of the 22 guidelines for the public setting, 20 concern public spaces, and two concern vulnerable populations within public spaces (e.g., people experiencing unsheltered homelessness and people living in dense, informal settlements). For institutional settings, eight guidelines concern schools, six the workplace, four prisons and places of detention, three places of worship, and one long-term care facilities. Almost no guidelines, however, define a community setting. Overall, we extracted 923 recommendations from the 51 included guidelines (Table A7).

Recommendations for effective hand hygiene

Hand hygiene definitions

Only 10% of guidelines provide a clear definition for hand hygiene. Meanwhile, 75% of guidelines provide at least one recommendation on when to practice hand hygiene, referred to in our review as a 'key moment'. Different terms are used for key moments including 'key times' (14%), 'critical times' (8%), and 'key moments' (4%). Otherwise, guidelines either do not use a specific term for defining a key moment (49%) or do not recommend at least one key moment (25%).

Hand hygiene practices

There is agreement among guidelines on what constitutes effective hand hygiene across community settings, although there are gaps in recommendations on how to practice effective hand hygiene. Almost all guidelines (90%) recommend washing hands with soap and for a duration of at least 20 seconds (27%) (Table 1). In addition, 63% of guidelines recommend the use of ABHR as an alternative or complement to handwashing with soap, though very few guidelines recommend a duration for hand rubbing. Few guidelines provide specific recommendations on handwashing or hand rubbing technique (14%), with over half of these guidelines (60%, n= 4) referring to the WHO instructions for hand hygiene in healthcare settings.[28] Meanwhile, 24% of guidelines recommend including simple instructions on handwashing technique in hygiene promotion programmes.

Guidelines provide inconsistent recommendations on when to practice hand hygiene. 75% (n= 38) of guidelines specify a key moment for hand hygiene, though over 30 different individual key moments are recommended across guidelines. The most common individual key moments include before and after eating, after using the toilet, before and after preparing food, after blowing nose, coughing, or sneezing, and after touching public surfaces or objects (Table 1). The latter two feature most commonly among guidelines published during the COVID-19 pandemic. Fairly consistently recommended individual key moments include when entering or exiting the home or public space, after changing a child's diaper, and when hands are visibly dirty. However, almost all guidelines recommend clusters of key moments (i.e., individual key moments recommended together). The most common ones are 'after using the toilet' with 'before and after eating' (39%, n= 20); 'after using the toilet' with 'before and after preparing food' (24%, n= 12); and 'after using the toilet', with 'before and after eating', and 'after blowing nose, coughing, or sneezing' (22%, n= 11).

Recommendations for minimum requirements

Handwashing materials

Guidelines consistently recommend plain soap for handwashing and paper towels or electric dryers for hand drying. Most guidelines (65%) do not recommend a specific type of soap for handwashing, though some specifically recommend bar soap (24%), liquid soap (22%), or "soapy water" (e.g., home-

For peer review only

made mixture of powder or liquid soap, or bar soap shavings diluted in water) (16%) (Table 1). No guidelines specifically recommend antibacterial soap. For hand drying after handwashing, most guidelines recommend clean, single use paper towels (39%) and electric air-drying systems (22%). 8% of guidelines recommend a bin with disposal liners and lid for the waste management of paper towels.

Soap and water requirements for handwashing

While guidelines consistently recommend running water for handwashing with soap, there are gaps for soap and water quantity and discordant recommendations on water quality for handwashing. In terms of water services, almost half (43%) of guidelines recommend running water, 16% a piped water system connected to a tap, and 14% water trucking, storage, or manual refilling. Few guidelines recommend a minimum quantity of soap (14%) or water (18%) required for handwashing (Table 2). For water quality, 16% of guidelines state that water must be of drinking water quality in line with WHO guidelines.[35] In contrast, 8% state that it does not have to be of drinking water quality, though none specify a quantitative standard nor whether non-drinking quality water may conditionally be used if high quality water is not available. In addition, 10% of guidelines recommend that free residual chlorine must be greater than or equal to 0.5 mg/l after at least 30 minutes of contact time.

For peer review only

Table 1. Consistent and fairly consistent recommendations for hand hygiene in community settings.

Parameter	Recommendation	All guidelines (n)	Domestic setting (n)	Public setting (n)	Institutional setting (n)	Multiple settings (n)
Number of guidelines		51	2	24	22	5
**Effective hand hygiene**						
Key moments	Before and after eating	45% (23)	0% (0)	38% (9)	27% (14)	0% (0)
	After using the toilet	41% (21)	0% (0)	38% (9)	55% (12)	0% (0)
	Before and after preparing food	27% (14)	0% (0)	38% (9)	41% (9)	20% (1)
	After blowing nose, coughing, or sneezing	24% (12)	0% (0)	21% (5)	27% (6)	20% (1)
	After touching public surfaces or objects	24% (12)	0% (0)	12% (6)	18% (4)	40% (2)
	When entering/exiting buildings or home	14% (7)	0% (0)	17% (4)	5% (1)	40% (2)
	After changing a child's diaper	12% (6)	0% (0)	17% (4)	0% (0)	40% (2)
	When hands visibly dirty	10% (5)	0% (0)	13% (3)	9% (2)	0% (0)
	Before and after work	8% (4)	0% (0)	0% (0)	18% (4)	0% (0)
	After contact with animals	8% (4)	0% (0)	13% (3)	5% (1)	0% (0)
	Taking care of sick person	8% (4)	0% (0)	13% (3)	5% (1)	0% (0)
Handwashing duration	At least 20 seconds	27% (14)	0% (0)	21% (5)	36% (8)	20% (1)
**Minimum requirements for effective hand hygiene**						
**Materials for effective hand hygiene**						
Soap	Soap	65% (33)	100% (2)	50% (12)	82% (18)	60% (3)
	Bar soap	24% (12)	0% (0)	14% (7)	14% (3)	40% (2)
	Liquid soap	22% (11)	0% (0)	14% (7)	14% (3)	20% (1)
	Soap water (e.g., powder, liquid, or bar soap shavings diluted in water)	16% (8)	0% (0)	17% (4)	5% (1)	60% (3)
ABHR	ABHR with at least 60% alcohol	35% (18)	0% (0)	16% (8)	45% (10)	0% (0)
	ABHR, no alcohol percentage	22% (11)	50% (1)	17% (4)	23% (5)	20% (1)
Hand drying	Clean, single use paper towels	39% (20)	0% (0)	14% (7)	59% (13)	0% (0)
	Air-drying system	22% (11)	0% (0)	17% (4)	14% (7)	0% (0)
	Bin with disposable liners and lid	8% (4)	0% (0)	4% (1)	14% (3)	0% (0)
**Alternative materials**						
Other materials	Ash	22% (11)	0% (0)	12% (6)	14% (3)	40% (2)
Conditional recommendations	ABHR where soap and water not available	18% (9)	0% (0)	4% (1)	36% (8)	0% (0)
	Ash and water if ABHR or soap not available	14% (7)	0% (0)	13% (3)	14% (3)	20% (1)

For peer review only - http://bmjopen.bmj.com/site/about/guidelines.xhtml

	ABHR if hands not visibly soiled	10% (5)	0% (0)	8% (2)	14% (3)	0% (0)
**Water requirements**						
Water services	Running water	43% (22)	0% (0)	16% (8)	55% (12)	40% (2)
	Piped water system connected to tap	16% (8)	0% (0)	13% (3)	18% (4)	20% (1)
	Water trucking, storage, or manual refilling	14% (7)	0% (0)	13% (3)	14% (3)	20% (1)
Alternative water sources for handwashing	Rainwater	8% (4)	0% (0)	17% (4)	0% (0)	0% (0)
Wastewater management	Drainage system	16% (8)	0% (0)	8% (2)	23% (5)	20% (1)
	Covered soakaway pit	12% (6)	0% (0)	21% (5)	0% (0)	20% (1)
**Hand hygiene stations**						
Handwashing station	Washbasin	16% (8)	0% (0)	13% (3)	18% (4)	20% (1)
	Bucket or container connected to tap	14% (7)	50% (1)	17% (4)	5% (1)	40% (2)
	Tippy tap	12% (6)	0% (0)	4% (1)	14% (3)	40% (2)
	Foot-pedal or hand-operated system	8% (4)	0% (0)	8% (2)	5% (1)	20% (1)
Location of handwashing stations	Key entry/exit points	(14)	0% (0)	50% (12)	5% (1)	20% (1)
	Close proximity to toilets	(14)	0% (0)	21% (5)	14% (7)	40% (2)
	food preparation and eating areas	(9)	0% (0)	8% (2)	27% (6)	20% (1)
Accessibility	Accessible for all users and vulnerable groups	12% (6)	0% (0)	13% (3)	5% (1)	40% (2)
	Height of soap and water taps appropriate for children, elderly, and disabled	10% (5)	0% (0)	13% (3)	5% (1)	20% (1)
	Age-appropriate handwashing stations	8% (4)	0% (0)	0% (0)	18% (4)	0% (0)
Design considerations	Theft resistance	16% (8)	0% (0)	17% (4)	14% (3)	20% (1)
	Water-saving designs/technologies	12% (6)	0% (0)	13% (3)	9% (2)	20% (1)
COVID-19 adaptations	Taps that limit cross-contamination	16% (8)	0% (0)	14% (7)	0% (0)	20% (1)
	1-2m spacing between handwashing stations	12% (6)	0% (0)	17% (4)	5% (1)	20% (1)
	Disinfect taps regularly	10% (5)	0% (0)	17% (4)	0% (0)	20% (1)
	Towels for opening/closing taps	8% (4)	0% (0)	13% (3)	5% (1)	0% (0)
Availability of materials	Locally available materials for handwashing	10% (5)	0% (0)	8% (2)	9% (2)	20% (1)
**Behaviour change**						
Behaviour change techniques[1]	Prompts and cues	12% (6)	0% (0)	13% (3)	14% (3)	0% (0)
	Habit formation	8% (4)	0% (0)	13% (3)	5% (1)	0% (0)
Content of behaviour change messaging	Doable instructions with proper steps	24% (12)	0% (0)	12% (6)	27% (6)	0% (0)
	Messages that target motivation	10% (5)	0% (0)	8% (2)	14% (3)	0% (0)

For peer review only - http://bmjopen.bmj.com/site/about/guidelines.xhtml

Delivery channels	Visual reminders	31% (16)	0% (0)	12% (6)	36% (8)	40% (2)
	Mass communication	24% (12)	0% (0)	14% (7)	9% (2)	60% (3)
	Small group activities	18% (9)	0% (0)	12% (6)	9% (2)	20% (1)
	Interpersonal communication	10% (5)	0% (0)	17% (4)	0% (0)	20% (1)
Formative research	Identify determinants of target behaviour within target population	8% (4)	0% (0)	4% (1)	0% (0)	60% (3)

[1] Using a typology of behaviour change techniques developed by Michie et al. (2013)[36]

For peer review only

Table 2. Parameters with gaps in recommendations (i.e., fewer than 10 recommendations, equivalent to less than 20% of guidelines providing a recommendation).

**Parameters with gaps in recommendations[1]**	**Percentage (%) of guidelines that provide a recommendation (n)**
**Effective hand hygiene**	
No definition of hand hygiene	4% (2)
No definition of safe hand hygiene	6% (3)
Handwashing knowledge	16% (8)
Hand rubbing duration	6% (3)
**Minimum requirements for effective hand hygiene**	
**Soap and water requirements for handwashing**	
Quantity of soap	14% (7)
Quantity of water	18% (9)
**Hand hygiene stations**	
Soap supply	6% (3)
Functionality	10% (5)
Cost and affordability	14% (7)
Durability	16% (8)
Operation and maintenance – responsibility	16% (8)
Operation and maintenance – actions	18% (9)
Spacing and number of users per handwashing station	18% (9)
**Behaviour and behaviour change**	
Frequency of behaviour change interventions	4% (2)
Behaviour change approaches	18% (9)
**Government measures**	
Sector policy and strategy	0% (0)
Sector financing	0% (0)
Capacity development	2% (1)
Equity	2% (1)
Planning, monitoring, and review	4% (2)
Institutional arrangements	10% (5)

[1] Parameters with less than 20% of guidelines that provide a recommendation

Alternative materials for hand hygiene

There are consistent recommendations for ABHR as an alternative material for hand hygiene. Of the guidelines that recommend ABHR, 35% (n= 18) recommend ABHR with at least 60% alcohol, 22% (n= 11) do not specify an alcohol percentage, and only 6% (n= 3) recommend an alcohol percentage of at least 70%. In addition, 18% of guidelines recommend ABHR where soap and water are not available and 10% (n= 5) only if hands are not visibly soiled.

There are discordant recommendations for the use of ash. 22% of guidelines recommend ash as an alternative material to soap for handwashing. In addition, 14% recommend ash if ABHR or soap are not available. However, 14% of guidelines advise against the use of ash or other products, such as soil, sand, mud, or water alone. In addition, while some guidelines (8%) recommend 0.05% chlorine solution, one guideline advises against it.

When disaggregated by setting, ABHR is most consistently recommended in public and institutional settings where it may meet larger and more frequent demand than handwashing stations. Similarly, recommendations for the conditional use of ABHR where soap and water are not available feature the most in the institutional setting, particularly in the workplace and schools. Ash is most commonly

For peer review only

recommended in the public setting, which includes low-resource and water-scarce settings where soap and water may not be available.

Hand hygiene stations

Overall, there are inconsistent recommendations on hand hygiene facilities and their location, as well as gaps. Guidelines recommend washbasins (e.g., ceramic, cement, or plastic) (16%), a bucket or container connected to tap (14%), and tippy taps (12%) for handwashing stations across all settings (Table 1). Guidelines specify 16 different locations for handwashing stations, which include by the entrance and exit of public spaces and buildings (e.g., restaurants, shops, markets, places of worship, train and bus stations) (27%), in close proximity to toilets (27%), and next to food preparation and eating areas (18%). Guidelines also mention placing hand hygiene stations, such as ABHR dispensers, at key entry and exit points of public spaces and buildings (14%). There are inconsistent recommendations on the optimal spacing and number of users per handwashing station and none for ABHR dispensers. Gaps in recommendations include those for hand hygiene materials and product supply chains, cost and affordability, functionality, durability, and hand hygiene station operation and maintenance responsibilities and actions (Table 2).

Recommendations on the location of handwashing stations vary slightly by setting. Close proximity to toilets is consistently recommended for both public and institutional settings (10% and 14%, respectively). By the entrance and exit of public spaces is most consistently recommended for public settings (24%). Next to food preparation and eating areas (12%) is most consistently recommended for institutional settings, such as schools and the workplace.

Hand hygiene station access and adaptations

Guidelines recommend hand hygiene stations that are accessible for all, including people with disabilities and older adults, and adapted for pandemic response. For example, 12% of guidelines recommend that hand hygiene stations be accessible for all users and vulnerable groups. In addition, 10% recommend that the height of soap and water taps be appropriate for access by children, the elderly, and disabled (e.g., 500-700 mm basin height for children and 850 mm basin height for wheelchair access) (Table 1). 45% (n= 23) of guidelines recommend COVID-19 adaptations for hand hygiene stations, such as taps that limit cross contamination (16%), one to two meters spacing between stations (12%), regular tap disinfection (10%), and towels for opening and closing taps (8%). Other adaptations include theft resistance (e.g., attaching soap or other movable pieces to the station) (16%) and water-saving designs (e.g., low-flow handwashing stations) (12%).

Recommendations for behaviour change

Overall, there are few recommendations related to hand hygiene behaviour change, though there are consistent recommendations on behaviour change messaging and delivery channels. For behaviour change messaging, some guidelines (10%) recommend messages that target "motives"[37] (Table 1). In terms of delivery channels, 31% of guidelines recommend using visual reminders (e.g., signs, posters, or leaflets) and 24% mass communication (e.g., radio, social media, or SMS) to deliver behaviour change messages. Prompts, cues and habit formation are fairly consistently recommended as behaviour change techniques.[36] Formative research for behaviour change programmes is recommended by 10% (n= 5) of guidelines, though only 8% (n= 4) specifically mention undertaking formative research to identify behavioural determinants among the target population. Guidelines provide inconsistent recommendations on behaviour change approaches (18%), determinants of hand hygiene to target for interventions (22%), and behaviour change models or frameworks (33%). Lastly,

For peer review only

there are gaps in recommendations on frequency (i.e., how often an intervention should be delivered) of behaviour change interventions and approaches (4%) (Table 2).

Recommendations for government measures

Overall, few guidelines (22%, n= 11) provide a recommendation on government measures. Nonetheless, recommendations for sector policy and strategy include promoting local soap production and fostering public-private partnerships for handwashing (4%). For institutional arrangements, 4% of guidelines suggest identifying ways of cross-sectoral collaboration for hand hygiene, while other recommendations centre on engaging communities, the private sector, and civil society for the delivery of water, sanitation, and hygiene (WASH) services. On planning, monitoring, and review, 4% of guidelines recommend supporting or reinforcing existing monitoring systems or creating a government-led national monitoring system, in line with global hygiene indicators.

Evidence-based recommendations

Most recommendations are not supported by evidence (Figure 3). Of the 923 extracted recommendations, only 7% (n= 68) are supported by at least one of the four types of evidence identified for the review. In addition, only 2% (n= 18) are supported by peer-reviewed literature, which are mainly for alternative hand hygiene materials, such as ash, sand, and soil, or alternative water sources for handwashing, such as cooking water, laundry water, bathwater, and seawater. Nonetheless, the cited peer-reviewed literature are individual studies, and not systematic reviews.

DISCUSSION

We identified 51 guidelines published between 1999 and 2021 by various international agencies covering a range of community settings. Most guidelines target the public and institutional setting, while surprisingly none exclusively target the domestic setting. Overall, community settings are not clearly defined among the guidelines, which presents an opportunity for future normative guidelines to establish a clear and common definition, especially as it relates to hand hygiene. Overall, no guidelines comprehensively address hand hygiene across domestic, public, and institutional settings, and very few recommendations are evidence-based, highlighting a gap in global normative guidance on hand hygiene in community settings.

Effective hand hygiene

There is agreement among guidelines on what constitutes effective hand hygiene, however, inconsistencies and gaps remain as to when and how to practice hand hygiene. Current recommendations for handwashing with soap reflect the literature that suggests that handwashing with soap is an effective means for preventing the transmission of a range of diseases.[3–9] Regarding when to practice hand hygiene, future guidelines could focus key moments on those most likely to interrupt the transmission of infectious diseases in domestic, public, and institutional settings. These might include after using the toilet to reduce the transmission of diarrhoea-causing pathogens,[9] and after touching high-contact surfaces or after coughing or sneezing to reduce the risk of respiratory infections.[3] Lastly, gaps in recommendations on handwashing and hand rubbing technique present an opportunity for future guidelines to recommend the optimal technique for reducing bacterial load on hands.

Minimum requirements

For peer review only

Consistent recommendations for the use of soap and water and ABHR suggest the widespread acceptability of these materials for effective hand hygiene across community settings. Current recommendations for the use of plain soap are consistent with findings from a systematic review that suggest that plain soap is more effective than antibacterial soap at removing or inactivating pathogens on hands in community settings.[38] Recommendations for running water are equally relevant as unreliable water supply negatively affects households' ability to perform hand hygiene.[39] Gaps in recommendations on minimum quantities of soap and water required for effective hand hygiene are important for future guidelines to address. The recommended use of ABHR with at least 60% alcohol is in line with several studies which have found that ABHRs with an alcohol concentration between 60 and 80% are more effective at killing germs than those with a lower alcohol percentage, particularly in clinical settings.[40,41] Although ABHR can still inactivate many types of microbes when used correctly, evidence suggests that soap and running water are more effective at removing certain types of pathogens that may be present on hands.[42] The WHO Guidelines on Hand Hygiene in Health Care, for example, recommend ABHR as the preferred method for routine hand hygiene in healthcare settings when hands are not visibly soiled, as it enables more frequent hand hygiene.[28] Similarly, in community settings, ABHR may be suitable in contexts where frequent hand hygiene is necessary, such as transport hubs and entrances or exits to public spaces and buildings.[43] However, in certain domestic, public, and institutional settings, such as the household or schools, where hands may become more soiled, ABHR may be less likely to effectively inactivate microbes.[40,41] As per current international guidelines, handwashing with soap may therefore be prioritised in these settings, with ABHR as a suitable complement or alternative where frequent hand hygiene is required. Finally, because the transmission of germs is more likely to occur to and from wet hands, hand drying is an essential component of effective hand hygiene, especially for handwashing.[44] Current recommendations for hand drying are consistent with those in the WHO Guidelines on Hand Hygiene in Health Care, which recommend that hands should ideally be dried with individual paper towels, otherwise with air dryers.[28] While there is mixed evidence for the most effective hand drying method,[45] the WHO recommendations are based on findings that suggest that paper towels may effectively prevent re-contamination of hands, while also lowering the risk of spreading pathogens through the air compared to electric air dryers.[44]

Discordant recommendations for minimum requirements suggest the need to leverage further research to determine the optimal water quality for handwashing and effectiveness of alternative materials for hand hygiene where soap and ABHR are not widely available. Limited evidence suggests that the use of non-potable water with low-to-moderate levels of *E. coli* contamination may still be effective for handwashing,[46] which may be promising for areas where it is difficult to regularly treat water or where there is intermittent water supply that is prone to contamination. Similarly, two studies found that drinkable water may not be needed for handwashing with soap.[47,48] Nevertheless, the WHO Healthcare Guidelines recommend washing hands with clean, running water whenever possible.[28] While there are discordant recommendations for the use of ash, there is uncertain evidence whether this stops or reduces the spread of pathogens compared to hand cleansing with soap, mud, soil, or no hand cleansing.[49] Future guidelines may consider the efficacy of hand hygiene products along with their availability and acceptability in domestic, public, and institutional settings to make relevant recommendations, particularly in water-scarce regions or settings where there is limited access to soap or ABHR. For example, soapy water may be a promising low-cost and effective alternative to bar soap in settings where bar or liquid soap is unaffordable.[50] One interim guideline on hand hygiene practices in low-resource settings recommends the use of friction-generating materials where clean, running water, soap, or ABHR are not available.[51]

Current inconsistent recommendations for hand hygiene facilities and their placement may limit the practice of effective hand hygiene. Sustaining hand hygiene behaviour change requires consistent access to functional hand hygiene stations at key locations[52,53] and diverse infrastructure is

For peer review only - http://bmjopen.bmj.com/site/about/guidelines.xhtml

recommended with varying costs. Nonetheless, the appropriateness of these recommendations is likely to depend on the local availability and affordability of materials. One guideline, for example, provides technical recommendations for permanent and semi-permanent handwashing facilities in public places and buildings, focusing on the sustainability and equitable access of these facilities.[20] Similarly, future guidelines may prioritise the accessibility, affordability, and sustainability of materials and infrastructure for hand hygiene in domestic, public, and institutional settings to address current inconsistent and discordant recommendations. The accessibility and sustainability of hand hygiene stations are particularly important to ensure that they are inclusive and kept functional and well-stocked beyond their installation.[20]

Behaviour change

The gaps in recommendations related to behaviour change suggest the need for guidance based on established behavioural theory and existing evidence. There are some recommendations for behaviour change, though without clear steps on how to develop, implement, and sustain hand hygiene behaviour change interventions. Future guidelines may benefit from leveraging well-established behavioural frameworks and theories[54–56] to make recommendations as to how to develop effective, locally-appropriate strategies beyond information-focused communication. In addition, while most behaviour change theories and frameworks recommended amongst the guidelines note the importance of robust formative research, very few guidelines recommend undertaking formative research. Yet, formative research plays a key role in adapting hand hygiene behaviour change programmes to high-risk populations and target settings.[57]

Government measures

The significant gaps in recommendations on government measures underscore the current lack of normative standards to guide national governments on the planning, delivery, financing, and monitoring of effective hand hygiene. Indicators also suggest inadequate planning and insufficient funding for hand hygiene among national governments globally.[19] Future guidelines may therefore consider prioritising government measures to support countries in responding to and preventing public health crises, such as the COVID-19 pandemic. Future guidelines may also focus recommendations on hand hygiene monitoring and reporting to improve comparison of hand hygiene indicators within and between countries.

Strengths and limitations

This review has three main limitations. First, as a scoping review we did not systematically assess the quality of the included guidelines although we did, for example, consider aspects such as the extent to which recommendations were based on evidence. In addition, included guidelines covered WASH and public health topics beyond hand hygiene, so it was therefore not necessarily relevant to assess the whole guidelines for quality, but rather focus on the robustness of the specific recommendations for hand hygiene. Second, the search was limited to guidelines published in English or French and therefore may have excluded relevant guidelines published in other languages. Third, while recommendations were summarised across domestic, public, and institutional settings, there were often too few recommendations for each setting to assess consistency and concordance. Still, with 51 guidelines providing over 900 recommendations for hand hygiene in community settings, findings from this review highlight significant gaps and inconsistencies that future guidelines may seek to prioritise.

CONCLUSION

For peer review only

This review identified 51 current international guidelines for hand hygiene in community settings providing 923 recommendations. Nonetheless, there are several important areas of discordance and significant gaps in the recommendations among these guidelines. Furthermore, very few recommendations are supported by any qualifying evidence. The COVID-19 pandemic led to numerous national, regional, and international efforts to improve effective hand hygiene in domestic, public, and institutional settings, such as households, public spaces, workplaces, and schools, but the lack of clear, evidence-based recommendations may limit progress in this important area of public health.

For peer review only

Contributors

CM, LB, BC, CC, KC, JC, RD, REN, BG, JEM, and OC informed the study protocol. CM carried out the database and grey literature search with input from OC and JEM. JEM, OC, BG, and CM carried out expert consultations. CM and LB screened the retrieved articles for inclusion and extracted the data with input from OC. CM led on data analysis and CM, LB, OC, and JEM led on the presentation of results with inputs from co-authors. CM, LB, JEM, and OC led on writing the manuscript with input from all co-authors. OC, JEM, and BG provided overall supervision, leadership, and advice. The first author (CM) accepts responsibility for the work.

Funding

This research was funded by the World Health Organization (grant number N/A), UK Foreign, Commonwealth, and Development Office (grant number N/A) and London School of Hygiene and Tropical Medicine (grant number N/A).

Competing interests

The authors declare no competing interests.

Patient and public involvement

Patients and/or the public were not involved in the design, or conduct, or reporting, or dissemination plans of this research.

Patient consent for publication

Not applicable.

Ethics approval

This research did not require institutional review board approval as the data were publicly available and collected from existing online databases and search engines. This research did not involve any human subjects.

Data availability statement

All data relevant to the study are included in the article or uploaded as supplementary information.

For peer review only

References

(1) Burton, M.; Cobb, E.; Donachie, P.; Judah, G.; Curtis, V.; Schmidt, W. P. The Effect of Handwashing with Water or Soap on Bacterial Contamination of Hands. *Int. J. Environ. Res. Public Health* **2011**, *8* (1), 97–104. https://doi.org/10.3390/ijerph8010097.

(2) Cairncross, S.; Valdamis, V. Water Supply, Sanitation, and Hygiene Promotion. In *Disease Control Priorities in Developing Countries*; World Bank: Washington, 2006; pp 771–792.

(3) Freeman, M.; Stocks, M.; Cumming, O.; Jeandron, A.; Higgins, J.; Wolf, J.; Prüss-Ustün, A.; Bonjour, S.; Hunter, P.; Fewtrell, L.; Curtis, V. Hygiene and Health: Systematic Review of Handwashing Practices Worldwide and Update of Health Effects. *Trop. Med. Int. Health* **2014**, *19* (8), 906–916. https://doi.org/10.1111/TMI.12339.

(4) Rabie, T.; Curtis, V. Handwashing and Risk of Respiratory Infections: A Quantitative Systematic Review. *Trop. Med. Int. Heal.* **2006**, *11* (3), 258–267. https://doi.org/10.1111/j.1365-3156.2006.01568.x.

(5) Aiello, A. E.; Coulborn, R. M.; Perez, V.; Larson, E. L. Effect of Hand Hygiene on Infectious Disease Risk in the Community Setting: A Meta-Analysis. *Am. J. Public Health* **2008**, *98* (8), 1372–1381. https://doi.org/10.2105/AJPH.2007.124610.

(6) Prüss-Ustün, A.; Wolf, J.; Bartram, J.; Clasen, T.; Cumming, O.; Freeman, M.; Gordon, B.; Hunter, P.; Medlicott, K.; Johnston, R. Burden of Disease from Inadequate Water, Sanitation and Hygiene for Selected Adverse Health Outcomes: An Updated Analysis with a Focus on Lowand Middle-Income Countries. *Int. J. Hyg. Environ. Health* **2019**, *222*, 765–777.

(7) Mbakaya, B. C.; Lee, P. H.; Lee, R. L. T. Hand Hygiene Intervention Strategies to Reduce Diarrhoea and Respiratory Infections among Schoolchildren in Developing Countries: A Systematic Review. *Int. J. Environ. Res. Public Health* **2017**, *14* (4), 1–14. https://doi.org/10.3390/ijerph14040371.

(8) Wolf, J.; Hubbard, S.; Brauer, M.; Ambelu, A.; Arnold, B. F.; Bain, R.; Bauza, V.; Brown, J.; Caruso, B. A.; Clasen, T.; Colford, J. M.; Freeman, M. C.; Gordon, B.; Johnston, R. B.; Mertens, A.; Prüss-Ustün, A.; Ross, I.; Stanaway, J.; Zhao, J. T.; Cumming, O.; Boisson, S. Effectiveness of Interventions to Improve Drinking Water, Sanitation, and Handwashing with Soap on Risk of Diarrhoeal Disease in Children in Low-Income and Middle-Income Settings: A Systematic Review and Meta-Analysis. *Lancet* **2022**, *400* (10345), 48–59. https://doi.org/10.1016/S0140-6736(22)00937-0.

(9) Ejemot-Nwadiaro, R.; Ehiri, J.; Arikpo, D.; Meremikwu, M.; Critchley, J. Hand Washing Promotion for Preventing Diarrhoea (Review). *Cochrane Database Syst. Rev.* **2015**, *2015* (9). https://doi.org/10.1002/14651858.CD004265.pub3.

(10) Stocks, M. E.; Ogden, S.; Haddad, D.; Addiss, D. G.; McGuire, C.; Freeman, M. C. Effect of Water, Sanitation, and Hygiene on the Prevention of Trachoma: A Systematic Review and Meta-Analysis. *PLoS Med.* **2014**, *11* (2). https://doi.org/10.1371/journal.pmed.1001605.

(11) Strunz, E. C.; Addiss, D. G.; Stocks, M. E.; Ogden, S.; Utzinger, J.; Freeman, M. C. Water, Sanitation, Hygiene, and Soil-Transmitted Helminth Infection: A Systematic Review and Meta-Analysis. *PLoS Med.* **2014**, *11* (3). https://doi.org/10.1371/journal.pmed.1001620.

(12) World Health Organization. *United Nations Children's Fund (2020). Water, Sanitation, Hygiene, and Waste Management for SARS-CoV-2, the Virus That Causes COVID-19: Interim Guidance*; Geneva, Switzerland, 2020.

(13) World Health Organization. *Recommendations to Member States to Improve Hand Hygiene Practices to Help Prevent the Transmission of the COVID-19 Virus: Interim Guidance, 1 April 2020*; World Health Organization, 2020.

(14) Talic, S.; Shah, S.; Wild, H.; Gasevic, D.; Maharaj, A.; Ademi, Z.; Li, X.; Xu, W.; Mesa-Eguiagaray, I.; Rostron, J.; Theodoratou, E.; Zhang, X.; Motee, A.; Liew, D.; Ilic, D. Effectiveness of Public Health Measures in Reducing the Incidence of Covid-19, SARS-CoV-2 Transmission, and Covid-19 Mortality: Systematic Review and Meta-Analysis. *BMJ* **2021**, 375.

https://doi.org/https://doi.org/10.1136/bmj-2021-068302.
(15) World Health Organization. The Ottawa Charter for Health Promotion. WHO: Geneva, Switzerland 1986.
(16) Brauer, M.; Zhao, J.; Bennitt, F.; Stanaway, J. Global Access to Handwashing: Implications for COVID-19 Control in Low-Income Countries. *Environ. Health Perspect.* **2020**, *128* (5). https://doi.org/https://doi.org/ 10.1289/EHP7200.
(17) World Health Organization; UNICEF. *Progress on Household Drinking Water, Sanitation and Hygiene 2000-2020: Five Years into the SDGs*; Geneva, Switzerland, 2020.
(18) World Health Organization; UNICEF. *Progress on Drinking Water, Sanitation and Hygiene in Schools: Special Focus on Covid-19*; 2020.
(19) World Health Organization. Hygiene - UN-Water GLAAS Findings on National Policies, Plans, Targets and Finance. *World Health Organization*. Licence: CC BY-NC-SA 3.0 IGO: Geneva, Switzerland 2020.
(20) WaterAid. *Technical Guide for Handwashing Facilities in Public Places and Buildings*; 2020.
(21) ILO. *Hand Hygiene at the Workplace: An Essential Occupational Safety and Health Prevention and Control Measure against COVID-19*; Geneva, Switzerland, 2020.
(22) ICRC. *Water, Sanitation, and Hygiene and Habitat in Prisons: Supplementary Guidance*; Geneva, Switzerland, 2012.
(23) Arksey, H.; O'Malley, L. Scoping Studies: Towards a Methodological Framework. *Int. J. Soc. Res. Methodol.* **2005**, *8* (1), 19–32. https://doi.org/10.1080/1364557032000119616.
(24) Levac, D.; Colquhoun, H.; O'Brien, K. Scoping Studies: Advancing the Methodology. *Implement. Sci.* **2010**, *5* (69). https://doi.org/10.1017/cbo9780511814563.003.
(25) Westphaln, K. K.; Regoeczi, W.; Masotya, M.; Vazquez-Westphaln, B.; Lounsbury, K.; McDavid, L.; Lee, H. N.; Johnson, J.; Ronis, S. D. From Arksey and O'Malley and Beyond: Customizations to Enhance a Team-Based, Mixed Approach to Scoping Review Methodology. *MethodsX* **2021**, *8*, 101375. https://doi.org/10.1016/j.mex.2021.101375.
(26) Tricco, A.; Lillie, E.; Zarin, W.; O'Brien, K.; Colquhoun, H.; Levac, D.; Moher, D.; Peters, M.; Horsley, T.; Weeks, L.; Hempel, S.; Akl, E.; Chang, C.; McGowan, J.; Stewart, L.; Hartling, L.; Aldcroft, A.; Wilson, M.; Garritty, C.; Lewin, S.; Godfrey, C.; Macdonald, M.; Langlois, E.; Soares-Weiser, K.; Moriarty, J.; Clifford, T.; Tunçalp, O.; Straus, S. PRISMA Extension for Scoping Reviews (PRISMA-ScR): Checklist and Explanation. *Ann. Intern. Med.* **2018**, *169* (7), 467–473. https://doi.org/10.7326/M18-0850.
(27) MacLeod, C.; Braun, L.; Chipungu, J.; Chidziwisano, K.; Ejemot-Nwadiaro, R.; Dreibelbis, R.; Gordon, B.; Mills, J. E. Guideline recommendations for hand hygiene in community settings: scoping review protocol https://osf.io/z2gvs/?view_only=95516bea2202437d8fbea11be17fcae7.
(28) World Health Organization. *WHO Guidelines on Hand Hygiene in Health Care*; Geneva, Switzerland, 2009.
(29) Sanitation and Water for All. Building Blocks https://www.sanitationandwaterforall.org/about/our-work/priority-areas/building-blocks (accessed Jun 15, 2022).
(30) MacQueen, K.; McLellan, E.; Metzger, D.; Kegeles, S.; Strauss, R.; Scotti, R.; Blanchard, L.; Trotter, R. What Is Community? An Evidence-Based Definition for Participatory Public Health. *Am. J. Public Health* **2001**, *91* (12).
(31) World Health Organization. WHO Guidelines https://www.who.int/publications/who-guidelines#:~:text=A WHO guideline is defined,practice or public health policy. (accessed Jun 15, 2022).
(32) Sphere Association. The Sphere Handbook: Humanitarian Charter and Minimum Standards in Humanitarian Response (Fourth Edition). Geneva, Switzerland 2018.
(33) Elsevier. Mendeley. London, United Kingdom.
(34) Microsoft. Microsoft Excel Spreadsheet Software. Redmon, VA, USA.

For peer review only

(35) World Health Organization. Guidelines for Drinking-Water Quality, 4th Edition. 4th ed. World Health Organization 2017.

(36) Michie, S.; Richardson, M.; Johnston, M.; Abraham, C.; Francis, J.; Hardeman, W.; Eccles, M.; Cane, J.; Wood, C. The Behavior Change Technique Taxonomy (v1) of 93 Hierarchically Clustered Techniques: Building an International Consensus for the Reporting of Behavior Change Interventions. *Ann. Behav. Med.* **2013**, *46* (1), 81–95. https://doi.org/10.1007/s12160-013-9486-6.

(37) Curtis, V. A.; Danquah, L. O.; Aunger, R. V. Planned, Motivated and Habitual Hygiene Behaviour: An Eleven Country Review. *Health Educ. Res.* **2009**, *24* (4), 655–673. https://doi.org/10.1093/her/cyp002.

(38) Aiello, A. E.; Larson, E. L.; Levy, S. B. Consumer Antibacterial Soaps: Effective or Just Risky? *Clin. Infect. Dis.* **2007**, *45* (SUPPL. 2). https://doi.org/10.1086/519255.

(39) Kumpel, E.; Billava, N.; Nayak, N.; Ercumen, A. Water Use Behaviors and Water Access in Intermittent and Continuous Water Supply Areas during the COVID-19 Pandemic. *J. Water Health* **2022**, *20* (1), 139–148. https://doi.org/10.2166/WH.2021.184.

(40) Todd, E.; Michaels, B.; Holah, J.; Smith, D.; Grieg, J.; Bartleson, C. Outbreaks Where Food Workers Have Been Implicated in the Spread of Foodborne Disease. Part 10. Alcohol-Based Antiseptics for Hand Disinfection and a Comparison of Their Effectiveness with Soaps. *J. Food Prot.* **2010**, *73* (11), 2128–2140.

(41) Charbonneau, D.; Ponte, J.; Kochanowski, B. A Method of Assessing the Efficacy of Hand Sanitizers: Use of Real Soil Encountered in the Food Service Industry. *J. Food Prot.* **2000**, *63* (4), 495–501.

(42) Burton, M.; Cobb, E.; Donachie, P.; Judah, G.; Curtis, V.; Schmidt, W. The Effect of Handwashing with Water or Soap on Bacterial Contamination of Hands. *Int. J. Environ. Res. Public Health* **2011**, *8* (1), 97–104.

(43) Howard, G.; Bartram, J.; Brocklehurst, C.; Colford, J. M.; Costa, F.; Cunliffe, D.; Dreibelbis, R.; Eisenberg, J. N. S.; Evans, B.; Girones, R.; Hrudey, S.; Willetts, J.; Wright, C. Y. COVID-19: Urgent Actions, Critical Reflections and Future Relevance of "WaSH": Lessons for the Current and Future Pandemics. *J. Water Health* **2020**, *18* (5), 613–630. https://doi.org/10.2166/wh.2020.162.

(44) Huang, C.; Ma, W.; Stack, S. The Hygienic Efficacy of Different Hand-Drying Methods: A Review of the Evidence. *Mayo Clin. Proc.* **2012**, *87* (8), 791–798. https://doi.org/10.1016/j.mayocp.2012.02.019.

(45) Gustafson, D.; Vetter, E.; Larson, D.; Ilstrup, D.; Maker, M.; Thompson, R.; Cockerill, F. Effects of 4 Hand-Drying Methods for Removing Bacteria from Washed Hands: A Randomized Trial. *Mayo Clin. Proc.* **2000**, *75* (7), 705–708.

(46) Verbyla, M. E.; Pitol, A. K.; Navab-Daneshmand, T.; Marks, S. J.; Julian, T. R. Safely Managed Hygiene: A Risk-Based Assessment of Handwashing Water Quality. *Environ. Sci. Technol.* **2019**, *53* (5), 2852–2861. https://doi.org/10.1021/acs.est.8b06156.

(47) Hoque, B. Handwashing Practices and Challenges in Bangladesh. *Int. J. Environ. Health Res.* **2004**, *13* (1), S81–S87. https://doi.org/https://doi.org/10.1080/0960312031000102831.

(48) Luby, S.; Agboatwalla, M.; Painter, J.; Altaf, A.; Billhimer, W.; Hoekstra, R. Effect of Intensive Handwashing Promotion on Childhood Diarrhea in High-Risk Communities in Pakistan: A Randomized Controlled Trial. *J. Am. Med. Assoc.* **2004**, *291* (21), 2547–2554. https://doi.org/10.1001/jama.291.21.2547.

(49) Paludan-Müller AS, Boesen K, Klerings I, Jørgensen KJ, M. K. Hand Cleaning with Ash for Reducing the Spread of Viral and Bacterial Infections: A Rapid Review. *Cochrane Database Syst. Rev.* **2020**, No. 4. https://doi.org/10.1002/14651858.CD013597.www.cochranelibrary.com.

(50) Amin, N.; Pickering, A. J.; Ram, P. K.; Unicomb, L.; Najnin, N.; Homaira, N.; Ashraf, S.; Abedin, J.; Islam, M. S.; Luby, S. P. Microbiological Evaluation of the Efficacy of Soapy Water to Clean

For peer review only

Hands: A Randomized, Non-Inferiority Field Trial. *Am. J. Trop. Med. Hyg.* **2014**, *91* (2), 415–423. https://doi.org/10.4269/ajtmh.13-0475.
(51) World Health Organization Western Pacific Region. *Considerations for Community Hand Hygiene Practices in Low- Resource Situations*; 2020.
(52) United Nations Children's Fund (UNICEF); World Health Organization (WHO). *State of the World's Hand Hygiene: A Global Call to Action to Make Hand Hygiene a Priority in Policy and Practice*; UNICEF: New York, New York, 2021.
(53) White, S.; Thorseth, A. H.; Dreibelbis, R.; Curtis, V. The Determinants of Handwashing Behaviour in Domestic Settings: An Integrative Systematic Review. *Int. J. Hyg. Environ. Health* **2020**, *227*, 113512. https://doi.org/10.1016/J.IJHEH.2020.113512.
(54) Michie, S.; Stralen, M. van; West, R. The Behaviour Change Wheel: A New Method for Characterising and Designing Behaviour Change Interventions. *Implement. Sci.* **2011**, *6* (42). https://doi.org/10.1001/archderm.1985.01660070119033.
(55) Aunger, R.; Curtis, V. Behaviour Centred Design: Towards an Applied Science of Behaviour Change. *Health Psychol. Rev.* **2016**, *10* (4), 425–446. https://doi.org/10.1080/17437199.2016.1219673.
(56) West, R.; Michie, S. A Brief Introduction to the COM-B Model of Behaviour and the PRIME Theory of Motivation. Qeios 2020.
(57) Curtis, V.; Kanki, B.; Cousens, S.; Sanou, A.; Diallo, I.; Mertens, T. Dirt and Diarrhoea Formative Research in Hygiene Promotion Programmes. *Health Policy Plan.* **1997**, *12* (2), 122–131.

For peer review only

Figure legends

Figure 1. Conceptual framework for hand hygiene in community settings.
Figure 2. PRISMA flow diagram.
Figure 3. Percentage of recommendations not supported by evidence and by type of evidence.

Exclusive license

I, the Submitting Author has the right to grant and does grant on behalf of all authors of the Work (as defined in the below author licence), an exclusive licence and/or a non-exclusive licence for contributions from authors who are: i) UK Crown employees; ii) where BMJ has agreed a CC-BY licence shall apply, and/or iii) in accordance with the terms applicable for US Federal Government officers or employees acting as part of their official duties; on a worldwide, perpetual, irrevocable, royalty-free basis to BMJ Publishing Group Ltd ("BMJ") its licensees and where the relevant Journal is co-owned by BMJ to the co-owners of the Journal, to publish the Work in BMJ Global Health and any other BMJ products and to exploit all rights, as set out in our licence.

The Submitting Author accepts and understands that any supply made under these terms is made by BMJ to the Submitting Author unless you are acting as an employee on behalf of your employer or a postgraduate student of an affiliated institution which is paying any applicable article publishing charge ("APC") for Open Access articles. Where the Submitting Author wishes to make the Work available on an Open Access basis (and intends to pay the relevant APC), the terms of reuse of such Open Access shall be governed by a Creative Commons licence – details of these licences and which Creative Commons licence will apply to this Work are set out in our licence referred to above.

For peer review only

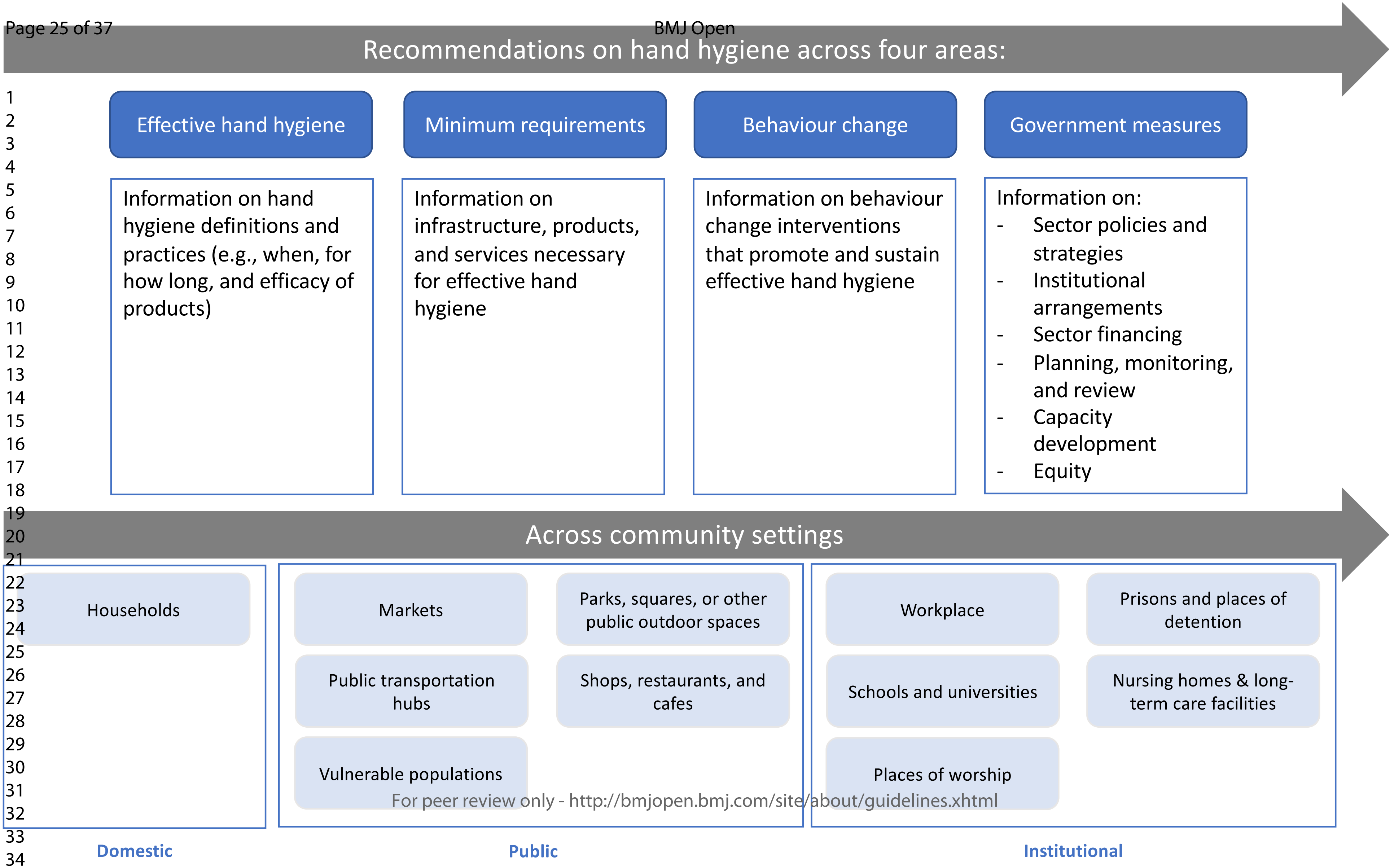
Recommendations on hand hygiene across four areas:
Effective hand hygiene
Minimum requirements
Behaviour change
Government measures
Information on hand hygiene definitions and practices (e.g., when, for how long, and efficacy of products)
Information on infrastructure, products, and services necessary for effective hand hygiene
Information on behaviour change interventions that promote and sustain effective hand hygiene
Information on:
- Sector policies and strategies
- Institutional arrangements
- Sector financing
- Planning, monitoring, and review
- Capacity development
- Equity
Across community settings
Households
Markets
Parks, squares, or other public outdoor spaces
Public transportation hubs
Shops, restaurants, and cafes
Vulnerable populations
Workplace
Prisons and places of detention
Schools and universities
Nursing homes & long-term care facilities
Places of worship
Domestic
Public
Institutional

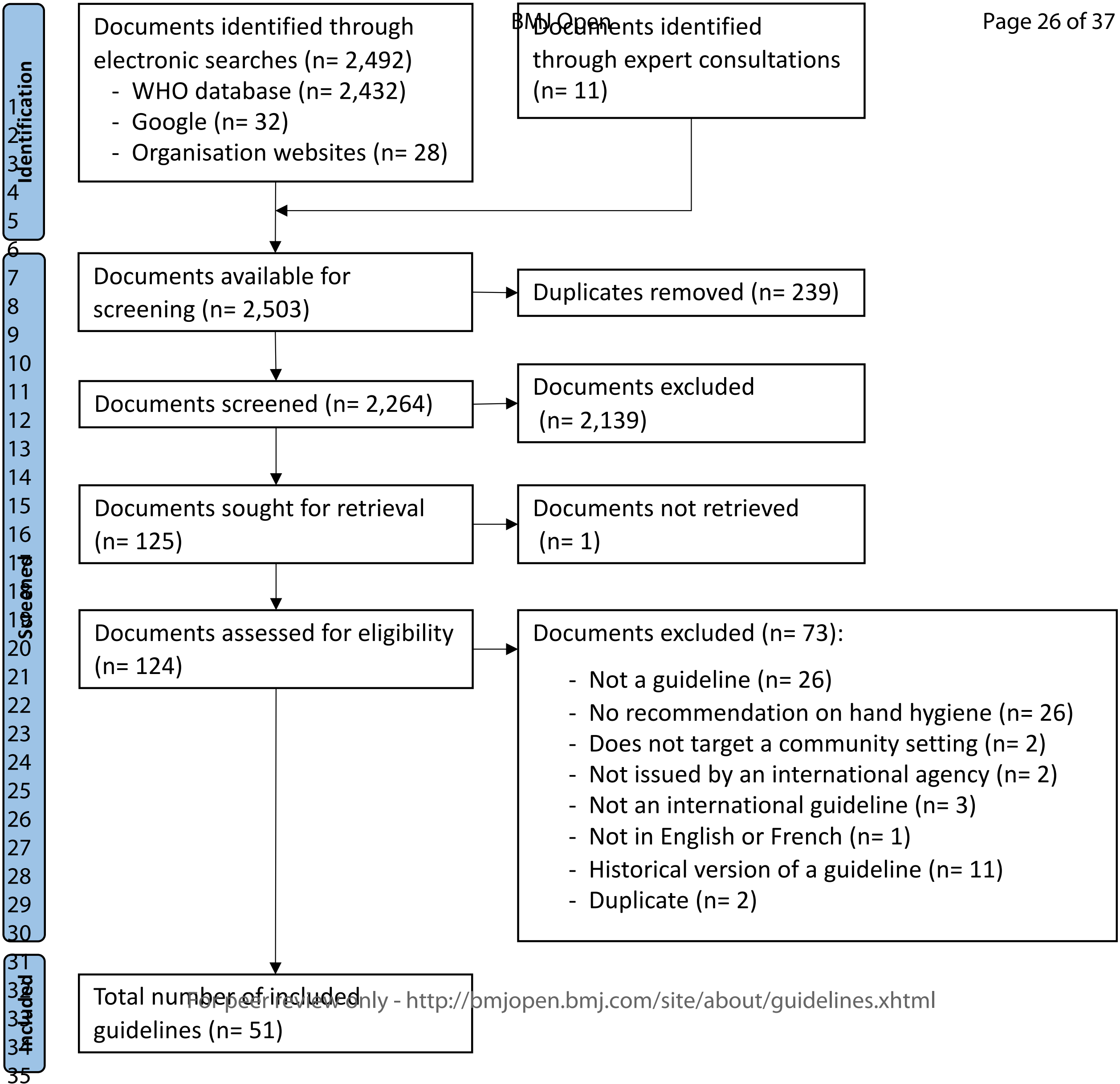
Identification
Documents identified through electronic searches (n= 2,492)
- WHO database (n= 2,432)
- Google (n= 32)
- Organisation websites (n= 28)
Documents identified through expert consultations (n= 11)
Screened
Documents available for screening (n= 2,503)
Duplicates removed (n= 239)
Documents screened (n= 2,264)
Documents excluded (n= 2,139)
Documents sought for retrieval (n= 125)
Documents not retrieved (n= 1)
Documents assessed for eligibility (n= 124)
Documents excluded (n= 73):
- Not a guideline (n= 26)
- No recommendation on hand hygiene (n= 26)
- Does not target a community setting (n= 2)
- Not issued by an international agency (n= 2)
- Not an international guideline (n= 3)
- Not in English or French (n= 1)
- Historical version of a guideline (n= 11)
- Duplicate (n= 2)
Included
Total number of included guidelines (n= 51)

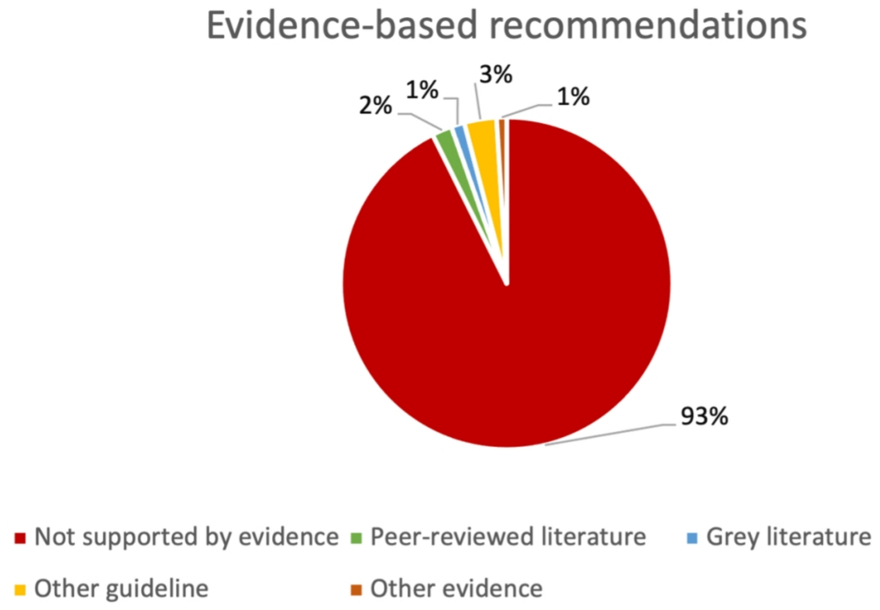

Percentage of recommendations not supported by evidence and by type of evidence.

114x71mm (300 x 300 DPI)

SUPPLEMENTAL MATERIALS

Table A1. The completed Preferred Reporting Items for Systematic reviews and Meta-Analyses extension for Scoping Reviews (PRISMA-ScR) checklist.[25]

SECTION	ITEM	PRISMA-ScR CHECKLIST ITEM	REPORTED ON PAGE #
**TITLE**			
Title	1	Identify the report as a scoping review.	2
**ABSTRACT**			
Structured summary	2	Provide a structured summary that includes (as applicable): background, objectives, eligibility criteria, sources of evidence, charting methods, results, and conclusions that relate to the review questions and objectives.	3
**INTRODUCTION**			
Rationale	3	Describe the rationale for the review in the context of what is already known. Explain why the review questions/objectives lend themselves to a scoping review approach.	5
Objectives	4	Provide an explicit statement of the questions and objectives being addressed with reference to their key elements (e.g., population or participants, concepts, and context) or other relevant key elements used to conceptualize the review questions and/or objectives.	5
**METHODS**			
Protocol and registration	5	Indicate whether a review protocol exists; state if and where it can be accessed (e.g., a Web address); and if available, provide registration information, including the registration number.	5
Eligibility criteria	6	Specify characteristics of the sources of evidence used as eligibility criteria (e.g., years considered, language, and publication status), and provide a rationale.	6
Information sources	7	Describe all information sources in the search (e.g., databases with dates of coverage and contact with authors to identify additional sources), as well as the date the most recent search was executed.	6
Search	8	Present the full electronic search strategy for at least 1 database, including any limits used, such that it could be repeated.	Supplemental material Table A5
Selection of sources of evidence	9	State the process for selecting sources of evidence (i.e., screening and eligibility) included in the scoping review.	6
Data charting process	10	Describe the methods of charting data from the included sources of evidence (e.g., calibrated forms or forms that have been tested by the team before their use, and whether data charting was done independently or in duplicate) and any processes for obtaining and confirming data from investigators.	6
Data items	11	List and define all variables for which data were sought and any assumptions and simplifications made.	6
Critical appraisal of individual sources of evidence	12	If done, provide a rationale for conducting a critical appraisal of included sources of evidence; describe the methods used and how this information was used in any data synthesis (if appropriate).	6 – 7

For peer review only

SECTION	ITEM	PRISMA-ScR CHECKLIST ITEM	REPORTED ON PAGE #
Synthesis of results	13	Describe the methods of handling and summarizing the data that were charted.	6 – 7
**RESULTS**			
Selection of sources of evidence	14	Give numbers of sources of evidence screened, assessed for eligibility, and included in the review, with reasons for exclusions at each stage, ideally using a flow diagram.	7
Characteristics of sources of evidence	15	For each source of evidence, present characteristics for which data were charted and provide the citations.	Supplemental material Table A6
Critical appraisal within sources of evidence	16	If done, present data on critical appraisal of included sources of evidence (see item 12).	14
Results of individual sources of evidence	17	For each included source of evidence, present the relevant data that were charted that relate to the review questions and objectives.	7 – 14
Synthesis of results	18	Summarize and/or present the charting results as they relate to the review questions and objectives.	7 – 14
**DISCUSSION**			
Summary of evidence	19	Summarize the main results (including an overview of concepts, themes, and types of evidence available), link to the review questions and objectives, and consider the relevance to key groups.	14 – 16
Limitations	20	Discuss the limitations of the scoping review process.	16
Conclusions	21	Provide a general interpretation of the results with respect to the review questions and objectives, as well as potential implications and/or next steps.	6
**FUNDING**			
Funding	22	Describe sources of funding for the included sources of evidence, as well as sources of funding for the scoping review. Describe the role of the funders of the scoping review.	17

Table A2. List of websites searched.

Agency or resource hub	Website
Africa Centers for Disease Control and Prevention (CDC)	https://africacdc.org/
Global Handwashing Partnership	https://globalhandwashing.org/
Global WASH Cluster	www.washcluster.org
International Red Cross and Red Crescent (ICRC)	www.icrc.org
International Federation of the Red Cross (IFRC)	www.ifrc.org
International Labour Organization (ILO)	https://www.ilo.org
International Centre for Diarrhoeal Disease Research Bangladesh (ICDDR'B)	www.icddrb.org
Oxfam	www.oxfam.org.uk

For peer review only

Sanitation Learning Hub	https://sanitationlearninghub.org/
Save the Children	www.nrc.no
SNV Netherlands Development Organisation	https://snv.org/
Sustainable Sanitation Alliance	https://www.susana.org/en/
United Nations Human Settlement Programme (UN Habitat)	https://unhabitat.org/
United Nations High Commissioner for Refugees (UNHCR)	www.unhcr.org
United Nations Children's Fund (UNICEF)	www.unicef.org
US Centers for Disease Control and Prevention (CDC)	www.cdc.gov
WASH'Em	https://www.washem.info/
WaterAid	https://www.wateraid.org/us/
World Bank	https://www.worldbank.org/en/home
Water, Education and Development Centre (WEDC)	www.wedc.lboro.ac.uk
Water and Sanitation for the Urban Poor (WSUP)	https://www.wsup.com/
Deutsche Gesellschaft für Internationale Zusammenarbeit (GIZ)	https://www.giz.de
United States Agency for International Development (USAID)	https://www.usaid.gov/

Table A3. Search terms.

Concept	Search terms
Hand hygiene	Handwash* OR hygiene OR hand wash* OR with soap OR hand hygiene* OR clean hands OR WASH
Community settings	community OR household OR domestic OR public space OR institution* OR work* OR occupation* OR school OR prison OR market* OR relig* OR education* OR detention*
Guideline	guideline OR guide OR handbook OR manual OR toolkit

Table A4. Hand Hygiene for All Initiative core partners.

United Nations Children's Fund
International Labour Organization
World Bank
United Nations High Commission for Refugees
COVID-19 Hygiene Hub
Sanitation and Water for All
Global Handwashing Partnership
International Federation of Red Cross
WaterAid

For peer review only

Table A5. Excluded documents with reasons.

Author	Guideline title	Year	Reason for exclusion
ICRC	Water, sanitation, and hygiene, and habitat in prisons	2005	Historical version of guideline
World Bank	The Handwashing Handbook: a guide for developing a hygiene promotion program to increase handwashing with soap	2005	Historical version of guideline
IRC	Strengthening Water, Sanitation and Hygiene in schools: a WASH guidance manual with a focus on South Asia	2010	Not a guideline
ICRC	Water, sanitation, and hygiene, and habitat in prisons: supplementary guidance	2012	No recommendation on hand hygiene
GIZ	Field guide: hardware for handwashing in schools	2013	Not a guideline
WEDC	Guidelines for handwashing with soap	2013	Not issued by an international agency
NRC	Water, sanitation and hygiene manual: WASH training for hygiene promotion staff	2015	Not a guideline
ILO	WASH@Work: self-training handbook	2016	Historical version of guideline
WSUP	School hygiene manual	2016	Not for international audience
Rotary	A guide to WASH in schools	2017	Not a guideline
Global Handwashing Partnership	Clean hands for all: a toolkit for hygiene advocacy	2018	Not a guideline
COOPI	WASH handbook for teachers and facilitators	2019	Not for international audience
WASH’Em	How to design handwashing facilities that change behaviour	2019	Does not target a community setting
Africa CDC	Hand washing facility options for resource limited settings	2020	Not a guideline
British Psychological Society	Behavioural science and disease prevention psychological guidance: encouraging hand hygiene in the community	2020	Not a guideline
Coultas et al.	Handwashing compendium for low-resource settings: a living document, 3rd edition	2020	Not a guideline
Global Handwashing Partnership	The Handwashing Handbook	2020	Not a guideline
Smart Centre Group	Smart hygiene solutions: affordable options for household level	2020	Not a guideline

UN Habitat	UN-Habitat guidance on COVID-19 and public spaces	2020	No recommendation on hand hygiene
UNHCR	COVID-19 refugees return to schooling guidelines	2020	No recommendation on hand hygiene
UNICEF et al.	Panduan Cuci Tangan Pakai Sabun	2020	Not in English or French
WaterAid	COVID-19 guidance: prioritising hygiene for workforce health and business resilience	2020	No recommendation on hand hygiene
WaterAid Nepal	Technical brief on contactless handwashing stations	2020	Not a guideline
Bolton, L.	WASH in schools for student return during the COVID-19 pandemic	2021	Not issued by an international agency
ILO	WASH@Work: self-training handbook (revised)	2021	Not a guideline
Government of Vietnam	A guide to integrated hand washing with soap communication	n/d	Not for international audience
WaterAid	Technical manual on community water supply, hygiene and sanitation facilities	n/d	Not a guideline
WaterAid Bangladesh	Handwashing stations: an easy-to-use technological and context-based handwashing stations manual	n/d	Not a guideline

Table A6. List of included guidelines.

Author	Guideline	Year	Community setting	COVID-19 response
UNICEF	A manual on hygiene promotion	1999	Public	No
WHO	WHO recommended strategies for the prevention and control of communicable diseases	2001	Domestic & public	No
WHO	Health villages: a guide for communities and community health workers	2002	Public	No
WHO	Water, sanitation, and hygiene standards for schools in low-cost settings	2009	Institutional	No
ICRC	Water, sanitation, hygiene, and habitat in prisons	2013	Institutional	No
ICRC	Health care in detention	2015	Institutional	No
WHO	Public health for mass gatherings: key considerations	2015	Public	No
SNV	Behaviour change communication guidelines	2016	Public	No
UNICEF, GIZ	Scaling up group handwashing in schools: compendium of group washing facilities across the globe	2016	Schools	No
Malteser	WASH guidelines for field practitioners	2017	Multiple settings	No
Oxfam	Handwashing technical briefing note	2017	Public	No

UNHCR	UNHCR hygiene promotion guidelines	2017	Public	No
WHO	Guidelines on sanitation and health	2018	Domestic & public	No
WHO	Recommendations to Member States to improve hand hygiene practices to help prevent COVID-19	2020	Public	Yes
UNICEF	Handwashing stations and supplies for the COVID-19 response	2020	Domestic & public	Yes
ILO	Hand hygiene at the workplace: an essential occupational safety and health prevention and control measure against COVID-19	2020	Workplace	Yes
GOAL	WASH and IPC measures in households and public spaces	2020	Domestic & public	No
WHO	Considerations for community hand hygiene practices in low-resource situations	2020	Public	No
WHO	Overview of public health and social measures in the context of COVID-19	2020	Public	Yes
GWC	COVID-19 response guidance note - 15 April 2020	2020	Domestic & public	Yes
GWC	Health and hygiene promotion guidance document for COVID-19	2020	Public	Yes
ILO	Safe return to work: guide for employers on COVID-19 prevention	2020	Institutional	Yes
SNV	Practical options for hand-washing stations: a guide for promoters and producers	2020	Multiple settings	No
UNHCR	Technical WASH guidance for COVID-19 preparedness and response	2020	Public	Yes
UNICEF	COVID-19 emergency preparedness and response: WASH and infection prevention and control measures in schools	2020	Schools	Yes
UNICEF	COVID-10 handwashing with soap (HWWS) facilities: compendium of indicative layouts, designs, and cost estimates	2020	Public	Yes
UNICEF et al.	Handwashing with soap in schools: a technical options manual	2020	Schools	No
UNICEF, WHO, IFRC	Key messages and actions for COVID-19 prevention and control in schools	2020	Schools	Yes
UNICEF	Understanding Hygiene promotion in the context of Risk Communication & Community Engagement (RCCE) and Infection Control and Prevention (IPC) for the COVID-19 outbreak	2020	Domestic & public	Yes
UNICEF	WASH and Infection Prevention and Control (IPC) measures in households and public spaces	2020	Domestic & public	Yes
UNICEF, WHO, IFRC	Interim guidance for COVID-19 prevention and control in schools	2020	Institutional	Yes
WASH4WORK	Hand hygiene protocol for the workplace	2020	Institutional	Yes
Water Mission	Handwashing station program: requirements and recommendations	2020	Public	Yes
WaterAid	Technical guide for handwashing facilities in public spaces and buildings	2020	Public	Yes
WHO	Actions for consideration in the case and protection of vulnerable populations from COVID-19	2020	Vulnerable populations	Yes

For peer review only - http://bmjopen.bmj.com/site/about/guidelines.xhtml

WHO	Considerations for public health and social measures in the workplace in the context of COVID-19: Annex to Considerations in adjusting public health and social measures in the context of COVID-19	2020	Workplace	Yes
WHO	Considerations for school-related public health measures in the context of COVID-19: Annex to Considerations in adjusting public health and social measures in the context of COVID-19	2020	Institutional	Yes
WHO	Guidance on COVID-19 for the care of older people and people living in long-term care facilities, other non- acute care facilities and home care	2020	Institutional	Yes
WHO	Interim recommendations on obligatory hand hygiene against transmission of COVID-19	2020	Public spaces	Yes
WHO	Practical considerations and recommendations for religious leaders and faith-based communities in the context of COVID-19	2020	Public spaces	Yes
WHO	Water, sanitation, hygiene, and waste management for SARS-CoV-2, the virus that causes COVID-19: interim guidance	2020	Domestic & public	Yes
CDC	When and how to wash your hands	2021	Public	No
CDC	Hand hygiene at school	2021	Institutional	No
CDC	Hand hygiene at work	2021	Institutional	No
CDC	Interim guidance on management of coronavirus disease 2019 (COVID-19) in correctional and detention facilities	2021	Institutional	Yes
CDC	Interim guidance on people experiencing unsheltered homelessness	2021	Vulnerable populations	Yes
WHO	Key planning recommendations for mass gatherings in the context of COVID-19: interim guidance	2021	Public	Yes
WHO	Preparedness, prevention, and control of COVID-19 in prisons and other places of detention: interim guidance	2021	Institutional	Yes
WHO, ILO	Preventing and mitigating COVID-19 at work	2021	Institutional	Yes
WHO	Safe Eid al Adha practices in the context of COVID-19: interim guidance	2021	Public	Yes
WHO	Safe Ramadan practices in the context of COVID-19: interim guidance	2021	Public	Yes

For peer review only - http://bmjopen.bmj.com/site/about/guidelines.xhtml

Table A7. Number of recommendations per parameter across all community settings.

Parameters	Number of recommendations	Number of guidelines with recommendations
**Safe hand hygiene**		
Guidelines that define hand hygiene	3	3
Guidelines that define safe hand hygiene	2	2
Critical control points language	38	38
Critical control points	145	38
Basic handwashing knowledge	8	8
Basic hand hygiene knowledge	0	0
**Handwashing with soap & hand drying**		
Handwashing infrastructure	33	24
Handwashing materials	68	46
Amount of soap	7	6
Handwashing services	37	30
Handwashing with soap duration	17	17
Water quality	19	16
Water quantity	9	10
Alternative water sources for handwashing	15	16
Greywater management	23	13
Location of handwashing stations	59	30
Spacing and number of users per handwashing station	9	8
Hand drying materials	36	19
Disposal of hand drying materials	9	9
Conditional recommendations	7	7
**Handwashing with soap alternatives**		
Alcohol-based hand rub	32	32
Hand rubbing duration	3	3
Other materials for hand hygiene	39	17
Conditional recommendations	29	22
Alternatives not recommended	11	7
Location of hand hygiene stations	21	12
**Hand hygiene station design considerations**		
Design principles	10	6
Technical considerations	5	4
Accessibility	19	14
Local procurement and production	11	9
Supply chain	3	3
Cost and affordability	7	7
Durability	8	6
COVID-19 adaptations	26	16
Other adaptations	17	10
Operation and maintenance – responsibility	8	6
Operation and maintenance – actions	9	8
**Changing behaviour**		
Behaviour change techniques	29	14
Content of behaviour change messaging	27	25
Behaviour change delivery channels	42	25
Person or group tasked with delivery behaviour change intervention	13	10
Frequency of behaviour change interventions	2	2
Formative research	5	4

For peer review only

Behaviour change models or frameworks	15	3
Behaviour change approaches	9	2
Hand hygiene determinants	11	3
**Government interventions**		
Strengthening markets for hand hygiene products and services	2	2
Hygiene promotion practices	0	0
At-scale behaviour change interventions	0	0
Sector policy and strategy	0	0
Institutional arrangements	5	5
Sector financing	0	0
Planning, monitoring, and review	2	2
Capacity development	1	1
Equity	1	1

For peer review only

Table A1. The completed Preferred Reporting Items for Systematic reviews and Meta-Analyses extension for Scoping Reviews (PRISMA-ScR) checklist.

SECTION	ITEM	PRISMA-ScR CHECKLIST ITEM	REPORTED ON PAGE #
**TITLE**			
Title	1	Identify the report as a scoping review.	2
**ABSTRACT**			
Structured summary	2	Provide a structured summary that includes (as applicable): background, objectives, eligibility criteria, sources of evidence, charting methods, results, and conclusions that relate to the review questions and objectives.	3
**INTRODUCTION**			
Rationale	3	Describe the rationale for the review in the context of what is already known. Explain why the review questions/objectives lend themselves to a scoping review approach.	5
Objectives	4	Provide an explicit statement of the questions and objectives being addressed with reference to their key elements (e.g., population or participants, concepts, and context) or other relevant key elements used to conceptualize the review questions and/or objectives.	5
**METHODS**			
Protocol and registration	5	Indicate whether a review protocol exists; state if and where it can be accessed (e.g., a Web address); and if available, provide registration information, including the registration number.	5
Eligibility criteria	6	Specify characteristics of the sources of evidence used as eligibility criteria (e.g., years considered, language, and publication status), and provide a rationale.	6
Information sources	7	Describe all information sources in the search (e.g., databases with dates of coverage and contact with authors to identify additional sources), as well as the date the most recent search was executed.	6
Search	8	Present the full electronic search strategy for at least 1 database, including any limits used, such that it could be repeated.	Supplemental material Table A5
Selection of sources of evidence	9	State the process for selecting sources of evidence (i.e., screening and eligibility) included in the scoping review.	6
Data charting process	10	Describe the methods of charting data from the included sources of evidence (e.g., calibrated forms or forms that have been tested by the team before their use, and whether data charting was done independently or in duplicate) and any processes for obtaining and confirming data from investigators.	6
Data items	11	List and define all variables for which data were sought and any assumptions and simplifications made.	6
Critical appraisal of individual sources of evidence	12	If done, provide a rationale for conducting a critical appraisal of included sources of evidence; describe the methods used and how this information was used in any data synthesis (if appropriate).	6 – 7
Synthesis of results	13	Describe the methods of handling and summarizing the data that were charted.	6 – 7

For peer review only

SECTION	ITEM	PRISMA-ScR CHECKLIST ITEM	REPORTED ON PAGE #
**RESULTS**			
Selection of sources of evidence	14	Give numbers of sources of evidence screened, assessed for eligibility, and included in the review, with reasons for exclusions at each stage, ideally using a flow diagram.	7
Characteristics of sources of evidence	15	For each source of evidence, present characteristics for which data were charted and provide the citations.	Supplemental material Table A6
Critical appraisal within sources of evidence	16	If done, present data on critical appraisal of included sources of evidence (see item 12).	14
Results of individual sources of evidence	17	For each included source of evidence, present the relevant data that were charted that relate to the review questions and objectives.	7 – 14
Synthesis of results	18	Summarize and/or present the charting results as they relate to the review questions and objectives.	7 – 14
**DISCUSSION**			
Summary of evidence	19	Summarize the main results (including an overview of concepts, themes, and types of evidence available), link to the review questions and objectives, and consider the relevance to key groups.	14 – 16
Limitations	20	Discuss the limitations of the scoping review process.	16
Conclusions	21	Provide a general interpretation of the results with respect to the review questions and objectives, as well as potential implications and/or next steps.	6
**FUNDING**			
Funding	22	Describe sources of funding for the included sources of evidence, as well as sources of funding for the scoping review. Describe the role of the funders of the scoping review.	17

For peer review only

BMJ Open

Recommendations for hand hygiene in community settings: a scoping review of current international guidelines

Journal:	*BMJ Open*
Manuscript ID	bmjopen-2022-068887.R1
Article Type:	Original research
Date Submitted by the Author:	07-Dec-2022
Complete List of Authors:	MacLeod, Clara; London School of Hygiene and Tropical Medicine Faculty of Infectious and Tropical Diseases, Department of Disease Control<br>Braun, Laura; London School of Hygiene and Tropical Medicine Faculty of Infectious and Tropical Diseases, Department of Disease Control<br>Caruso, Bethany; Emory University, Hubert Department of Global Health, Rollins School of Public Health<br>Chase, Claire; World Bank Group<br>Chidziwisano, Kondwani; Malawi University of Business and Applied Sciences, Department of Environmental Health and WASHTED<br>Chipungu, Jenala; Center for Infectious Disease Research in Zambia<br>Dreibelbis , Robert; London School of Hygiene and Tropical Medicine Faculty of Infectious and Tropical Diseases, Department of Disease Control<br>Ejemot-Nwadiaro, Regina; University of Calabar, Department of Public Health, College of Medical Sciences<br>Gordon, Bruce ; World Health Organization, Water, Sanitation, Hygiene and Health Unit<br>Esteves Mills, Joanna; World Health Organization, Water, Sanitation, Hygiene and Health Unit<br>Cumming, Oliver; London School of Hygiene and Tropical Medicine Faculty of Infectious and Tropical Diseases, Department of Disease Control
**Primary Subject Heading**:	Public health
Secondary Subject Heading:	Global health, Public health
Keywords:	Public health < INFECTIOUS DISEASES, PUBLIC HEALTH, COVID-19

SCHOLARONE™
Manuscripts

I, the Submitting Author has the right to grant and does grant on behalf of all authors of the Work (as defined in the below author licence), an exclusive licence and/or a non-exclusive licence for contributions from authors who are: i) UK Crown employees; ii) where BMJ has agreed a CC-BY licence shall apply, and/or iii) in accordance with the terms applicable for US Federal Government officers or employees acting as part of their official duties; on a worldwide, perpetual, irrevocable, royalty-free basis to BMJ Publishing Group Ltd ("BMJ") its licensees and where the relevant Journal is co-owned by BMJ to the co-owners of the Journal, to publish the Work in this journal and any other BMJ products and to exploit all rights, as set out in our licence.

The Submitting Author accepts and understands that any supply made under these terms is made by BMJ to the Submitting Author unless you are acting as an employee on behalf of your employer or a postgraduate student of an affiliated institution which is paying any applicable article publishing charge ("APC") for Open Access articles. Where the Submitting Author wishes to make the Work available on an Open Access basis (and intends to pay the relevant APC), the terms of reuse of such Open Access shall be governed by a Creative Commons licence – details of these licences and which Creative Commons licence will apply to this Work are set out in our licence referred to above.

Other than as permitted in any relevant BMJ Author's Self Archiving Policies, I confirm this Work has not been accepted for publication elsewhere, is not being considered for publication elsewhere and does not duplicate material already published. I confirm all authors consent to publication of this Work and authorise the granting of this licence.

For peer review only

Title

Recommendations for hand hygiene in community settings: a scoping review of current international guidelines

Authors

Clara MacLeod[1,*], Laura Braun[1], Bethany A. Caruso[2], Claire Chase[3], Kondwani Chidziwisano[4], Jenala Chipungu[5], Robert Dreibelbis[1], Regina Ejemot-Nwadiaro[6], Bruce Gordon[7], Joanna Esteves Mills[7], Oliver Cumming[1]

Affiliations

[1] Department of Disease Control, London School of Hygiene and Tropical Medicine, Keppel St, London, WC1E 7HT, UK
[2] Hubert Department of Global Health, Rollins School of Public Health, Emory University, Atlanta, Georgia
[3] The World Bank, Washington, DC 20433, United States of America
[4] Department of Environmental Health and WASHTED, Malawi University of Business and Applied Sciences, Blantyre, Malawi
[5] Centre for Infectious Disease Research in Zambia, Lusaka, Zambia
[6] Department of Public Health, College of Medical Sciences, University of Calabar, Calabar, Nigeria
[7] Water, Sanitation, Hygiene and Health Unit, World Health Organization, Avenue Appia 20, 1202 Geneva, Switzerland

* Correspondence to Clara MacLeod: clara.macleod@lshtm.ac.uk

Word count: 5,298

Abstract count: 276

Number of references: 98

For peer review only

ABSTRACT

Objective: To summarise current recommendations for hand hygiene in community settings among international guidelines and assess to what extent they are consistent and evidence-based.

Methods: To identify international guidelines with recommendations on hand hygiene in community settings, categorised as either domestic, public, and institutional, published between 1 January 1990 and 15 November 2021, we performed electronic and grey literature searches and contacted expert organisations and individuals. Recommendations extracted from included guidelines were mapped to four areas related to hand hygiene: i) effective hand hygiene; ii) minimum requirements; iii) behaviour change; and iv) government measures. We assessed if recommendations were supported by various levels of evidence and checked their consistency and concordance across community settings.

Results: We identified 51 guidelines published between 1999 and 2021 by multilateral agencies and international non-governmental organisations containing 923 recommendations. Handwashing with soap is consistently recommended as the preferred method for hand hygiene across all community settings. Most guidelines specifically recommend handwashing with plain soap and running water for at least 20 seconds; single-use paper towels for hand drying; and alcohol-based hand rub (ABHR) as a complement or alternative to handwashing. There are inconsistent and discordant recommendations for water quality for handwashing, affordable and effective alternatives to soap and ABHR, and the design of handwashing stations. Further, there are gaps in recommendations on soap and water quantity, behaviour change approaches, and government measures required for effective hand hygiene. Overall, less than 10% of recommendations were supported by any cited evidence.

Conclusion: While current international guidelines consistently recommend handwashing with soap in domestic, public, and institutional settings, there remain gaps in recommendations where clear evidence-based guidance might support more effective policy and investment.

For peer review only

Strengths and limitations of this study

- This is the first scoping review to synthesise current recommendations for hand hygiene in community settings among international guidelines and assess to what extent they are consistent and evidence-based.
- We identified 51 guidelines with over 900 recommendations for hand hygiene in community settings published by multilateral agencies and international non-governmental organisations.
- The search was limited to guidelines published in English or French and therefore may have excluded relevant guidelines published in other languages.
- The quality of the included guidelines was not assessed, although we did consider the extent to which recommendations were based on cited evidence.

For peer review only

INTRODUCTION

Hand hygiene, including handwashing with soap and other methods such as alcohol-based hand rubs (ABHRs), is an important public health measure that can prevent the transmission of a range of diseases.[1] Handwashing with soap has been found to be a cost-effective intervention[2] that can reduce the risk of both diarrhoeal disease and acute respiratory infections (ARIs) by over 20%.[3–9] Handwashing with soap has also been linked to the reduction of certain neglected tropical diseases (NTDs), including trachoma and some soil-transmitted helminth infections.[10,11] Recently, handwashing with soap and the use of ABHRs were advised as one of the key control measures during the COVID-19 pandemic[12,13] and were found to be effective.[14]

This scoping review focuses on hand hygiene in non-healthcare settings, which we collectively refer to as "community settings". Using the definition set out in the Ottawa Charter, we consider settings as where "health is created and lived by people within the settings of their everyday life; where they learn, work, play and love",[15] and include domestic, public, and institutional settings. The practice of hand hygiene – and access to the facilities which enable this – is often limited in these community settings, particularly in low- and middle-income countries.[16] In the domestic setting, 30% of the global population does not have access to a basic handwashing facility with soap and water at home,[17,18] with three quarters of those living in low-income countries.[17] In institutional settings, an estimated 43% of schools worldwide do not have access to basic hand hygiene facilities[18] but there is limited data for other institutional settings, such as the workplace and prisons and places of detention, and public settings, such as markets, transportation hubs, and places of worship.[18]

Despite the international recognition of hand hygiene as a critical public health measure, a recent global assessment of government policies, planning, and financing for hygiene found that while the majority of surveyed countries reported having national policies for hand hygiene, less than 10% had sufficient financing to implement them.[19] Various international guidelines with recommendations on hand hygiene for non-healthcare settings exist[13,20–22] but it is unclear whether current international guidelines are comprehensive, consistent, and based on the most rigorous evidence available. This review aims to summarise current international recommendations for hand hygiene in community settings, identify areas of consistency and concordance, and assess whether recommendations are evidence-based.

METHODS

This review follows the six stages of the Arksey and O'Malley methodological framework for scoping reviews.[23–25] Expert consultation (stage 6) was integrated throughout the scoping review process[24] to obtain feedback on the scope and conceptual framework and to identify any additional guidelines beyond those identified during the electronic search. Our review is described according to the Preferred Reporting Items for Systematic reviews and Meta-Analyses extension for Scoping Reviews (PRISMA-ScR)[26] and a PRISMA-ScR checklist is included in the supplementary materials (Table A1). The protocol was pre-registered with OSF Registries.[27]

Identifying the research question (stage 1)

To identify and refine the research question, a conceptual framework was developed for this review and built around three key concepts: i) hand hygiene, ii) non-healthcare settings, and iii) current international guidelines (Figure 1). In this review, we define hand hygiene as any action of hand cleansing for the purpose of removing or deactivating pathogens from hands.[28] Effective hand hygiene is defined as any practice which removes or deactivates pathogens from hands and thereby limits disease transmission.[28]

The first key concept in the conceptual framework – hand hygiene – covers four areas: effective hand hygiene, minimum requirements, behaviour change, and government measures (Figure 1). Effective hand hygiene refers to definitions and practices. Minimum requirements refers to the materials, services, and infrastructure required for effective hand hygiene. Behaviour change denotes the appropriate behavioural performances that promote and sustain effective hand hygiene. Government measures concern actions taken by governments to ensure effective hand hygiene, which are categorised according to an established framework[29] as follows: policy and strategy; institutional arrangements; sector financing; planning, monitoring, and review; capacity development; and equity.

The second key concept – community settings – is defined as settings where healthcare is not routinely delivered,[28] broadly spanning all places where people "learn, play, work and love"[15] and specifically including domestic, public, and institutional settings. As these settings may not exclusively refer to physical settings,[30] the review also includes recommendations for vulnerable populations (e.g., people experiencing homelessness) who may reside permanently or semi-permanently in public spaces. Nursing homes, long-term care facilities, non-acute care facilities, and home care are also included in the review under institutional settings, as the boundary between healthcare and non-healthcare settings is often unclear or recommendations for these sub-settings may not be disaggregated, especially in the context of COVID-19 where people received care at home.

The third concept – guideline – is defined as a published document where the primary purpose is to provide specific guidance, in the form of recommendations, towards a course of action. A recommendation is defined as a statement designed to assist a targeted actor to make an informed decision on whether, when, and how to undertake a specific action.[31] Our review is limited to international guidelines to identify generalisable recommendations of global relevance.

Identifying relevant studies (stage 2)

To identify relevant guidelines, the search strategy consisted of the following sources: (1) the World Health Organization (WHO) Institutional Repository for Information Sharing (IRIS) database, (2) Google search engine, (3) websites of international organisations known to work on hand hygiene (Table A2), and (4) contacting experts. We searched the WHO IRIS database using pre-specified search terms related to hand hygiene, non-healthcare settings, and guidelines (Table A3). The search in Google was carried out using the anonymous function in the web browser (Chrome) to reduce the influence of the reviewer's (CM) individual search history. Search strings were constructed by using multiple combinations of search terms from Table A3. The first 10 pages of Google were reviewed by one reviewer (CM). Documents that at first appeared related to the research question and met the inclusion criteria were included for further screening. Expert consultations were also conducted with 'Hand Hygiene for All' Initiative core partners (Table A4) to identify potentially relevant guidelines. The reference lists of guidelines were also hand-searched for any additional relevant documents. The search was limited to English and French languages and publication date was restricted to 1 January 1990 onwards to identify current guidelines.

Study selection (stage 3)

Documents meeting the following criteria were included: i) international guideline, ii) offers one or more recommendations on hand hygiene, iii) targets at least one community setting, as defined in the conceptual framework, iv) published by an international non-governmental organisation (NGO), multilateral agency, or public health agency, v) published in English or French, and vi) published between 1 January 1990 and 15 November 2021. The review excludes guidelines for humanitarian settings, as internationally agreed guidance on hand hygiene in humanitarian settings and complex

For peer review only

emergencies is available through the Sphere standards for water, sanitation, and hygiene promotion.[32] Only the most recent versions of guidelines were included, with previous versions of the same guidelines excluded. Country-specific guidelines were also excluded.

All documents retrieved from electronic searches and expert consultations were transferred to Mendeley[33] for de-duplication. Inclusion was completed in two stages: (1) title and abstracts were screened for eligibility by one reviewer (CM); and (2) full texts of all potentially eligible documents were retrieved and independently assessed for inclusion by two reviewers (CM and LB). Disagreement between reviewers on inclusion was resolved through arbitration by a third reviewer (OC).

Charting the data (stage 4)

Guideline characteristics and recommendations from included guidelines were double-extracted by two reviewers (CM and LB) using a standardised data extraction template in MS Excel[34] and then cross-checked for accuracy. As with inclusion, a third reviewer (OC) provided arbitration if agreement on extraction could not be reached. The data extraction form (Table A5) included information on guideline characteristics, such as author, year of publication, target setting, and COVID-19 response, as well as 57 specific parameters related to the four areas of hand hygiene described in the conceptual framework (Figure 1). Recommendations for each parameter were extracted from included guidelines where possible.

Collating, summarising, and reporting the results (stage 5)

Guideline recommendations were first summarised for each parameter across community settings and then disaggregated by domestic, public, or institutional setting where relevant. Definitions and recommendations for hand hygiene were assessed for consistency, concordance, and whether supported by cited evidence. Recommendations were classified as consistent, fairly consistent, or inconsistent if they featured in 10 or more, four to nine, or less than four guidelines, respectively. Concordance is here defined as parameters with no consistent or fairly consistent recommendations at odds with each other. We also used a hierarchical system to classify evidence cited for recommendations into five levels, adapted according to an established classification system:[35] 1) systematic review or meta-analysis, 2) randomised controlled trial or controlled trial without randomisation (e.g., quasi-experimental), 3) guideline developed from systematic reviews, 4) observational study (e.g., cohort, cross-sectional, case-control studies), and 5) expert opinion or anecdotal information (e.g., programme documentation). A recommendation was considered evidence-based if the guideline provided a specific citation for the recommendation, which was coded according to the level of evidence cited. Finally, evidence gaps were defined as parameters with very few recommendations (i.e., less than 10 recommendations, equivalent to less than 20% of guidelines providing a recommendation).

Patient and public involvement

There was no public or patient involvement in the course of this project.

RESULTS

Search results

Electronic searches were conducted on 15 November 2021, identifying 2,492 records (2,432 from the WHO IRIS database, 32 from Google, 28 from international agency websites) (Table A2) and a further 11 records identified through expert consultation. Following de-duplication, a total of 2,264 records

were screened by title and abstract, and 125 documents were sought for retrieval for full-text screening, with one document not accessible. Finally, 51 guidelines are included in the review (Figure 2). The 73 documents excluded during the full-text review are listed in the Supplemental Material with reasons for exclusion (Table A6).

Description of included guidelines

The 51 included guidelines were published in English between 1999 and 2021, with 38 published in 2020 or later and 31 providing guidance specifically focusing on hand hygiene to help prevent the transmission of the COVID-19 virus. Among the 51 included guidelines, 67% are published by multilateral agencies (WHO, UNICEF, UNHCR, and ILO), 23% by international NGOs, and 10% by the United States Centers for Disease Control and Prevention (US CDC) (Table A7). Most guidelines target public and institutional settings, while none exclusively target the domestic setting. More specifically, 43% (n=22) target the public setting, 43% (n=22) the institutional setting, 4% (n=2) the domestic and public setting, and 10% (n=5) more than one setting (e.g., domestic, public, and/or institutional). Of the 22 guidelines for the public setting, 20 concern public spaces, and two concern vulnerable populations within public spaces (e.g., people experiencing unsheltered homelessness and people living in dense, informal settlements). For institutional settings, eight guidelines concern schools, six the workplace, four prisons and places of detention, three places of worship, and one long-term care facilities and home care. Almost no guidelines, however, define a community setting. The majority of recommendations are generalisable to high-, medium-, and low-income settings. Overall, we extracted 923 recommendations from the 51 included guidelines (Table A8).

Recommendations for effective hand hygiene

Hand hygiene definitions

Only 10% of guidelines provide a clear definition for hand hygiene or safe or effective hand hygiene.[36–40] Meanwhile, 75% of guidelines provide at least one recommendation on when to practice hand hygiene, referred to in our review as a 'key moment'. Guidelines use inconsistent terms for defining when to practice hand hygiene, such as 'key times' (14%),[37,41–46] 'critical times' (8%),[47–50] and 'key moments' (4%).[21,51] Otherwise, guidelines either do not use a specific term for defining a key moment (49%) or do not recommend at least one key moment (25%).

Hand hygiene practices

There is agreement among guidelines on what constitutes effective hand hygiene across community settings, although there are gaps in recommendations on how to practice effective hand hygiene. Almost all guidelines (90%) recommend washing hands with soap and for a duration of at least 20 seconds (27%) (Table 1). In addition, 63% of guidelines recommend the use of ABHR as an alternative or complement to handwashing with soap, though very few guidelines recommend a duration for hand rubbing. There is a lack of recommendations on handwashing or hand rubbing technique (16%),[21,40–42,47,52–54] with two of these guidelines (25%, n=2)[21,40] referring to the WHO instructions for hand hygiene in healthcare settings.[28] Meanwhile, 24% of guidelines recommend the inclusion or provision of simple instructions on handwashing technique in hygiene promotion programmes, yet none of these guidelines include specific steps on handwashing or hand rubbing technique.

Guidelines provide inconsistent recommendations on when to practice hand hygiene. Over 30 different individual key moments are recommended among the guidelines. Despite this inconsistency, the individual key moments most commonly mentioned among the guidelines include 'before and

For peer review only

after eating', 'after using the toilet', 'before and after preparing food', 'after blowing nose, coughing, or sneezing', and 'after touching public surfaces or objects' (Table 1). The latter two feature most commonly among guidelines published during the COVID-19 pandemic. Fairly consistently recommended individual key moments include 'when entering or exiting the home or public space', 'after changing a child's diaper', and 'when hands are visibly dirty'. Almost all guidelines recommend clusters of key moments (i.e., individual key moments recommended together). The most common ones are 'after using the toilet' with 'before and after eating' (39%, n=20); 'after using the toilet' with 'before and after preparing food' (24%, n=12); and 'after using the toilet', with 'before and after eating', and 'after blowing nose, coughing, or sneezing' (22%, n=11).

Recommendations for minimum requirements

Handwashing materials

Guidelines consistently recommend plain soap for handwashing across all community settings and paper towels or electric dryers for hand drying in public and institutional settings. Most guidelines (65%) do not recommend a specific type of soap for handwashing, though some specifically recommend bar soap (24%), liquid soap (22%), or "soapy water" (e.g., home-made mixture of powder or liquid soap, or bar soap shavings diluted in water) (16%) (Table 1). No guidelines specifically recommend antibacterial soap. For hand drying after handwashing, most guidelines recommend clean, single use paper towels (39%) and electric air-drying systems (22%) in public and institutional settings. 8% of guidelines recommend a bin with disposal liners and lid for the waste management of paper towels in public and institutional settings.[21,55–57] No guidelines provide a recommendation for hand drying materials in the domestic setting.

Soap and water requirements for handwashing

While guidelines consistently recommend running water for handwashing with soap, there are gaps for soap and water quantity and discordant recommendations on water quality for handwashing. For water services, almost half (43%) of guidelines recommend running water, 16% a piped water system connected to a tap, and 14% water trucking, storage, or manual refilling. Few guidelines recommend a minimum quantity of liquid or bar soap (14%)[43,48,58–61] or water (18%)[12,20,47,50,60–62] required for handwashing (e.g., minimum amount of water needed to wet hands or soap product necessary to cover all surfaces of hands for handwashing with soap) (Table 2). One guideline, for example, recommends less than 5 ml of liquid soap per person per handwashing event,[60] whereas three other guidelines recommend at least 250 grams of bar soap per person per month for handwashing.[43,48,61] For water quality, 16% of guidelines state that water must be of drinking water quality, in line with WHO guidelines.[21,40,41,52,54,63–66] In contrast, 8% state that it does not have to be of drinking water quality,[12,20,39,67] though none specify a quantitative standard nor whether non-drinking quality water may conditionally be used if high quality water is not available. In addition, 10% of guidelines recommend that free residual chlorine must be greater than or equal to 0.5 mg/l after at least 30 minutes of contact time.[43,48,63,68,69]

For peer review only

Table 1. Consistent and fairly consistent recommendations for hand hygiene in community settings.

Parameter	Recommendation	All guidelines (n)	Domestic setting (n)	Public setting (n)	Institutional setting (n)	Multiple settings (n)
Number of guidelines		51	2	24	22	5
**Effective hand hygiene**						
Key moments	Before and after eating	45% (23)	0% (0)	38% (9)	27% (14)	0% (0)
	After using the toilet	41% (21)	0% (0)	38% (9)	55% (12)	0% (0)
	Before and after preparing food	27% (14)	0% (0)	38% (9)	18% (4)	20% (1)
	After blowing nose, coughing, or sneezing	24% (12)	0% (0)	21% (5)	27% (6)	20% (1)
	After touching public surfaces or objects	24% (12)	0% (0)	12% (6)	18% (4)	40% (2)
	When entering/exiting buildings or home	14% (7)	0% (0)	17% (4)	5% (1)	40% (2)
	After changing a child's diaper	12% (6)	0% (0)	17% (4)	0% (0)	40% (2)
	When hands visibly dirty	10% (5)	0% (0)	13% (3)	9% (2)	0% (0)
	Before and after work	8% (4)	0% (0)	0% (0)	18% (4)	0% (0)
	After contact with animals	8% (4)	0% (0)	13% (3)	5% (1)	0% (0)
	Taking care of sick person	8% (4)	0% (0)	13% (3)	5% (1)	0% (0)
Handwashing duration	At least 20 seconds	27% (14)	0% (0)	21% (5)	36% (8)	20% (1)
**Minimum requirements for effective hand hygiene**						
**Materials for effective hand hygiene**						
Soap	Soap	65% (33)	100% (2)	50% (12)	82% (18)	20% (1)
	Bar soap	24% (12)	0% (0)	14% (7)	14% (3)	40% (2)
	Liquid soap	22% (11)	0% (0)	14% (7)	14% (3)	20% (1)
	Soap water (e.g., powder, liquid, or bar soap shavings diluted in water)	16% (8)	0% (0)	17% (4)	5% (1)	60% (3)
ABHR	ABHR with at least 60% alcohol	35% (18)	0% (0)	16% (8)	45% (10)	0% (0)
	ABHR, no alcohol percentage	22% (11)	50% (1)	17% (4)	23% (5)	20% (1)
Hand drying	Clean, single use paper towels	39% (20)	0% (0)	14% (7)	59% (13)	0% (0)
	Air-drying system	22% (11)	0% (0)	17% (4)	14% (7)	0% (0)
	Bin with disposable liners and lid	8% (4)	0% (0)	4% (1)	14% (3)	0% (0)
**Alternative materials**						
Other materials	Ash	22% (11)	0% (0)	12% (6)	14% (3)	40% (2)
Conditional recommendations	ABHR where soap and water not available	18% (9)	0% (0)	4% (1)	36% (8)	0% (0)
	Ash and water if ABHR or soap not available	14% (7)	0% (0)	13% (3)	14% (3)	20% (1)

For peer review only

	ABHR if hands not visibly soiled	10% (5)	0% (0)	8% (2)	14% (3)	0% (0)
**Water requirements**						
Water services	Running water	43% (22)	0% (0)	16% (8)	55% (12)	40% (2)
	Piped water system connected to tap	16% (8)	0% (0)	13% (3)	18% (4)	20% (1)
	Water trucking, storage, or manual refilling	14% (7)	0% (0)	13% (3)	14% (3)	20% (1)
Alternative water sources for handwashing	Rainwater	8% (4)	0% (0)	17% (4)	0% (0)	0% (0)
Wastewater management	Drainage system	16% (8)	0% (0)	8% (2)	23% (5)	20% (1)
	Covered soakaway pit	12% (6)	0% (0)	21% (5)	0% (0)	20% (1)
**Hand hygiene stations**						
Handwashing station	Washbasin	16% (8)	0% (0)	13% (3)	18% (4)	20% (1)
	Bucket or container connected to tap	14% (7)	50% (1)	17% (4)	5% (1)	40% (2)
	Tippy tap	12% (6)	0% (0)	4% (1)	14% (3)	40% (2)
	Foot-pedal or hand-operated system	8% (4)	0% (0)	8% (2)	5% (1)	20% (1)
Location of handwashing stations	Key entry/exit points	27% (14)	0% (0)	50% (12)	5% (1)	20% (1)
	Close proximity to toilets	27% (14)	0% (0)	21% (5)	14% (7)	40% (2)
	Food preparation and eating areas	18% (9)	0% (0)	8% (2)	27% (6)	20% (1)
Accessibility	Accessible for all users and vulnerable groups	12% (6)	0% (0)	13% (3)	5% (1)	40% (2)
	Height of soap and water taps appropriate for children, elderly, and disabled	10% (5)	0% (0)	13% (3)	5% (1)	20% (1)
	Age-appropriate handwashing stations	8% (4)	0% (0)	0% (0)	18% (4)	0% (0)
Design considerations	Theft resistance	16% (8)	0% (0)	17% (4)	14% (3)	20% (1)
	Water-saving designs/technologies	12% (6)	0% (0)	13% (3)	9% (2)	20% (1)
COVID-19 adaptations	Taps that limit cross-contamination	16% (8)	0% (0)	14% (7)	0% (0)	20% (1)
	1-2m spacing between handwashing stations	12% (6)	0% (0)	17% (4)	5% (1)	20% (1)
	Disinfect taps regularly	10% (5)	0% (0)	17% (4)	0% (0)	20% (1)
	Towels for opening/closing taps	8% (4)	0% (0)	13% (3)	5% (1)	0% (0)
Availability of materials	Locally available materials for handwashing	10% (5)	0% (0)	8% (2)	9% (2)	20% (1)
**Behaviour change**						
Behaviour change techniques[1]	Prompts and cues	12% (6)	0% (0)	13% (3)	14% (3)	0% (0)
	Habit formation	8% (4)	0% (0)	13% (3)	5% (1)	0% (0)
Content of behaviour change messaging	Doable instructions with proper steps	24% (12)	0% (0)	12% (6)	27% (6)	0% (0)
	Messages that target motivation	10% (5)	0% (0)	8% (2)	14% (3)	0% (0)

Delivery channels	Visual reminders	31% (16)	0% (0)	12% (6)	36% (8)	40% (2)
	Mass communication	24% (12)	0% (0)	14% (7)	9% (2)	60% (3)
	Small group activities	18% (9)	0% (0)	12% (6)	9% (2)	20% (1)
	Interpersonal communication	10% (5)	0% (0)	17% (4)	0% (0)	20% (1)
Formative research	Identify determinants of target behaviour within target population	8% (4)	0% (0)	4% (1)	0% (0)	60% (3)

[1] Using a typology of behaviour change techniques developed by Michie et al. (2013)[70]

For peer review only

Table 2. Parameters with gaps in recommendations (i.e., fewer than 10 recommendations, equivalent to less than 20% of guidelines providing a recommendation).

Parameters with gaps in recommendations[1]	Percentage (%) of guidelines that provide a recommendation (n)
**Effective hand hygiene**	
Definition of hand hygiene	4% (2)
Definition of safe/effective hand hygiene	6% (3)
Handwashing knowledge	16% (8)
Hand rubbing duration	6% (3)
**Minimum requirements for effective hand hygiene**	
**Soap and water requirements for handwashing**	
Quantity of soap	14% (7)
Quantity of water	18% (9)
**Hand hygiene stations**	
Supply chain for products and materials	6% (3)
Functionality	10% (5)
Cost and affordability	14% (7)
Durability	16% (8)
Operation and maintenance – responsibility	16% (8)
Operation and maintenance – actions	18% (9)
Spacing and number of users per handwashing station	18% (9)
**Behaviour and behaviour change**	
Frequency of behaviour change interventions	4% (2)
Behaviour change approaches	18% (9)
**Government measures**	
Sector policy and strategy	0% (0)
Sector financing	0% (0)
Capacity development	2% (1)
Equity	2% (1)
Planning, monitoring, and review	4% (2)
Institutional arrangements	10% (5)

[1] Parameters with less than 20% of guidelines that provide a recommendation

Alternative materials for hand hygiene

There are consistent recommendations for ABHR as an alternative material for hand hygiene. Of the guidelines that recommend ABHR, 35% (n=18) recommend ABHR with at least 60% alcohol, 22% (n=11) do not specify an alcohol percentage, and only 6% (n=3) recommend an alcohol percentage of at least 70%. In addition, 18% of guidelines recommend ABHR where soap and water are not available[37–39,41,42,52–54,71] and 10% (n=5) only if hands are not visibly soiled.[21,36,40,65,72]

There are discordant recommendations for the use of ash. 22% of guidelines recommend ash as an alternative material to soap for handwashing.[12,36,74,43,45,47,52,64,65,72,73] In addition, 14% recommend ash if ABHR or soap are not available.[12,43,45,47,52,64,74] However, 6% (n=3) of guidelines advise against the use of ash or other products, such as soil, sand, mud, or water alone.[45,47,62] In addition, while some guidelines (8%) recommend 0.05% chlorine solution,[48,53,54,63] one guideline advises against it.[13]

When disaggregated by setting, ABHR is most consistently recommended in public and institutional settings where it may meet larger and more frequent demand than handwashing stations. Similarly, recommendations for the conditional use of ABHR where soap and water are not available feature the

For peer review only

most in the institutional setting, particularly in the workplace and schools. Ash is most commonly recommended in the public setting, which includes low-resource and water-scarce settings where soap and water may not be available.

Hand hygiene stations

Overall, there are inconsistent recommendations on hand hygiene facilities and their location, as well as gaps. Across all settings, guidelines recommend washbasins (e.g., ceramic, cement, or plastic) (16%), a bucket or container connected to tap (14%), and tippy taps (12%) for handwashing stations (Table 1). Guidelines specify 16 different locations for handwashing stations, which include by the entrance and exit of public spaces and buildings (e.g., restaurants, shops, markets, places of worship, train and bus stations) (27%), in close proximity to toilets (27%), and next to food preparation and eating areas (18%). Guidelines also mention placing hand hygiene stations, such as ABHR dispensers, at key entry and exit points of public spaces and buildings (14%). There are inconsistent recommendations on the optimal spacing and number of users per handwashing station, whereas none for ABHR dispensers. Gaps in recommendations include those for hand hygiene materials and product supply chains, cost and affordability, functionality, durability, and hand hygiene station operation and maintenance responsibilities and actions (Table 2).

Recommendations on the location of handwashing stations vary slightly by setting. Close proximity to toilets is consistently recommended for both public and institutional settings (10% and 14%, respectively). By the entrance and exit of public spaces is most consistently recommended for public settings (24%). Next to food preparation and eating areas is most consistently recommended for institutional settings (12%), such as schools and the workplace.

Hand hygiene station access and adaptations

Guidelines recommend hand hygiene stations that are accessible for all and adapted for pandemic response. For example, 10% of guidelines recommend that the height of soap and water taps be appropriate for access by children, the elderly, and disabled (e.g., 500-700 mm basin height for children and 850 mm basin height for wheelchair access) (Table 1). 45% (n=23) of guidelines recommend COVID-19-related adaptations for hand hygiene stations, such as taps that limit cross contamination (16%), one to two meters spacing between stations (12%), regular tap disinfection (10%), and towels for opening and closing taps (8%). Other adaptations include theft resistance (e.g., attaching soap or other movable pieces to the station) (16%) and water-saving designs (e.g., low-flow faucets) (12%).

Recommendations for behaviour change

Overall, there are few recommendations related to hand hygiene behaviour change, though there are consistent recommendations on behaviour change messaging and delivery channels. For behaviour change messaging, some guidelines (10%) recommend messages that target "motives"[75] (Table 1).[20,45,54,64,72] In terms of delivery channels, 31% of guidelines recommend using visual reminders (e.g., signs, posters, or leaflets) and 24% mass communication (e.g., radio, social media, or mobile phone text messaging) to deliver behaviour change messages. Prompts, cues and habit formation are fairly consistently recommended as behaviour change techniques.[20,39,52,67,70,72,76–78] Formative research for behaviour change programmes is recommended by 10% (n=5) of guidelines,[46,47,49,73,76] though only 8% (n=4) specifically mention undertaking formative research to identify behavioural determinants among the target population.[46,47,49,73] Guidelines provide inconsistent recommendations on behaviour change approaches (18%), determinants of hand hygiene to target for interventions (22%), and behaviour

For peer review only

change models or frameworks (33%). Lastly, there are gaps in recommendations on frequency of behaviour change interventions (i.e., how often an intervention should be delivered) and behaviour change approaches (4%)[20,46] (Table 2).

Recommendations for government measures

Overall, few guidelines (22%, n=11) provide a recommendation on government measures. Nonetheless, recommendations for sector policy and strategy include promoting local soap production and fostering public-private partnerships for handwashing (4%).[47,49] For institutional arrangements, 4% of guidelines suggest identifying ways of cross-sectoral collaboration for hand hygiene,[20,49] while other recommendations centre on engaging communities, the private sector, and civil society for the delivery of water, sanitation, and hygiene (WASH) services.[12,65] On planning, monitoring, and review, 4% of guidelines recommend supporting or reinforcing existing monitoring systems or creating a government-led national monitoring system, in line with global hygiene indicators.[49,68]

Evidence-based recommendations

Over 90% of recommendations do not have a citation for one of the five levels of evidence identified for the review (Figure 3). Of the 923 extracted recommendations, only 7% (n=68) indicate whether the recommendation is evidence-based (i.e., cited by one of the five levels of evidence, i.e., systematic review, randomised and quasi-randomised trial, guideline developed from systematic reviews, observational study, or anecdotal information). Less than 1% (n=4) of recommendations cite evidence from a systematic review.[46] In addition, less than 1% (n=3) cite evidence from a randomised or quasi-randomised trial,[12,36] while over 1% (n=11) cite evidence from observational studies, which are mainly recommendations for alternative hand hygiene materials, such as ash, sand, and soil, or alternative water sources for handwashing, such as cooking water, laundry water, bathwater, and seawater.[12,36,47] Less than 1% (n=6) of recommendations cite other guidelines developed from systematic reviews,[21,47,67,71] while 2.5% (n=23) cite WHO interim guidelines for hand hygiene in the context of COVID-19.[51,56,57,65,72,78,79] The other 2% (n=21) of recommendations cite anecdotal information, such as programme documentation.[45,47,62,76] The remaining recommendations (93%, n=855) lack any citation.

DISCUSSION

We identified 51 guidelines published between 1999 and 2021 by various international agencies covering a range of community settings. Most guidelines target the public and institutional setting, while surprisingly none exclusively target the domestic setting. Overall, community settings are not clearly defined among the guidelines, which presents an opportunity for future normative guidelines to establish a clear and common definition, especially as it relates to hand hygiene. Overall, no guidelines comprehensively address hand hygiene across domestic, public, and institutional settings, and very few recommendations are evidence-based, highlighting a gap in global normative guidance on hand hygiene in community settings.

Effective hand hygiene

There is agreement among guidelines on what constitutes effective hand hygiene, however, inconsistencies and gaps remain as to when and how to practice hand hygiene. Current recommendations for handwashing with soap reflect findings from published literature, which show that handwashing with soap is an effective means for preventing the transmission of a range of diseases.[3–9] Regarding when to practice hand hygiene, future guidelines could focus key moments

For peer review only

on those most likely to interrupt the transmission of infectious diseases in domestic, public, and institutional settings. These might include “after using the toilet” to reduce the transmission of diarrhoea-causing pathogens,[9] and “after touching high-contact surfaces” or “after coughing or sneezing” to reduce the risk of respiratory infections.[3] Lastly, gaps in recommendations on handwashing and hand rubbing technique present an opportunity for future guidelines to recommend the optimal technique for reducing bacterial load on hands.

Minimum requirements

Consistent recommendations for the use of soap and water and ABHR suggest the widespread acceptability of these materials for effective hand hygiene across community settings. Current recommendations for the use of plain soap are consistent with findings from a systematic review that suggest that plain soap is more effective than antibacterial soap at removing or inactivating pathogens on hands in community settings.[80] Recommendations for running water are equally relevant as unreliable water supply negatively affects households’ ability to perform hand hygiene.[81] Gaps in recommendations on minimum quantities of soap and water required for effective hand hygiene are important for future guidelines to address. The recommended use of ABHR with at least 60% alcohol is in line with several studies which have found that ABHRs with an alcohol concentration between 60 and 80% are more effective at killing germs than those with a lower alcohol percentage, particularly in clinical settings.[82,83] Although ABHR can still inactivate many types of microbes when used correctly, evidence suggests that soap and running water are still more effective at removing certain types of pathogens that may be present on hands.[84] The WHO Guidelines on Hand Hygiene in Health Care, for example, recommend ABHR as the preferred method for routine hand hygiene in healthcare settings when hands are not visibly soiled, as it enables more frequent hand hygiene.[28] Similarly, in community settings, ABHR may be suitable in contexts where frequent hand hygiene is necessary, such as transport hubs and entrances or exits to public spaces and buildings.[85] ABHR may also be favourable where frequent hand hygiene is required, as the repeated use of soap can result in skin dryness and cause chronic irritant contact dermatitis.[28] However, in certain domestic, public, and institutional settings, such as the household or schools, where hands may become more soiled, ABHR may be less likely to effectively inactivate microbes.[82,83] As per current international guideline recommendations, handwashing with soap may therefore be prioritised in these settings, with ABHR as a suitable complement or alternative where frequent hand hygiene is required. Finally, because the transmission of germs is more likely to occur to and from wet hands, hand drying is an essential component of effective hand hygiene, especially for handwashing.[86] Current recommendations for hand drying in public and institutional settings are consistent with those in the WHO Guidelines on Hand Hygiene in Health Care, which recommend that hands should ideally be dried with individual paper towels, otherwise with air dryers.[28] While there is mixed evidence for the most effective hand drying method,[87] the WHO recommendations are based on findings that suggest that paper towels may effectively prevent re-contamination of hands, while also lowering the risk of spreading pathogens through the air compared to electric air dryers.[86]

Discordant recommendations for minimum requirements suggest the need to leverage further research to determine the optimal water quality for handwashing and effectiveness of alternative materials for hand hygiene where soap and ABHR are not widely available. Limited evidence suggests that the use of non-potable water with low-to-moderate levels of *E. coli* contamination may still be effective for handwashing,[88] which may be promising for areas where it is difficult to regularly treat water or where there is intermittent water supply that is prone to contamination. Similarly, two studies found that drinkable water may not be needed for handwashing with soap.[89,90] Nevertheless, the WHO Guidelines on Hand Hygiene in Health Care recommend washing hands with clean, running water whenever possible.[28] While there are discordant recommendations for the use of ash, there is uncertain evidence whether this stops or reduces the spread of pathogens compared

For peer review only

to hand cleansing with soap, mud, soil, or no hand cleansing.[91] Future guidelines may consider the efficacy of hand hygiene products along with their availability and acceptability in domestic, public, and institutional settings to make relevant recommendations, particularly in water-scarce regions or settings where there is limited access to soap or ABHR. For example, soapy water may be a promising low-cost and effective alternative to bar soap in settings where bar or liquid soap is unaffordable.[92] In addition, one interim guideline on hand hygiene practices in low-resource settings, for example, recommends the use of friction-generating materials where clean, running water, soap, or ABHR are not available.[36]

Current inconsistent recommendations for hand hygiene facilities and their placement may limit the practice of effective hand hygiene. Sustaining hand hygiene behaviour change requires consistent access to functional hand hygiene stations at key locations[93,94] and diverse infrastructure is recommended with varying costs. Nonetheless, the appropriateness of these recommendations is likely to depend on the local availability and affordability of materials. One guideline, for example, provides technical recommendations for permanent and semi-permanent handwashing facilities in public places and buildings, focusing on the sustainability and equitable access of these facilities.[20] Similarly, future guidelines may prioritise the accessibility, affordability, and sustainability of materials and infrastructure for hand hygiene across all community settings to address current inconsistent and discordant recommendations. The accessibility and sustainability of hand hygiene stations are particularly important to ensure that they are inclusive and kept functional and well-stocked beyond their installation.[20]

Behaviour change

The gaps in recommendations related to behaviour change suggest the need for guidance based on established behavioural theory and existing evidence. There are some recommendations for behaviour change, though without clear steps on how to develop, implement, and sustain hand hygiene behaviour change interventions. Future guidelines may benefit from leveraging well-established behavioural frameworks and theories[95–97] to make recommendations as to how to develop effective, locally-appropriate strategies beyond information-focused communication. In addition, while most behaviour change theories and frameworks recommended amongst the guidelines note the importance of formative research, very few guidelines recommend undertaking formative research. Yet, formative research plays a key role in adapting hand hygiene behaviour change programmes to high-risk populations and target settings.[98]

Government measures

The significant gaps in recommendations on government measures underscore the current lack of normative standards to guide national governments on the planning, delivery, financing, and monitoring of effective hand hygiene. Indicators also suggest inadequate planning and insufficient funding for hand hygiene among national governments globally.[19] Future guidelines may therefore consider prioritising government measures to support countries in responding to and preventing public health crises, such as the COVID-19 pandemic. Future guidelines may also focus recommendations on hand hygiene monitoring and reporting to improve comparison of hand hygiene indicators within and between countries.

Strengths and limitations

This review has four main limitations. First, as a scoping review, we did not systematically assess the quality of the included guidelines although we did, for example, consider aspects such as the extent to which recommendations were based on cited evidence. In addition, included guidelines covered

For peer review only

WASH and public health topics beyond hand hygiene, so it was therefore not necessarily relevant to assess the whole guidelines for quality, but rather focus on the robustness of the specific recommendations for hand hygiene. Second, the search was limited to guidelines published in English or French and therefore may have excluded relevant guidelines published in other languages. Third, the lack of recommendations for government measures among current international guidelines may not reflect an absolute absence of guidance in this area. Guidance to governments and other stakeholders on how to improve uptake of hand hygiene practices in community settings may be included in other sector documents or policy instruments. Fourth, while recommendations were summarised across domestic, public, and institutional settings, there were often too few recommendations for each setting to assess consistency and concordance. Still, with 51 guidelines providing over 900 recommendations for hand hygiene in community settings, findings from this review highlight significant gaps and inconsistencies across community settings that future guidelines may seek to prioritise.

CONCLUSION

This review identified 51 current international guidelines providing 923 recommendations for hand hygiene in community settings. Nonetheless, there are several important areas of discordance and significant gaps in the recommendations among these guidelines. Furthermore, very few recommendations are supported by any qualifying evidence. The COVID-19 pandemic led to numerous national, regional, and international efforts to improve effective hand hygiene in domestic, public, and institutional settings, such as households, public spaces, workplaces, and schools, but the lack of clear recommendations supported by cited evidence may limit progress in this important area of public health.

For peer review only

Figure captions

Figure 1. Conceptual framework for hand hygiene in community settings.
Figure 2. PRISMA flow diagram.
Figure 3. Percentage of recommendations cited by one of the five types of evidence or no evidence.

For peer review only

Contributors

CM, LB, BC, CC, KC, JC, RD, REN, BG, JEM, and OC informed the study protocol. CM carried out the database and grey literature search with input from OC and JEM. JEM, OC, BG, and CM carried out expert consultations. CM and LB screened the retrieved articles for inclusion and extracted the data with input from OC. CM led on data analysis and CM, LB, OC, and JEM led on the presentation of results with inputs from co-authors. CM, LB, JEM, and OC led on writing the manuscript with input from all co-authors. OC, JEM, and BG provided overall supervision, leadership, and advice. The first author (CM) accepts responsibility for the work.

Funding

This research was funded by the World Health Organization and the UK Foreign, Commonwealth, and Development Office.

Competing interests

The authors declare no competing interests.

Patient consent for publication

Not applicable.

Ethics approval

This research did not require institutional review board approval as the data were publicly available and collected from existing online databases and search engines. This research did not involve any human subjects.

Data availability statement

All data relevant to the study are included in the article or uploaded as supplementary information.

For peer review only

References

1 Burton M, Cobb E, Donachie P, *et al.* The effect of handwashing with water or soap on bacterial contamination of hands. *Int J Environ Res Public Health* 2011;**8**:97–104. doi:10.3390/ijerph8010097

2 Cairncross S, Valdamis V. Water Supply, Sanitation, and Hygiene Promotion. In: *Disease Control Priorities in Developing Countries*. Washington: : World Bank 2006. 771–792.

3 Freeman M, Stocks M, Cumming O, *et al.* Hygiene and health: systematic review of handwashing practices worldwide and update of health effects. *Trop Med Int Health* 2014;**19**:906–16. doi:10.1111/TMI.12339

4 Rabie T, Curtis V. Handwashing and risk of respiratory infections: A quantitative systematic review. *Trop Med Int Heal* 2006;**11**:258–67. doi:10.1111/j.1365-3156.2006.01568.x

5 Aiello AE, Coulborn RM, Perez V, *et al.* Effect of hand hygiene on infectious disease risk in the community setting: A meta-analysis. *Am J Public Health* 2008;**98**:1372–81. doi:10.2105/AJPH.2007.124610

6 Prüss-Ustün A, Wolf J, Bartram J, *et al.* Burden of disease from inadequate water, sanitation and hygiene for selected adverse health outcomes: An updated analysis with a focus on lowand middle-income countries. *Int J Hyg Environ Health* 2019;**222**:765–77.

7 Mbakaya BC, Lee PH, Lee RLT. Hand hygiene intervention strategies to reduce diarrhoea and respiratory infections among schoolchildren in developing countries: A systematic review. *Int J Environ Res Public Health* 2017;**14**:1–14. doi:10.3390/ijerph14040371

8 Wolf J, Hubbard S, Brauer M, *et al.* Effectiveness of interventions to improve drinking water, sanitation, and handwashing with soap on risk of diarrhoeal disease in children in low-income and middle-income settings: a systematic review and meta-analysis. *Lancet* 2022;**400**:48–59. doi:10.1016/S0140-6736(22)00937-0

9 Ejemot-Nwadiaro R, Ehiri J, Arikpo D, *et al.* Hand washing promotion for preventing diarrhoea (review). *Cochrane Database Syst Rev* 2015;**2015**. doi:10.1002/14651858.CD004265.pub3

10 Stocks ME, Ogden S, Haddad D, *et al.* Effect of Water, Sanitation, and Hygiene on the Prevention of Trachoma: A Systematic Review and Meta-Analysis. *PLoS Med* 2014;**11**. doi:10.1371/journal.pmed.1001605

11 Strunz EC, Addiss DG, Stocks ME, *et al.* Water, Sanitation, Hygiene, and Soil-Transmitted Helminth Infection: A Systematic Review and Meta-Analysis. *PLoS Med* 2014;**11**. doi:10.1371/journal.pmed.1001620

12 WHO. Water, sanitation, hygiene, and waste management for SARS-CoV-2, the virus that causes COVID-19. Geneva, Switzerland: 2020. https://www.who.int/publications/i/item/water-sanitation-hygiene-and-waste-management-for-the-covid-19-virus-interim-guidance

13 WHO. Recommendations to member states to improve hand hygiene practices to help prevent the transmission of the COVID-19 virus. Geneva, Switzerland: 2020.

14 Talic S, Shah S, Wild H, *et al.* Effectiveness of public health measures in reducing the incidence of covid-19, SARS-CoV-2 transmission, and covid-19 mortality: systematic review and meta-analysis. *BMJ* 2021;:375. doi:https://doi.org/10.1136/bmj-2021-068302

15 WHO. The Ottawa Charter for Health Promotion. 1986.http://www.who.int/healthpromotion/conferences/previous/ottawa/en/index.html.

16 Brauer M, Zhao J, Bennitt F, *et al.* Global Access to Handwashing: Implications for COVID-19 Control in Low-Income Countries. *Environ Health Perspect* 2020;**128**. doi:https://doi.org/ 10.1289/EHP7200

17 WHO, UNICEF. Progress on household drinking water, sanitation and hygiene 2000-2020: five years into the SDGs. Geneva, Switzerland: 2020.

18 WHO, UNICEF. Progress on drinking water, sanitation and hygiene in schools: special focus on Covid-19. 2020.

For peer review only

19 WHO. Hygiene - UN-Water GLAAS findings on national policies, plans, targets and finance. World Heal. Organ. 2020.https://www.who.int/water_sanitation_health/publications/hygiene-glaas-findings/en/%0Ahttps://www.unwater.org/publications/hygiene-un-water-glaas-findings-on-national-policies-plans-targets-and-finance/
20 WaterAid. Technical Guide for handwashing facilities in public places and buildings. 2020. https://washmatters.wateraid.org/sites/g/files/jkxoof256/files/technical-guide-for-handwashing-facilities-in-public-places-and-buildings.pdf
21 ILO. Hand hygiene at the workplace: an essential occupational safety and health prevention and control measure against COVID-19. Geneva, Switzerland: 2020.
22 ICRC. Water, Sanitation, and Hygiene and Habitat in Prisons: Supplementary Guidance. Geneva, Switzerland: 2012. https://www.icrc.org/eng/assets/files/publications/icrc-002-4083.pdf
23 Arksey H, O'Malley L. Scoping studies: Towards a methodological framework. *Int J Soc Res Methodol* 2005;**8**:19–32. doi:10.1080/1364557032000119616
24 Levac D, Colquhoun H, O'Brien K. Scoping studies: advancing the methodology. *Implement Sci* 2010;**5**. doi:10.1017/cbo9780511814563.003
25 Westphaln KK, Regoeczi W, Masotya M, *et al.* From Arksey and O'Malley and Beyond: Customizations to enhance a team-based, mixed approach to scoping review methodology. *MethodsX* 2021;**8**:101375. doi:10.1016/j.mex.2021.101375
26 Tricco A, Lillie E, Zarin W, *et al.* PRISMA Extension for Scoping Reviews (PRISMA-ScR): Checklist and Explanation. *Ann Intern Med* 2018;**169**:467–73. doi:10.7326/M18-0850
27 MacLeod C, Braun L, Chipungu J, *et al.* Guideline recommendations for hand hygiene in community settings: scoping review protocol. 2021.https://osf.io/z2gvs/?view_only=95516bea2202437d8fbea11be17fcae7
28 WHO. WHO Guidelines on Hand Hygiene in Health Care. Geneva, Switzerland: 2009.
29 Sanitation and Water for All. Building Blocks. 2022.https://www.sanitationandwaterforall.org/about/our-work/priority-areas/building-blocks (accessed 15 Jun 2022).
30 MacQueen K, McLellan E, Metzger D, *et al.* What is Community? An Evidence-Based Definition for Participatory Public Health. *Am J Public Health* 2001;**91**.
31 WHO. WHO Guidelines. 2022.https://www.who.int/publications/who-guidelines#:~:text=A WHO guideline is defined,practice or public health policy. (accessed 15 Jun 2022).
32 Sphere Association. The Sphere Handbook: Humanitarian Charter and Minimum Standards in Humanitarian Response (fourth edition). 2018.www.spherestandards.org/handbook
33 Elsevier. Mendeley.
34 Microsoft. Microsoft Excel Spreadsheet Software.
35 Gugiu PC, Gugiu MR. A critical appraisal of standard guidelines for grading levels of evidence. *Eval Heal Prof* 2010;**33**:233–55. doi:10.1177/0163278710373980
36 WHO. Considerations for community hand hygiene practices in low-resource situations. 2020. http://iris.wpro.who.int/handle/10665.1/14533
37 CDC. Hand Hygiene at Work. 2021.https://www.cdc.gov/handwashing/handwashing-corporate.html (accessed 1 Nov 2021).
38 CDC. Interim Guidance on Management of Coronavirus Disease 2019 (COVID-19) in Correctional and Detention Facilities. 2021.https://www.cdc.gov/coronavirus/2019-ncov/community/correction-detention/guidance-correctional-detention.html (accessed 1 Nov 2022).
39 WASH4Work. Hand hygiene protocol for the workplace. 2020.
40 WHO. Preparedness, prevention and control of COVID-19 in prisons and other places of detention: interim guidance. Copenhagen, Denmark: 2021.
41 CDC. When & How to Wash Your Hands. 2021.https://www.cdc.gov/handwashing/when-

For peer review only

how-handwashing.html (accessed 1 Nov 2021).
42 CDC. Hand Hygiene in School and Early Care and Education. 2021.
43 Global WASH Cluster. Global WASH Cluster – COVID 19 Response Guidance Note # 02 – Update 15 April 2020. 2020. https://reliefweb.int/report/world/global-wash-cluster-covid-19-response-guidance-note-02-update-15-april-2020
44 Global WASH Cluster. Health and Hygiene Promotion Guidance Document for COVID-19 – 29 March 2020. 2020.
45 Oxfam. Handwashing technical briefing note.
46 WHO. Guidelines on sanitation and health. Geneva, Switzerland: 2018. http://www.who.int/water_sanitation_health/publications/guidelines-on-sanitation-and-health/en/
47 Malteser International. WASH Guidelines for Field Practitioners: Hygiene. Cologne, Germany: 2017.
48 UNHCR. Technical WASH Guidance for COVID-19 Preparedness and Response. 2020.
49 UNICEF. Understanding Hygiene promotion in the context of Risk Communication & Community Engagement (RCCE) and Infection Control and Prevention (IPC) for the COVID-19 outbreak. New York, N.Y.: 2020.
50 UNICEF, GIZ. Scaling up Group Handwashing in Schools: Compendium of Group Handwashing Facilities Across the Globe. 2016.
51 WHO. Considerations in adjusting public health and social measures in the context of COVID-19. Geneva, Switzerland: 2020. https://www.who.int/publications-detail/risk-
52 UNICEF, SNV, GIZ. Handwashing with soap in schools: a technical options manual. 2020.
53 WHO, IFRC, UNICEF. Key Messages and Actions for Prevention and Control in Schools. 2020.
54 UNICEF, WHO, IFRC. Interim Guidance for COVID-19 Prevention and Control in Schools. New York, N.Y.: 2020. https://www.unicef.org/reports/key-messages-and-actions-coronavirus-disease-covid-19-prevention-and-control-schools
55 WHO. Public Health for Mass Gatherings: Key Considerations. Geneva, Switzerland: 2015. https://apps.who.int/iris/bitstream/handle/10665/162109/WHO_HSE_GCR_2015.5_eng.pdf;jsessionid=20CDEF66BA427C7DB54004047BD811E4?sequence=1
56 WHO. Safe Eid Al Adha Practices In The Context Of COVID-19 Interim Guidance. Geneva, Switzerland: 2021.
57 WHO. Safe Ramadan practices in the context of COVID-19. Geneva, Switzerland: 2021.
58 ICRC. Water, Sanitation, Hygiene and Habitat in Prisons. Geneva, Switzerland: 2013. www.icrc.org
59 ICRC. Health care in detention. Geneva, Switzerland: 2015. https://www.icrc.org/en/publication/4213-health-care-detention-practical-guide
60 UNICEF. COVID-19 Handwashing with Soap (HWWS) Facilities: Compendium of Indicative Layouts, Designs and Cost Estimates. New York, N.Y.: 2020.
61 Water Mission. Handwashing station program: requirements and recommendations. North Charleston, South Carolina, U.S.: 2020.
62 SNV. Practical options for handwashing stations: a guide for promoters and producers. The Hague, Netherlands: https://snv.org/assets/explore/download/202012-practical-_options-for-handwashing-stations.pdf
63 UNICEF. COVID-19 Emergency Preparedness and Response WASH and Infection Prevention and Control Measures in Schools. New York, New York: 2020.
64 WHO. Water, sanitation and hygiene standards for schools in low-cost settings. Geneva, Switzerland: 2009. doi:10.1080/00207233.2014.913878
65 WHO. Actions for consideration in the care and protection of vulnerable population groups from COVID-19. Geneva, Switzerland: 2021. http://iris.wpro.who.int/handle/10665.1/14549%0Ahttps://apps.who.int/iris/handle/10665/333043

For peer review only

66 WHO. Guidelines for drinking-water quality, 4th edition. 2017.
67 UNICEF. Handwashing Stations and Supplies for the COVID-19 response. New York, New York: 2020.
68 GOAL. WASH and Infection Prevention and Control (IPC) Measures in Households and Public spaces. 2020.
69 UNICEF. WASH and Infection Prevention and Control (IPC) Measures in Households and Public Spaces. New York, N.Y.: 2020.
70 Michie S, Richardson M, Johnston M, *et al.* The behavior change technique taxonomy (v1) of 93 hierarchically clustered techniques: building an international consensus for the reporting of behavior change interventions. *Ann Behav Med* 2013;**46**:81–95. doi:10.1007/s12160-013-9486-6
71 ILO. Safe return to work: Guide for employers on COVID-19 prevention. Geneva, Switzerland: 2020.
72 WHO. Guidance on COVID-19 for the care of older people and people living in long-term care facilities, other non-acute care facilities and home care. 2020.
73 UNICEF. A Manual on Hygiene Promotion. New York, N.Y.: 1999. http://www.unicef.org/wash/files/hman.pdf
74 WHO. WHO recommended strategies for the prevention and control of communicable diseases. Geneva, Switzerland: 2001.
75 Curtis VA, Danquah LO, Aunger R V. Planned, motivated and habitual hygiene behaviour: An eleven country review. *Health Educ Res* 2009;**24**:655–73. doi:10.1093/her/cyp002
76 SNV. Behaviour Change Communication Guidelines. Thimphu, Bhutan: 2016. https://snv.org/cms/sites/default/files/explore/download/snv_behaviour_change_communication_guidelines_-_april_2016.pdf
77 UNHCR. UNHCR Hygiene Promotion Guidelines. 2017. https://wash.unhcr.org/download/hygiene-promotion-guidelines-unhcr-2017/
78 WHO, ILO. Preventing and mitigating COVID-19 at work. Geneva, Switzerland: 2021. https://www.who.int/publications/i/item/WHO-2019-nCoV-workplace-actions-policy-brief-2021-1
79 WHO. Key Planning Recommendations for Mass Gatherings in the Context of COVID-19. Geneva, Switzerland: 2020.
80 Aiello AE, Larson EL, Levy SB. Consumer antibacterial soaps: Effective or just risky? *Clin Infect Dis* 2007;**45**. doi:10.1086/519255
81 Kumpel E, Billava N, Nayak N, *et al.* Water use behaviors and water access in intermittent and continuous water supply areas during the COVID-19 pandemic. *J Water Health* 2022;**20**:139–48. doi:10.2166/WH.2021.184
82 Todd E, Michaels B, Holah J, *et al.* Outbreaks where food workers have been implicated in the spread of foodborne disease. Part 10. Alcohol-based antiseptics for hand disinfection and a comparison of their effectiveness with soaps. *J Food Prot* 2010;**73**:2128–40.
83 Charbonneau D, Ponte J, Kochanowski B. A method of assessing the efficacy of hand sanitizers: use of real soil encountered in the food service industry. *J Food Prot* 2000;**63**:495–501.
84 Burton M, Cobb E, Donachie P, *et al.* The effect of handwashing with water or soap on bacterial contamination of hands. *Int J Environ Res Public Health* 2011;**8**:97–104.
85 Howard G, Bartram J, Brocklehurst C, *et al.* COVID-19: Urgent actions, critical reflections and future relevance of ‘WaSH’: Lessons for the current and future pandemics. *J Water Health* 2020;**18**:613–30. doi:10.2166/wh.2020.162
86 Huang C, Ma W, Stack S. The hygienic efficacy of different hand-drying methods: A review of the evidence. *Mayo Clin Proc* 2012;**87**:791–8. doi:10.1016/j.mayocp.2012.02.019
87 Gustafson D, Vetter E, Larson D, *et al.* Effects of 4 hand-drying methods for removing bacteria from washed hands: a randomized trial. *Mayo Clin Proc* 2000;**75**:705–8.

For peer review only

88 Verbyla ME, Pitol AK, Navab-Daneshmand T, *et al.* Safely Managed Hygiene: A Risk-Based Assessment of Handwashing Water Quality. *Environ Sci Technol* 2019;**53**:2852–61. doi:10.1021/acs.est.8b06156
89 Hoque B. Handwashing practices and challenges in Bangladesh. *Int J Environ Health Res* 2004;**13**:S81–7. doi:https://doi.org/10.1080/0960312031000102831
90 Luby S, Agboatwalla M, Painter J, *et al.* Effect of intensive handwashing promotion on childhood diarrhea in high-risk communities in Pakistan: A randomized controlled trial. *J Am Med Assoc* 2004;**291**:2547–54. doi:10.1001/jama.291.21.2547
91 Paludan-Müller AS, Boesen K, Klerings I, Jørgensen KJ MK. Hand cleaning with ash for reducing the spread of viral and bacterial infections: a rapid review. *Cochrane Database Syst Rev* Published Online First: 2020. doi:10.1002/14651858.CD013597.www.cochranelibrary.com
92 Amin N, Pickering AJ, Ram PK, *et al.* Microbiological evaluation of the efficacy of soapy water to clean hands: A randomized, non-inferiority field trial. *Am J Trop Med Hyg* 2014;**91**:415–23. doi:10.4269/ajtmh.13-0475
93 United Nations Children's Fund (UNICEF), World Health Organization (WHO). *State of the World's Hand Hygiene: A global call to action to make hand hygiene a priority in policy and practice*. New York, New York: : UNICEF 2021.
94 White S, Thorseth AH, Dreibelbis R, *et al.* The determinants of handwashing behaviour in domestic settings: An integrative systematic review. *Int J Hyg Environ Health* 2020;**227**:113512. doi:10.1016/J.IJHEH.2020.113512
95 Michie S, Stralen M van, West R. The behaviour change wheel: A new method for characterising and designing behaviour change interventions. *Implement Sci* 2011;**6**. doi:10.1001/archderm.1985.01660070119033
96 Aunger R, Curtis V. Behaviour Centred Design: towards an applied science of behaviour change. *Health Psychol Rev* 2016;**10**:425–46. doi:10.1080/17437199.2016.1219673
97 West R, Michie S. A brief introduction to the COM-B Model of behaviour and the PRIME Theory of motivation. 2020.https://www.qeios.com/read/WW04E6.2
98 Curtis V, Kanki B, Cousens S, *et al.* Dirt and diarrhoea formative research in hygiene promotion programmes. *Health Policy Plan* 1997;**12**:122–31.

For peer review only

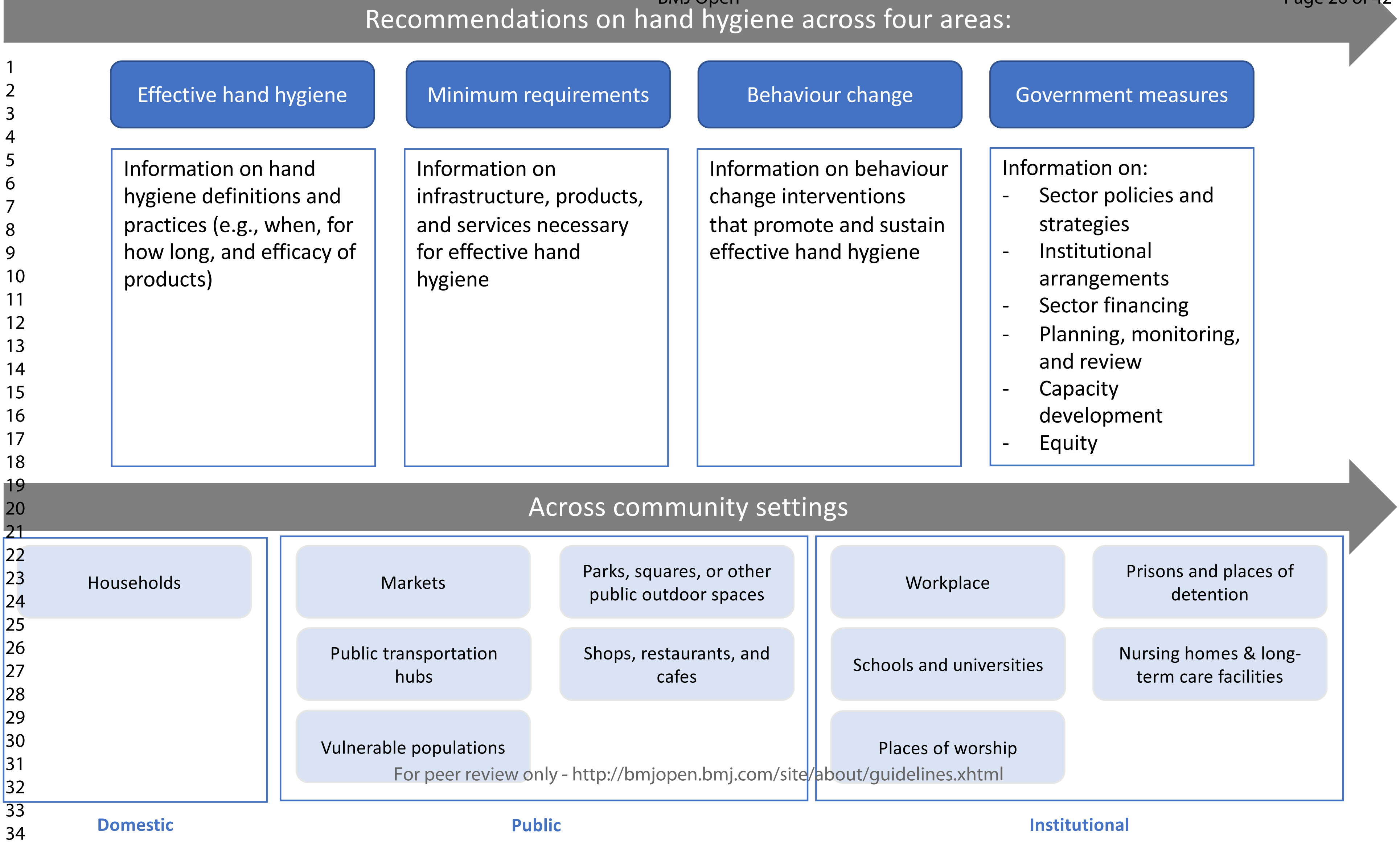
Recommendations on hand hygiene across four areas:
Effective hand hygiene
Minimum requirements
Behaviour change
Government measures
Information on hand hygiene definitions and practices (e.g., when, for how long, and efficacy of products)
Information on infrastructure, products, and services necessary for effective hand hygiene
Information on behaviour change interventions that promote and sustain effective hand hygiene
Information on:
- Sector policies and strategies
- Institutional arrangements
- Sector financing
- Planning, monitoring, and review
- Capacity development
- Equity
Across community settings
Households
Markets
Parks, squares, or other public outdoor spaces
Public transportation hubs
Shops, restaurants, and cafes
Vulnerable populations
Workplace
Prisons and places of detention
Schools and universities
Nursing homes & long-term care facilities
Places of worship
Domestic
Public
Institutional

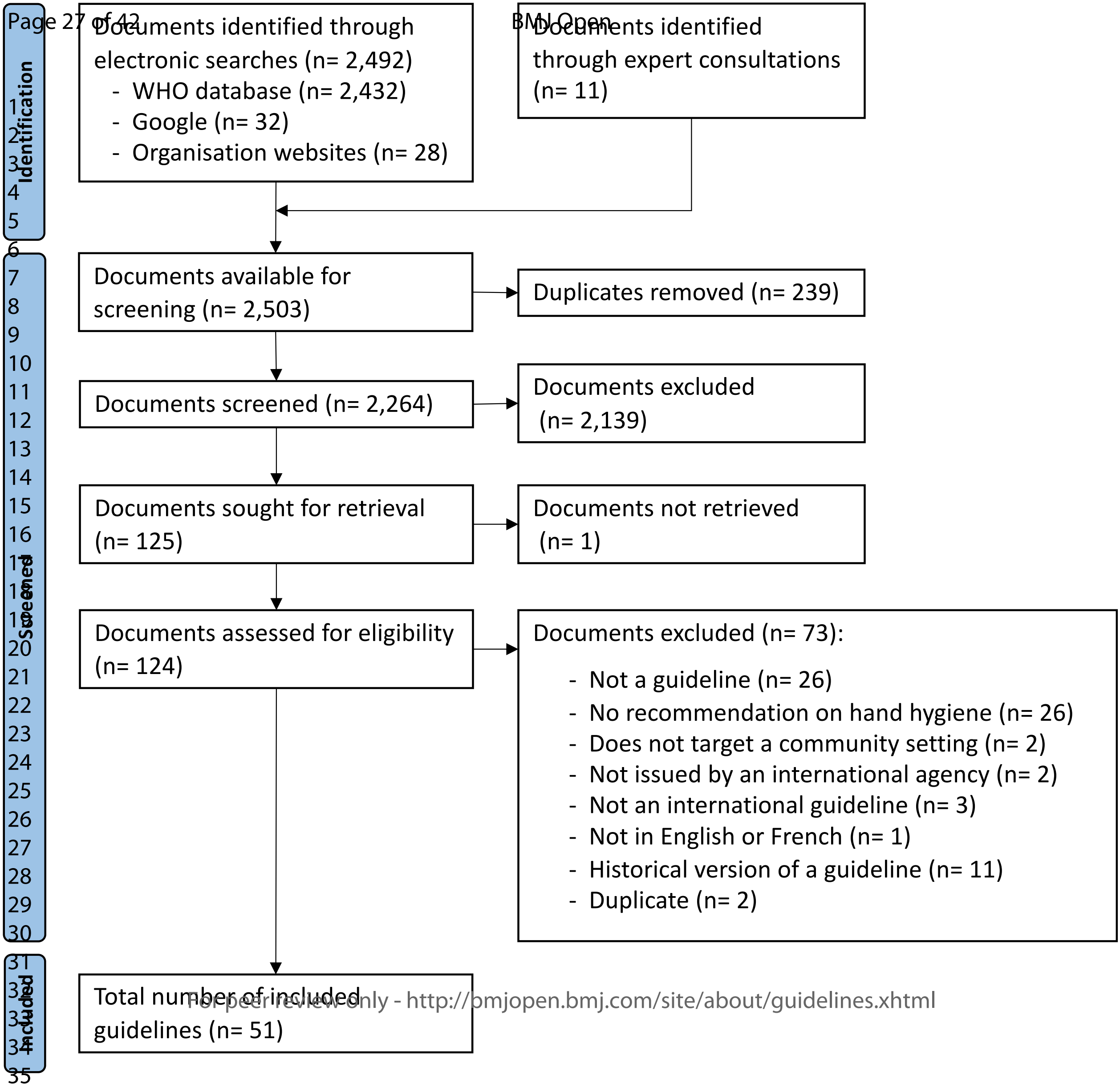

For peer review only - http://bmjopen.bmj.com/site/about/guidelines.xhtml

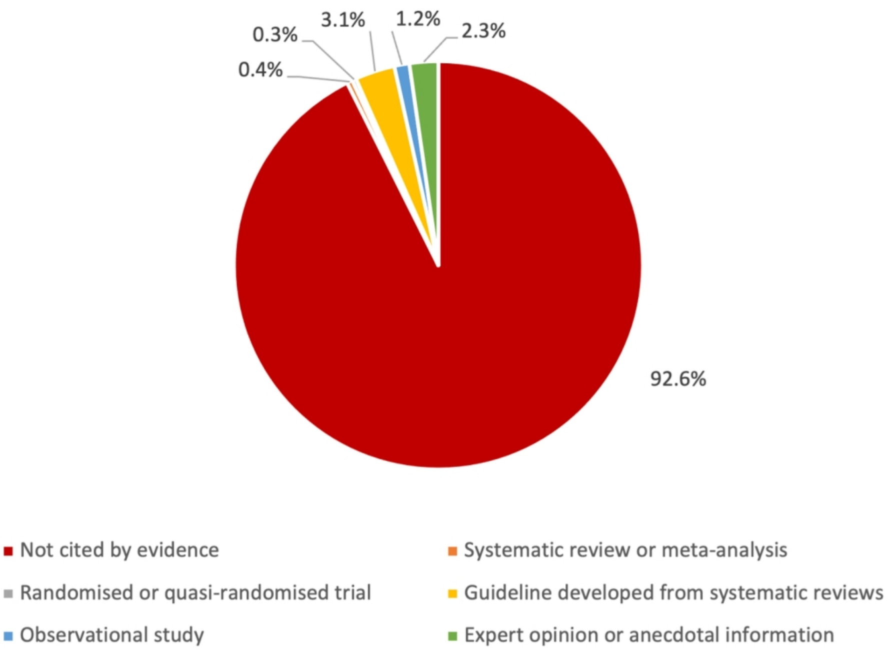

Percentage of recommendations cited by one of the five types of evidence or no evidence.

140x92mm (300 x 300 DPI)

SUPPLEMENTAL MATERIALS

Table A1. The completed Preferred Reporting Items for Systematic reviews and Meta-Analyses extension for Scoping Reviews (PRISMA-ScR) checklist.[25]

SECTION	ITEM	PRISMA-ScR CHECKLIST ITEM	REPORTED ON PAGE #
**TITLE**			
Title	1	Identify the report as a scoping review.	2
**ABSTRACT**			
Structured summary	2	Provide a structured summary that includes (as applicable): background, objectives, eligibility criteria, sources of evidence, charting methods, results, and conclusions that relate to the review questions and objectives.	3
**INTRODUCTION**			
Rationale	3	Describe the rationale for the review in the context of what is already known. Explain why the review questions/objectives lend themselves to a scoping review approach.	5
Objectives	4	Provide an explicit statement of the questions and objectives being addressed with reference to their key elements (e.g., population or participants, concepts, and context) or other relevant key elements used to conceptualize the review questions and/or objectives.	5
**METHODS**			
Protocol and registration	5	Indicate whether a review protocol exists; state if and where it can be accessed (e.g., a Web address); and if available, provide registration information, including the registration number.	5
Eligibility criteria	6	Specify characteristics of the sources of evidence used as eligibility criteria (e.g., years considered, language, and publication status), and provide a rationale.	6
Information sources	7	Describe all information sources in the search (e.g., databases with dates of coverage and contact with authors to identify additional sources), as well as the date the most recent search was executed.	6
Search	8	Present the full electronic search strategy for at least 1 database, including any limits used, such that it could be repeated.	Supplemental material Table A5
Selection of sources of evidence	9	State the process for selecting sources of evidence (i.e., screening and eligibility) included in the scoping review.	6
Data charting process	10	Describe the methods of charting data from the included sources of evidence (e.g., calibrated forms or forms that have been tested by the team before their use, and whether data charting was done independently or in duplicate) and any processes for obtaining and confirming data from investigators.	6
Data items	11	List and define all variables for which data were sought and any assumptions and simplifications made.	6
Critical appraisal of individual sources of evidence	12	If done, provide a rationale for conducting a critical appraisal of included sources of evidence; describe the methods used and how this information was used in any data synthesis (if appropriate).	6 – 7

SECTION	ITEM	PRISMA-ScR CHECKLIST ITEM	REPORTED ON PAGE #
Synthesis of results	13	Describe the methods of handling and summarizing the data that were charted.	6 – 7
**RESULTS**			
Selection of sources of evidence	14	Give numbers of sources of evidence screened, assessed for eligibility, and included in the review, with reasons for exclusions at each stage, ideally using a flow diagram.	7
Characteristics of sources of evidence	15	For each source of evidence, present characteristics for which data were charted and provide the citations.	Supplemental material Table A6
Critical appraisal within sources of evidence	16	If done, present data on critical appraisal of included sources of evidence (see item 12).	14
Results of individual sources of evidence	17	For each included source of evidence, present the relevant data that were charted that relate to the review questions and objectives.	7 – 14
Synthesis of results	18	Summarize and/or present the charting results as they relate to the review questions and objectives.	7 – 14
**DISCUSSION**			
Summary of evidence	19	Summarize the main results (including an overview of concepts, themes, and types of evidence available), link to the review questions and objectives, and consider the relevance to key groups.	14 – 16
Limitations	20	Discuss the limitations of the scoping review process.	16
Conclusions	21	Provide a general interpretation of the results with respect to the review questions and objectives, as well as potential implications and/or next steps.	6
**FUNDING**			
Funding	22	Describe sources of funding for the included sources of evidence, as well as sources of funding for the scoping review. Describe the role of the funders of the scoping review.	17

For peer review only

Table A2. List of websites of international organisations known to work on hand hygiene searched.

Agency or resource hub	Website
Africa Centers for Disease Control and Prevention (CDC)	https://africacdc.org/
Global Handwashing Partnership	https://globalhandwashing.org/
Global WASH Cluster	www.washcluster.org
International Red Cross and Red Crescent (ICRC)	www.icrc.org
International Federation of the Red Cross (IFRC)	www.ifrc.org
International Labour Organization (ILO)	https://www.ilo.org
International Centre for Diarrhoeal Disease Research Bangladesh (ICDDR'B)	www.icddrb.org
Oxfam	www.oxfam.org.uk
Sanitation Learning Hub	https://sanitationlearninghub.org/
Save the Children	www.nrc.no
SNV Netherlands Development Organisation	https://snv.org/
Sustainable Sanitation Alliance	https://www.susana.org/en/
United Nations Human Settlement Programme (UN Habitat)	https://unhabitat.org/
United Nations High Commissioner for Refugees (UNHCR)	www.unhcr.org
United Nations Children's Fund (UNICEF)	www.unicef.org
US Centers for Disease Control and Prevention (CDC)	www.cdc.gov
WASH'Em	https://www.washem.info/
WaterAid	https://www.wateraid.org/us/
World Bank	https://www.worldbank.org/en/home
Water, Education and Development Centre (WEDC)	www.wedc.lboro.ac.uk
Water and Sanitation for the Urban Poor (WSUP)	https://www.wsup.com/
Deutsche Gesellschaft für Internationale Zusammenarbeit (GIZ)	https://www.giz.de
United States Agency for International Development (USAID)	https://www.usaid.gov/

Table A3. Search terms.

Concept	Search terms
Hand hygiene	Handwash* OR hygiene OR hand wash* OR with soap OR hand hygiene* OR clean hands OR WASH
Community settings	community OR household OR domestic OR public space OR institution* OR work* OR occupation* OR school OR prison OR market* OR relig* OR education* OR detention*
Guideline	guideline OR guide OR handbook OR manual OR toolkit

For peer review only - http://bmjopen.bmj.com/site/about/guidelines.xhtml

Table A4. Hand Hygiene for All Initiative core partners**.**

United Nations Children's Fund
International Labour Organization
World Bank
United Nations High Commission for Refugees
COVID-19 Hygiene Hub
Sanitation and Water for All
Global Handwashing Partnership
International Federation of Red Cross
WaterAid

Table A5. Data extraction template.

#	Hand hygiene parameter	Guideline title 1 (author, year)		Guideline title 2 (author, year)		Guideline title 3 (author, year)	
		Recommendation	Evidence level[1]	Recommendation	Evidence level	Recommendation	Evidence level
**General**							
1	Community setting(s)						
2	Other guideline specification (e.g., COVID-19)						
**Effective hand hygiene**							
3	Hand hygiene definition						
4	Effective/safe hand hygiene definition						
5	Terminology for defining when to practice hand hygiene						
6	Key moments						
7	Basic handwashing knowledge						
8	Basic hand hygiene knowledge						
**Minimum requirements for effective hand hygiene**							
**Handwashing with soap**							
10	Soap						
11	Soap quantity						
12	Handwashing services						
13	Handwashing with soap duration						

For peer review only

14	Water quality						
15	Water quantity						
16	Alternative water sources for handwashing						
17	Wastewater management						
18	Location of handwashing stations						
19	Spacing and number of users per handwashing station						
**Hand drying**							
20	Hand drying materials						
21	Disposal of hand drying materials						
22	Conditional recommendations						
**Handwashing with soap alternatives**							
23	Alcohol-based hand rub and alcohol content						
24	Hand hygiene duration						
25	Other materials for hand hygiene						
26	Conditional recommendations						
27	Alternatives not recommended						
28	Location of hand hygiene stations						
**Hand hygiene station design considerations**							
29	Design principles						
30	Technical considerations						
31	Accessibility						
32	Local procurement and production						
33	Supply chain						
34	Cost and affordability						
35	Durability						
36	COVID-19 adaptations						
37	Other adaptations						

For peer review only

38	Operation and maintenance – responsibility						
39	Operation and maintenance – actions						
**Behaviour change**							
40	Behaviour change techniques						
41	Content of behaviour change messaging						
42	Behaviour change delivery channel						
43	Person or group tasked with delivering behaviour change intervention						
44	Frequency of behaviour change interventions						
45	Formative research						
46	Behaviour change models or frameworks						
47	Behaviour change approaches						
48	Hand hygiene determinants						
**Government measures – at-scale interventions**							
49	Strengthening markets for hand hygiene products and services						
50	Hygiene promotion practices						
51	At-scale behaviour change interventions						
**Government measures – SWA framework**							
52	Sector policy and strategy						
53	Institutional arrangements						
54	Sector financing						
55	Planning, monitoring, and review						
56	Capacity development						
57	Equity						

For peer review only

[1] One of the five levels of evidence used for the review: 1) systematic review or meta-analysis, 2) randomised controlled trial or controlled trial without randomisation (e.g., quasi-experimental), 3) guideline developed from systematic reviews, 4) observational studies (e.g., cohort, cross-sectional, case-control studies), and 5) expert opinion or anecdotal information (e.g., programme documentation).

Table A6. Excluded documents with reasons.

Author	Guideline title	Year	Reason for exclusion
ICRC	Water, sanitation, and hygiene, and habitat in prisons	2005	Historical version of guideline
World Bank	The Handwashing Handbook: a guide for developing a hygiene promotion program to increase handwashing with soap	2005	Historical version of guideline
IRC	Strengthening Water, Sanitation and Hygiene in schools: a WASH guidance manual with a focus on South Asia	2010	Not a guideline
ICRC	Water, sanitation, and hygiene, and habitat in prisons: supplementary guidance	2012	No recommendation on hand hygiene
GIZ	Field guide: hardware for handwashing in schools	2013	Not a guideline
WEDC	Guidelines for handwashing with soap	2013	Not issued by an international agency
NRC	Water, sanitation and hygiene manual: WASH training for hygiene promotion staff	2015	Not a guideline
ILO	WASH@Work: self-training handbook	2016	Historical version of guideline
WSUP	School hygiene manual	2016	Not for international audience
Rotary	A guide to WASH in schools	2017	Not a guideline
Global Handwashing Partnership	Clean hands for all: a toolkit for hygiene advocacy	2018	Not a guideline
COOPI	WASH handbook for teachers and facilitators	2019	Not for international audience
WASH'Em	How to design handwashing facilities that change behaviour	2019	Does not target a community setting
Africa CDC	Hand washing facility options for resource limited settings	2020	Not a guideline
British Psychological Society	Behavioural science and disease prevention psychological guidance: encouraging hand hygiene in the community	2020	Not a guideline

Coultas et al.	Handwashing compendium for low-resource settings: a living document, 3rd edition	2020	Not a guideline
Global Handwashing Partnership	The Handwashing Handbook	2020	Not a guideline
Smart Centre Group	Smart hygiene solutions: affordable options for household level	2020	Not a guideline
UN Habitat	UN-Habitat guidance on COVID-19 and public spaces	2020	No recommendation on hand hygiene
UNHCR	COVID-19 refugees return to schooling guidelines	2020	No recommendation on hand hygiene
UNICEF et al.	Panduan Cuci Tangan Pakai Sabun	2020	Not in English or French
WaterAid	COVID-19 guidance: prioritising hygiene for workforce health and business resilience	2020	No recommendation on hand hygiene
WaterAid Nepal	Technical brief on contactless handwashing stations	2020	Not a guideline
Bolton, L.	WASH in schools for student return during the COVID-19 pandemic	2021	Not issued by an international agency
ILO	WASH@Work: self-training handbook (revised)	2021	Not a guideline
Government of Vietnam	A guide to integrated hand washing with soap communication	n/d	Not for international audience
WaterAid	Technical manual on community water supply, hygiene and sanitation facilities	n/d	Not a guideline
WaterAid Bangladesh	Handwashing stations: an easy-to-use technological and context-based handwashing stations manual	n/d	Not a guideline

For peer review only

Table A7. List of included guidelines.

Author	Guideline	Year	Community setting	COVID-19 response
UNICEF	A manual on hygiene promotion	1999	Public	No
WHO	WHO recommended strategies for the prevention and control of communicable diseases	2001	Domestic & public	No
WHO	Health villages: a guide for communities and community health workers	2002	Public	No
WHO	Water, sanitation, and hygiene standards for schools in low-cost settings	2009	Institutional	No
ICRC	Water, sanitation, hygiene, and habitat in prisons	2013	Institutional	No
ICRC	Health care in detention	2015	Institutional	No
WHO	Public health for mass gatherings: key considerations	2015	Public	No
SNV	Behaviour change communication guidelines	2016	Public	No
UNICEF, GIZ	Scaling up group handwashing in schools: compendium of group washing facilities across the globe	2016	Schools	No
Malteser	WASH guidelines for field practitioners	2017	Multiple settings	No
Oxfam	Handwashing technical briefing note	2017	Public	No
UNHCR	UNHCR hygiene promotion guidelines	2017	Public	No
WHO	Guidelines on sanitation and health	2018	Domestic & public	No
WHO	Recommendations to Member States to improve hand hygiene practices to help prevent COVID-19	2020	Public	Yes
UNICEF	Handwashing stations and supplies for the COVID-19 response	2020	Domestic & public	Yes
ILO	Hand hygiene at the workplace: an essential occupational safety and health prevention and control measure against COVID-19	2020	Workplace	Yes
GOAL	WASH and IPC measures in households and public spaces	2020	Domestic & public	No
WHO	Considerations for community hand hygiene practices in low-resource situations	2020	Public	No
WHO	Overview of public health and social measures in the context of COVID-19	2020	Public	Yes
GWC	COVID-19 response guidance note - 15 April 2020	2020	Domestic & public	Yes
GWC	Health and hygiene promotion guidance document for COVID-19	2020	Public	Yes
ILO	Safe return to work: guide for employers on COVID-19 prevention	2020	Institutional	Yes
SNV	Practical options for hand-washing stations: a guide for promoters and producers	2020	Multiple settings	No
UNHCR	Technical WASH guidance for COVID-19 preparedness and response	2020	Public	Yes
UNICEF	COVID-19 emergency preparedness and response: WASH and infection prevention and control measures in schools	2020	Schools	Yes
UNICEF	COVID-10 handwashing with soap (HWWS) facilities: compendium of indicative layouts, designs, and cost estimates	2020	Public	Yes

For peer review only - http://bmjopen.bmj.com/site/about/guidelines.xhtml

UNICEF et al.	Handwashing with soap in schools: a technical options manual	2020	Schools	No
UNICEF, WHO, IFRC	Key messages and actions for COVID-19 prevention and control in schools	2020	Schools	Yes
UNICEF	Understanding Hygiene promotion in the context of Risk Communication & Community Engagement (RCCE) and Infection Control and Prevention (IPC) for the COVID-19 outbreak	2020	Domestic & public	Yes
UNICEF	WASH and Infection Prevention and Control (IPC) measures in households and public spaces	2020	Domestic & public	Yes
UNICEF, WHO, IFRC	Interim guidance for COVID-19 prevention and control in schools	2020	Institutional	Yes
WASH4WORK	Hand hygiene protocol for the workplace	2020	Institutional	Yes
Water Mission	Handwashing station program: requirements and recommendations	2020	Public	Yes
WaterAid	Technical guide for handwashing facilities in public spaces and buildings	2020	Public	Yes
WHO	Actions for consideration in the case and protection of vulnerable populations from COVID-19	2020	Vulnerable populations	Yes
WHO	Considerations for public health and social measures in the workplace in the context of COVID-19: Annex to Considerations in adjusting public health and social measures in the context of COVID-19	2020	Workplace	Yes
WHO	Considerations for school-related public health measures in the context of COVID-19: Annex to Considerations in adjusting public health and social measures in the context of COVID-19	2020	Institutional	Yes
WHO	Guidance on COVID-19 for the care of older people and people living in long-term care facilities, other non- acute care facilities and home care	2020	Institutional	Yes
WHO	Interim recommendations on obligatory hand hygiene against transmission of COVID-19	2020	Public spaces	Yes
WHO	Practical considerations and recommendations for religious leaders and faith-based communities in the context of COVID-19	2020	Public spaces	Yes
WHO	Water, sanitation, hygiene, and waste management for SARS-CoV-2, the virus that causes COVID-19: interim guidance	2020	Domestic & public	Yes
CDC	When and how to wash your hands	2021	Public	No
CDC	Hand hygiene at school	2021	Institutional	No
CDC	Hand hygiene at work	2021	Institutional	No
CDC	Interim guidance on management of coronavirus disease 2019 (COVID-19) in correctional and detention facilities	2021	Institutional	Yes
CDC	Interim guidance on people experiencing unsheltered homelessness	2021	Vulnerable populations	Yes
WHO	Key planning recommendations for mass gatherings in the context of COVID-19: interim guidance	2021	Public	Yes

For peer review only - http://bmjopen.bmj.com/site/about/guidelines.xhtml

WHO	Preparedness, prevention, and control of COVID-19 in prisons and other places of detention: interim guidance	2021	Institutional	Yes
WHO, ILO	Preventing and mitigating COVID-19 at work	2021	Institutional	Yes
WHO	Safe Eid al Adha practices in the context of COVID-19: interim guidance	2021	Public	Yes
WHO	Safe Ramadan practices in the context of COVID-19: interim guidance	2021	Public	Yes

For peer review only

Table A8. Number of recommendations per parameter across all community settings.

Parameters	Number of recommendations	Number of guidelines with recommendations
**Effective hand hygiene**		
Guidelines that define hand hygiene	3	3
Guidelines that define safe hand hygiene	2	2
Terminology for defining when to practice safe hand hygiene	38	38
Critical control points	145	38
Basic handwashing knowledge	8	8
Basic hand hygiene knowledge	0	0
**Handwashing with soap & hand drying**		
Handwashing infrastructure	33	24
Handwashing materials	68	46
Amount of soap	7	6
Handwashing services	37	30
Handwashing with soap duration	17	17
Water quality	19	16
Water quantity	9	10
Alternative water sources for handwashing	15	16
Wastewater management	23	13
Location of handwashing stations	59	30
Spacing and number of users per handwashing station	9	8
Hand drying materials	36	19
Disposal of hand drying materials	9	9
Conditional recommendations	7	7
**Handwashing with soap alternatives**		
Alcohol-based hand rub	32	32
Hand rubbing duration	3	3
Other materials for hand hygiene	39	17
Conditional recommendations	29	22
Alternatives not recommended	11	7
Location of hand hygiene stations	21	12
**Hand hygiene station design considerations**		
Design principles	10	6
Technical considerations	5	4
Accessibility	19	14
Local procurement and production	11	9
Supply chain	3	3
Cost and affordability	7	7
Durability	8	6
COVID-19 adaptations	26	16
Other adaptations	17	10
Operation and maintenance – responsibility	8	6
Operation and maintenance – actions	9	8
**Changing behaviour**		
Behaviour change techniques	29	14
Content of behaviour change messaging	27	25
Behaviour change delivery channels	42	25
Person or group tasked with delivery behaviour change intervention	13	10
Frequency of behaviour change interventions	2	2
Formative research	5	4

For peer review only

Behaviour change models or frameworks	15	3
Behaviour change approaches	9	2
Hand hygiene determinants	11	3
**Government interventions**		
Strengthening markets for hand hygiene products and services	2	2
Hygiene promotion practices	0	0
At-scale behaviour change interventions	0	0
Sector policy and strategy	0	0
Institutional arrangements	5	5
Sector financing	0	0
Planning, monitoring, and review	2	2
Capacity development	1	1
Equity	1	1

For peer review only

Table A1. The completed Preferred Reporting Items for Systematic reviews and Meta-Analyses extension for Scoping Reviews (PRISMA-ScR) checklist.

SECTION	ITEM	PRISMA-ScR CHECKLIST ITEM	REPORTED ON PAGE #
**TITLE**			
Title	1	Identify the report as a scoping review.	2
**ABSTRACT**			
Structured summary	2	Provide a structured summary that includes (as applicable): background, objectives, eligibility criteria, sources of evidence, charting methods, results, and conclusions that relate to the review questions and objectives.	3
**INTRODUCTION**			
Rationale	3	Describe the rationale for the review in the context of what is already known. Explain why the review questions/objectives lend themselves to a scoping review approach.	5
Objectives	4	Provide an explicit statement of the questions and objectives being addressed with reference to their key elements (e.g., population or participants, concepts, and context) or other relevant key elements used to conceptualize the review questions and/or objectives.	5
**METHODS**			
Protocol and registration	5	Indicate whether a review protocol exists; state if and where it can be accessed (e.g., a Web address); and if available, provide registration information, including the registration number.	5
Eligibility criteria	6	Specify characteristics of the sources of evidence used as eligibility criteria (e.g., years considered, language, and publication status), and provide a rationale.	6
Information sources	7	Describe all information sources in the search (e.g., databases with dates of coverage and contact with authors to identify additional sources), as well as the date the most recent search was executed.	6
Search	8	Present the full electronic search strategy for at least 1 database, including any limits used, such that it could be repeated.	Supplemental Material (Table A3)
Selection of sources of evidence	9	State the process for selecting sources of evidence (i.e., screening and eligibility) included in the scoping review.	6 – 7
Data charting process	10	Describe the methods of charting data from the included sources of evidence (e.g., calibrated forms or forms that have been tested by the team before their use, and whether data charting was done independently or in duplicate) and any processes for obtaining and confirming data from investigators.	7
Data items	11	List and define all variables for which data were sought and any assumptions and simplifications made.	7 and Supplemental Material (Table A5)
Critical appraisal of individual sources of evidence	12	If done, provide a rationale for conducting a critical appraisal of included sources of evidence; describe the methods used and how this information was used in any data synthesis (if appropriate).	Click here to enter text.7

SECTION	ITEM	PRISMA-ScR CHECKLIST ITEM	REPORTED ON PAGE #
Synthesis of results	13	Describe the methods of handling and summarizing the data that were charted.	7
**RESULTS**			
Selection of sources of evidence	14	Give numbers of sources of evidence screened, assessed for eligibility, and included in the review, with reasons for exclusions at each stage, ideally using a flow diagram.	7 – 8
Characteristics of sources of evidence	15	For each source of evidence, present characteristics for which data were charted and provide the citations.	Supplemental Material (Table A6)
Critical appraisal within sources of evidence	16	If done, present data on critical appraisal of included sources of evidence (see item 12).	15
Results of individual sources of evidence	17	For each included source of evidence, present the relevant data that were charted that relate to the review questions and objectives.	8 – 15
Synthesis of results	18	Summarize and/or present the charting results as they relate to the review questions and objectives.	8 – 15
**DISCUSSION**			
Summary of evidence	19	Summarize the main results (including an overview of concepts, themes, and types of evidence available), link to the review questions and objectives, and consider the relevance to key groups.	15 – 17
Limitations	20	Discuss the limitations of the scoping review process.	17
Conclusions	21	Provide a general interpretation of the results with respect to the review questions and objectives, as well as potential implications and/or next steps.	18
**FUNDING**			
Funding	22	Describe sources of funding for the included sources of evidence, as well as sources of funding for the scoping review. Describe the role of the funders of the scoping review.	19

For peer review only

BMJ Open

Recommendations for hand hygiene in community settings: a scoping review of current international guidelines

Journal:	*BMJ Open*
Manuscript ID	bmjopen-2022-068887.R2
Article Type:	Original research
Date Submitted by the Author:	12-Jan-2023
Complete List of Authors:	MacLeod, Clara; London School of Hygiene and Tropical Medicine Faculty of Infectious and Tropical Diseases, Department of Disease Control<br>Braun, Laura; London School of Hygiene and Tropical Medicine Faculty of Infectious and Tropical Diseases, Department of Disease Control<br>Caruso, Bethany; Emory University, Hubert Department of Global Health, Rollins School of Public Health<br>Chase, Claire; World Bank Group<br>Chidziwisano, Kondwani; Malawi University of Business and Applied Sciences, Department of Environmental Health and WASHTED<br>Chipungu, Jenala; Center for Infectious Disease Research in Zambia<br>Dreibelbis , Robert; London School of Hygiene and Tropical Medicine Faculty of Infectious and Tropical Diseases, Department of Disease Control<br>Ejemot-Nwadiaro, Regina; University of Calabar, Department of Public Health, College of Medical Sciences<br>Gordon, Bruce ; World Health Organization, Water, Sanitation, Hygiene and Health Unit<br>Esteves Mills, Joanna; World Health Organization, Water, Sanitation, Hygiene and Health Unit<br>Cumming, Oliver; London School of Hygiene and Tropical Medicine Faculty of Infectious and Tropical Diseases, Department of Disease Control
**Primary Subject Heading**:	Public health
Secondary Subject Heading:	Global health, Public health
Keywords:	Public health < INFECTIOUS DISEASES, PUBLIC HEALTH, COVID-19

SCHOLARONE™ Manuscripts

I, the Submitting Author has the right to grant and does grant on behalf of all authors of the Work (as defined in the below author licence), an exclusive licence and/or a non-exclusive licence for contributions from authors who are: i) UK Crown employees; ii) where BMJ has agreed a CC-BY licence shall apply, and/or iii) in accordance with the terms applicable for US Federal Government officers or employees acting as part of their official duties; on a worldwide, perpetual, irrevocable, royalty-free basis to BMJ Publishing Group Ltd ("BMJ") its licensees and where the relevant Journal is co-owned by BMJ to the co-owners of the Journal, to publish the Work in this journal and any other BMJ products and to exploit all rights, as set out in our licence.

The Submitting Author accepts and understands that any supply made under these terms is made by BMJ to the Submitting Author unless you are acting as an employee on behalf of your employer or a postgraduate student of an affiliated institution which is paying any applicable article publishing charge ("APC") for Open Access articles. Where the Submitting Author wishes to make the Work available on an Open Access basis (and intends to pay the relevant APC), the terms of reuse of such Open Access shall be governed by a Creative Commons licence – details of these licences and which Creative Commons licence will apply to this Work are set out in our licence referred to above.

Other than as permitted in any relevant BMJ Author's Self Archiving Policies, I confirm this Work has not been accepted for publication elsewhere, is not being considered for publication elsewhere and does not duplicate material already published. I confirm all authors consent to publication of this Work and authorise the granting of this licence.

For peer review only

Title

Recommendations for hand hygiene in community settings: a scoping review of current international guidelines

Authors

Clara MacLeod[1,*], Laura Braun[1], Bethany A. Caruso[2], Claire Chase[3], Kondwani Chidziwisano[4], Jenala Chipungu[5], Robert Dreibelbis[1], Regina Ejemot-Nwadiaro[6], Bruce Gordon[7], Joanna Esteves Mills[7], Oliver Cumming[1]

Affiliations

[1] Department of Disease Control, London School of Hygiene and Tropical Medicine, Keppel St, London, WC1E 7HT, UK
[2] Hubert Department of Global Health, Rollins School of Public Health, Emory University, Atlanta, Georgia,
[3] The World Bank, Washington, DC 20433, United States of America
[4] Department of Environmental Health and WASHTED, Malawi University of Business and Applied Sciences, Blantyre, Malawi
[5] Centre for Infectious Disease Research in Zambia, Lusaka, Zambia
[6] Department of Public Health, College of Medical Sciences, University of Calabar, Calabar, Nigeria
[7] Water, Sanitation, Hygiene and Health Unit, World Health Organization, Avenue Appia 20, 1202 Geneva, Switzerland

* Correspondence to Clara MacLeod: clara.macleod@lshtm.ac.uk

Word count: 5,279

Abstract count: 297

Number of references: 98

For peer review only

ABSTRACT

Background: Hand hygiene is an important measure to prevent disease transmission.

Objective: To summarise current international guideline recommendations for hand hygiene in community settings and to assess to what extent they are consistent and evidence-based.

Eligibility criteria: We included international guidelines with one or more recommendations on hand hygiene in community settings – categorised as domestic, public, or institutional – published by international organisations, in English or French, between 1 January 1990 and 15 November 2021.

Data sources: To identify relevant guidelines, we searched the World Health Organization Institutional Repository for Information Sharing database, Google, websites of international organisations, and contacted expert organisations and individuals.

Charting methods: Recommendations were mapped to four areas related to hand hygiene: i) effective hand hygiene; ii) minimum requirements; iii) behaviour change; and iv) government measures. Recommendations were assessed for consistency, concordance, and whether supported by evidence.

Results: We identified 51 guidelines published between 1999 and 2021 by multilateral agencies and international non-governmental organisations containing 923 recommendations. Handwashing with soap is consistently recommended as the preferred method for hand hygiene across all community settings. Most guidelines specifically recommend handwashing with plain soap and running water for at least 20 seconds; single-use paper towels for hand drying; and alcohol-based hand rub (ABHR) as a complement or alternative to handwashing. There are inconsistent and discordant recommendations for water quality for handwashing, affordable and effective alternatives to soap and ABHR, and the design of handwashing stations. There are gaps in recommendations on soap and water quantity, behaviour change approaches, and government measures required for effective hand hygiene. Less than 10% of recommendations are supported by any cited evidence.

Conclusion: While current international guidelines consistently recommend handwashing with soap across community settings, there remain gaps in recommendations where clear evidence-based guidance might support more effective policy and investment.

For peer review only

Strengths and limitations of this study

- The scoping review follows the Arksey and O'Malley methodological framework to identify, retrieve, and summarise current international guideline recommendations for hand hygiene in community settings.
- Various sources were systematically searched to identify relevant guidelines published by multilateral agencies and international non-governmental organisations between January 1990 and November 2021.
- The search was limited to guidelines published in English or French and therefore may have excluded relevant guidelines published in other languages.
- The quality of the included guidelines was not assessed, although we did consider the extent to which recommendations were based on cited evidence.

For peer review only

INTRODUCTION

Hand hygiene, including handwashing with soap and other methods such as alcohol-based hand rubs (ABHRs), is an important public health measure that can prevent the transmission of a range of diseases.[1] Handwashing with soap has been found to be a cost-effective intervention[2] that can reduce the risk of both diarrhoeal disease and acute respiratory infections (ARIs) by over 20%.[3–9] Handwashing with soap has also been linked to the reduction of certain neglected tropical diseases (NTDs), including trachoma and some soil-transmitted helminth infections.[10,11] Recently, handwashing with soap and the use of ABHRs were advised as one of the key control measures during the COVID-19 pandemic[12,13] and were found to be effective.[14]

This scoping review focuses on hand hygiene in non-healthcare settings, which we collectively refer to as “community settings”. Using the definition set out in the Ottawa Charter, we consider settings as where “health is created and lived by people within the settings of their everyday life; where they learn, work, play and love”,[15] and include domestic, public, and institutional settings. The practice of hand hygiene – and access to the facilities which enable this – is often limited in these community settings, particularly in low- and middle-income countries.[16] In the domestic setting, 30% of the global population does not have access to a basic handwashing facility with soap and water at home,[17,18] with three quarters of those living in low-income countries.[17] In institutional settings, an estimated 43% of schools worldwide do not have access to basic hand hygiene facilities[18] but there is limited data for other institutional settings, such as the workplace and prisons and places of detention, and public settings, such as markets, transportation hubs, and places of worship.[18]

Despite the international recognition of hand hygiene as a critical public health measure, a recent global assessment of government policies, planning, and financing for hygiene found that while the majority of surveyed countries reported having national policies for hand hygiene, less than 10% had sufficient financing to implement them.[19] Various international guidelines with recommendations on hand hygiene for non-healthcare settings exist[13,20–22] but it is unclear whether current international guidelines are comprehensive, consistent, and based on the most rigorous evidence available. This review aims to summarise current international recommendations for hand hygiene in community settings, identify areas of consistency and concordance, and assess whether recommendations are evidence-based.

METHODS

This review follows the six stages of the Arksey and O’Malley methodological framework for scoping reviews.[23–25] Expert consultation (stage 6) was integrated throughout the scoping review process[24] to obtain feedback on the scope and conceptual framework and to identify any additional guidelines beyond those identified during the electronic search. Our review is described according to the Preferred Reporting Items for Systematic reviews and Meta-Analyses extension for Scoping Reviews (PRISMA-ScR)[26] and a PRISMA-ScR checklist is included in the supplementary materials (Table A1). The protocol was pre-registered with OSF Registries.[27]

Identifying the research question (stage 1)

To identify and refine the research question, a conceptual framework was developed for this review and built around three key concepts: i) hand hygiene, ii) non-healthcare settings, and iii) current international guidelines (Figure 1). In this review, we define hand hygiene as any action of hand cleansing for the purpose of removing or deactivating pathogens from hands.[28] Effective hand hygiene is defined as any practice which removes or deactivates pathogens from hands and thereby limits disease transmission.[28]

For peer review only - http://bmjopen.bmj.com/site/about/guidelines.xhtml

The first key concept in the conceptual framework – hand hygiene – covers four areas: effective hand hygiene, minimum requirements, behaviour change, and government measures (Figure 1). Effective hand hygiene refers to definitions and practices. Minimum requirements refer to the materials, services, and infrastructure required for effective hand hygiene. Behaviour change denotes the appropriate behavioural performances that promote and sustain effective hand hygiene. Government measures concern actions taken by governments to ensure effective hand hygiene, which are categorised according to an established framework[29] as follows: policy and strategy; institutional arrangements; sector financing; planning, monitoring, and review; capacity development; and equity.

The second key concept – community settings – is defined as settings where healthcare is not routinely delivered,[28] broadly spanning all places where people "learn, play, work and love"[15] and specifically including domestic, public, and institutional settings. As these settings may not exclusively refer to physical settings,[30] the review also includes recommendations for vulnerable populations (e.g., people experiencing homelessness) who may reside permanently or semi-permanently in public spaces. Nursing homes, long-term care facilities, non-acute care facilities, and home care are also included in the review under institutional settings, as the boundary between healthcare and non-healthcare settings is often unclear or recommendations for these sub-settings may not be disaggregated, especially in the context of COVID-19 where people received care at home.

The third concept – guideline – is defined as a published document where the primary purpose is to provide specific guidance, in the form of recommendations, towards a course of action. A recommendation is defined as a statement designed to assist a targeted actor to make an informed decision on whether, when, and how to undertake a specific action.[31] Our review is limited to international guidelines to identify generalisable recommendations of global relevance.

Identifying relevant studies (stage 2)

To identify relevant guidelines, the search strategy consisted of the following sources: (1) the World Health Organization (WHO) Institutional Repository for Information Sharing (IRIS) database, (2) Google search engine, (3) websites of international organisations known to work on hand hygiene (Table A2), and (4) contacting experts. We searched the WHO IRIS database using pre-specified search terms related to hand hygiene, non-healthcare settings, and guidelines (Table A3). The search in Google was carried out using the anonymous function in the web browser (Chrome) to reduce the influence of the reviewer's (CM) individual search history. Search strings were constructed by using multiple combinations of search terms from Table A3. The first 10 pages of Google were reviewed by one reviewer (CM). Documents that at first appeared related to the research question and met the inclusion criteria were included for further screening. Expert consultations were also conducted with 'Hand Hygiene for All' Initiative core partners (Table A4) to identify potentially relevant guidelines. The reference lists of guidelines were also hand-searched for any additional relevant documents. The search was limited to English and French languages and publication date was restricted to 1 January 1990 onwards to identify current guidelines.

Study selection (stage 3)

Documents meeting the following criteria were included: i) international guideline, ii) offers one or more recommendations on hand hygiene, iii) targets at least one community setting, as defined in the conceptual framework, iv) published by an international non-governmental organisation (NGO), multilateral agency, or public health agency, v) published in English or French, and vi) published between 1 January 1990 and 15 November 2021. The review excludes guidelines for humanitarian settings, as internationally agreed guidance on hand hygiene in humanitarian settings and complex

For peer review only

emergencies is available through the Sphere standards for water, sanitation, and hygiene promotion.[32] Only the most recent versions of guidelines were included, with previous versions of the same guidelines excluded. Country-specific guidelines were also excluded.

All documents retrieved from electronic searches and expert consultations were transferred to Mendeley[33] for de-duplication. Inclusion was completed in two stages: (1) title and abstracts were screened for eligibility by one reviewer (CM); and (2) full texts of all potentially eligible documents were retrieved and independently assessed for inclusion by two reviewers (CM and LB). Disagreement between reviewers on inclusion was resolved through arbitration by a third reviewer (OC).

Charting the data (stage 4)

Guideline characteristics and recommendations from included guidelines were double-extracted by two reviewers (CM and LB) using a standardised data extraction template in MS Excel[34] and then cross-checked for accuracy. As with inclusion, a third reviewer (OC) provided arbitration if agreement on extraction could not be reached. The data extraction form (Table A5) included information on guideline characteristics, such as author, year of publication, target setting, and COVID-19 response, as well as 57 specific parameters related to the four areas of hand hygiene described in the conceptual framework (Figure 1). Recommendations for each parameter were extracted from included guidelines where possible.

Collating, summarising, and reporting the results (stage 5)

Guideline recommendations were first summarised for each parameter across community settings and then disaggregated by domestic, public, or institutional setting where relevant. Definitions and recommendations for hand hygiene were assessed for consistency, concordance, and whether supported by cited evidence. Recommendations were classified as consistent, fairly consistent, or inconsistent if they featured in 10 or more, four to nine, or less than four guidelines, respectively. Concordance is here defined as parameters with no consistent or fairly consistent recommendations at odds with each other. We also used a hierarchical system to classify evidence cited for recommendations into five levels, adapted according to an established classification system:[35] 1) systematic review or meta-analysis, 2) randomised controlled trial or controlled trial without randomisation (e.g., quasi-experimental), 3) guideline (either developed from systematic reviews or not developed from systematic reviews), 4) observational study (e.g., cohort, cross-sectional, case-control studies), and 5) expert opinion or anecdotal information (e.g., programme documentation). A recommendation was considered evidence-based if the guideline provided a specific citation for the recommendation, which was coded according to the level of evidence cited. Finally, evidence gaps were defined as parameters with very few recommendations (i.e., less than 10 recommendations, equivalent to less than 20% of guidelines providing a recommendation).

Patient and public involvement

There was no public or patient involvement in the course of this project.

RESULTS

Search results

Electronic searches were conducted on 15 November 2021, identifying 3,360 records (2,432 from the WHO IRIS database, 900 from Google, 28 from international agency websites) (Table A2) and a further 11 records identified through expert consultation. Following de-duplication, a total of 3,132 records

For peer review only

were screened by title and abstract, and 125 documents were sought for retrieval for full-text screening, with one document not accessible. Finally, 51 guidelines are included in the review (Figure 2). The 73 documents excluded during the full-text review are listed in the Supplemental Material with reasons for exclusion (Table A6).

Description of included guidelines

The 51 included guidelines were published in English between 1999 and 2021, with 38 published in 2020 or later and 31 providing guidance specifically on hand hygiene to help prevent the transmission of the COVID-19 virus. Among the 51 included guidelines, 67% are published by multilateral agencies (WHO, UNICEF, UNHCR, and ILO), 23% by international NGOs, and 10% by the United States Centers for Disease Control and Prevention (US CDC) (Table A7). Most guidelines target public and institutional settings, while none exclusively target the domestic setting. More specifically, 43% (n=22) target the public setting, 43% (n=22) the institutional setting, 4% (n=2) the domestic and public setting, and 10% (n=5) more than one setting (e.g., domestic, public, and/or institutional). Of the 22 guidelines for the public setting, 20 concern public spaces, and two concern vulnerable populations within public spaces (e.g., people experiencing unsheltered homelessness and people living in dense, informal settlements). For institutional settings, eight guidelines concern schools, six the workplace, four prisons and places of detention, three places of worship, and one long-term care facilities and home care. Almost no guidelines, however, define a community setting. The majority of recommendations are generalisable to high-, medium-, and low-income settings. Overall, we extracted 923 recommendations from the 51 included guidelines (Table A8).

Recommendations for effective hand hygiene

Hand hygiene definitions

Only 10% of guidelines provide a clear definition for hand hygiene or safe or effective hand hygiene.[36–40] Meanwhile, 75% of guidelines provide at least one recommendation on when to practice hand hygiene, referred to in our review as a 'key moment'. Guidelines use inconsistent terms for defining when to practice hand hygiene, such as 'key times' (14%),[37,41–46] 'critical times' (8%),[47–50] and 'key moments' (4%).[21,51] Otherwise, guidelines either do not use a specific term for defining a key moment (49%) or do not recommend at least one key moment (25%).

Hand hygiene practices

There is agreement among guidelines on what constitutes effective hand hygiene across community settings, although there are gaps in recommendations on how to practice effective hand hygiene. Almost all guidelines (90%) recommend washing hands with soap and for a duration of at least 20 seconds (27%) (Table 1). In addition, 63% of guidelines recommend the use of ABHR as an alternative or complement to handwashing with soap, though very few guidelines recommend a duration for hand rubbing. There is a lack of recommendations on handwashing or hand rubbing technique (16%),[21,40–42,47,52–54] with two of these guidelines (25%, n=2)[21,40] referring to the WHO instructions for hand hygiene in healthcare settings.[28] Meanwhile, 24% of guidelines recommend the inclusion or provision of simple instructions on handwashing technique in hygiene promotion programmes, yet none of these guidelines include specific steps on handwashing or hand rubbing technique.

Guidelines provide inconsistent recommendations on when to practice hand hygiene. Over 30 different individual key moments are recommended among the guidelines. Despite this inconsistency, the individual key moments most commonly mentioned include 'before and after eating', 'after using

For peer review only

the toilet', 'before and after preparing food', 'after blowing nose, coughing, or sneezing', and 'after touching public surfaces or objects' (Table 1). The latter two feature most commonly among guidelines published during the COVID-19 pandemic. Fairly consistently recommended individual key moments include 'when entering or exiting the home or public space', 'after changing a child's diaper', and 'when hands are visibly dirty'. Almost all guidelines recommend clusters of key moments (i.e., individual key moments recommended together). The most common ones are 'after using the toilet' with 'before and after eating' (39%, n=20); 'after using the toilet' with 'before and after preparing food' (24%, n=12); and 'after using the toilet', with 'before and after eating', and 'after blowing nose, coughing, or sneezing' (22%, n=11).

Recommendations for minimum requirements

Handwashing materials

Guidelines consistently recommend plain soap for handwashing across all community settings and paper towels or electric dryers for hand drying in public and institutional settings. Most guidelines (65%) do not recommend a specific type of soap for handwashing, though some specifically recommend bar soap (24%), liquid soap (22%), or "soapy water" (e.g., home-made mixture of powder or liquid soap, or bar soap shavings diluted in water) (16%) (Table 1). No guidelines specifically recommend antibacterial soap. For hand drying after handwashing, most guidelines recommend clean, single use paper towels (39%) and electric air-drying systems (22%) in public and institutional settings. 8% of guidelines recommend a bin with disposal liners and lid for the waste management of paper towels in public and institutional settings.[21,55–57] No guidelines provide a recommendation for hand drying materials in the domestic setting.

Soap and water requirements for handwashing

While guidelines consistently recommend running water for handwashing with soap, there are gaps for soap and water quantity and discordant recommendations on water quality for handwashing. For water services, almost half (43%) of guidelines recommend running water, 16% a piped water system connected to a tap, and 14% water trucking, storage, or manual refilling. Few guidelines recommend a minimum quantity of liquid or bar soap (14%)[43,48,58–61] or water (18%)[12,20,47,50,60–62] required for handwashing (e.g., minimum amount of water needed to wet hands or soap product necessary to cover all surfaces of hands for handwashing with soap) (Table 2). One guideline, for example, recommends less than 5 ml of liquid soap per person per handwashing event,[60] whereas three other guidelines recommend at least 250 grams of bar soap per person per month for handwashing.[43,48,61] For water quality, 16% of guidelines state that water must be of drinking water quality, in line with WHO guidelines.[21,40,41,52,54,63–66] In contrast, 8% state that it does not have to be of drinking water quality,[12,20,39,67] though none specify a quantitative standard nor whether non-drinking quality water may conditionally be used if high quality water is not available. In addition, 10% of guidelines recommend that free residual chlorine must be greater than or equal to 0.5 mg/l after at least 30 minutes of contact time.[43,48,63,68,69]

For peer review only

Table 1. Consistent and fairly consistent recommendations for hand hygiene in community settings.

Parameter	Recommendation	All guidelines (n= 51)	Domestic setting (n= 2)	Public setting (n= 24)	Institutional setting (n= 22)	Multiple settings (n= 5)
**Effective hand hygiene**						
Key moments	Before and after eating	45% (23)	0% (0)	38% (9)	27% (14)	0% (0)
	After using the toilet	41% (21)	0% (0)	38% (9)	55% (12)	0% (0)
	Before and after preparing food	27% (14)	0% (0)	38% (9)	18% (4)	20% (1)
	After blowing nose, coughing, or sneezing	24% (12)	0% (0)	21% (5)	27% (6)	20% (1)
	After touching public surfaces or objects	24% (12)	0% (0)	12% (6)	18% (4)	40% (2)
	When entering and exiting buildings or home	14% (7)	0% (0)	17% (4)	5% (1)	40% (2)
	After changing a child's diaper	12% (6)	0% (0)	17% (4)	0% (0)	40% (2)
	When hands visibly dirty	10% (5)	0% (0)	13% (3)	9% (2)	0% (0)
	Before and after work	8% (4)	0% (0)	0% (0)	18% (4)	0% (0)
	After contact with animals	8% (4)	0% (0)	13% (3)	5% (1)	0% (0)
	Taking care of sick person	8% (4)	0% (0)	13% (3)	5% (1)	0% (0)
Handwashing duration	At least 20 seconds	27% (14)	0% (0)	21% (5)	36% (8)	20% (1)
**Minimum requirements for effective hand hygiene**						
**Materials for effective hand hygiene**						
Soap	Soap	65% (33)	100% (2)	50% (12)	82% (18)	20% (1)
	Bar soap	24% (12)	0% (0)	14% (7)	14% (3)	40% (2)
	Liquid soap	22% (11)	0% (0)	14% (7)	14% (3)	20% (1)
	Soap water	16% (8)	0% (0)	17% (4)	5% (1)	60% (3)
ABHR	ABHR with at least 60% alcohol	35% (18)	0% (0)	16% (8)	45% (10)	0% (0)
	ABHR, no alcohol percentage	22% (11)	50% (1)	17% (4)	23% (5)	20% (1)
Hand drying	Clean, single use paper towels	39% (20)	0% (0)	14% (7)	59% (13)	0% (0)
	Air-drying system	22% (11)	0% (0)	17% (4)	14% (7)	0% (0)
	Bin with disposable liners and lid	8% (4)	0% (0)	4% (1)	14% (3)	0% (0)
**Alternative materials**						
Other materials	Ash	22% (11)	0% (0)	12% (6)	14% (3)	40% (2)
Conditional recommendations	ABHR where soap and water not available	18% (9)	0% (0)	4% (1)	36% (8)	0% (0)
	Ash and water if ABHR or soap not available	14% (7)	0% (0)	13% (3)	14% (3)	20% (1)
	ABHR if hands not visibly soiled	10% (5)	0% (0)	8% (2)	14% (3)	0% (0)
**Water requirements**						
Water services	Running water	43% (22)	0% (0)	16% (8)	55% (12)	40% (2)

For peer review only

	Piped water system connected to tap	16% (8)	0% (0)	13% (3)	18% (4)	20% (1)
	Water trucking, storage, or manual refilling	14% (7)	0% (0)	13% (3)	14% (3)	20% (1)
Other water sources	Rainwater	8% (4)	0% (0)	17% (4)	0% (0)	0% (0)
Wastewater management	Drainage system	16% (8)	0% (0)	8% (2)	23% (5)	20% (1)
	Covered soakaway pit	12% (6)	0% (0)	21% (5)	0% (0)	20% (1)
**Hand hygiene stations**						
Handwashing station	Washbasin	16% (8)	0% (0)	13% (3)	18% (4)	20% (1)
	Bucket or container connected to tap	14% (7)	50% (1)	17% (4)	5% (1)	40% (2)
	Tippy tap	12% (6)	0% (0)	4% (1)	14% (3)	40% (2)
Location of handwashing stations	Key entry/exit points	27% (14)	0% (0)	50% (12)	5% (1)	20% (1)
	Close proximity to toilets	27% (14)	0% (0)	21% (5)	14% (7)	40% (2)
	Food preparation and eating areas	18% (9)	0% (0)	8% (2)	27% (6)	20% (1)
Accessibility	Accessible for all users and vulnerable groups	12% (6)	0% (0)	13% (3)	5% (1)	40% (2)
	Height of soap and water taps appropriate for all	10% (5)	0% (0)	13% (3)	5% (1)	20% (1)
	Age-appropriate handwashing stations	8% (4)	0% (0)	0% (0)	18% (4)	0% (0)
Design considerations	Theft resistance	16% (8)	0% (0)	17% (4)	14% (3)	20% (1)
	Water-saving designs/technologies	12% (6)	0% (0)	13% (3)	9% (2)	20% (1)
COVID-19 adaptations	Taps that limit cross-contamination	16% (8)	0% (0)	14% (7)	0% (0)	20% (1)
	1-2m spacing between handwashing stations	12% (6)	0% (0)	17% (4)	5% (1)	20% (1)
	Disinfect taps regularly	10% (5)	0% (0)	17% (4)	0% (0)	20% (1)
	Towels for opening and closing taps	8% (4)	0% (0)	13% (3)	5% (1)	0% (0)
Availability of materials	Locally available materials for handwashing	10% (5)	0% (0)	8% (2)	9% (2)	20% (1)
**Behaviour change**						
Behaviour change techniques[1]	Prompts and cues	12% (6)	0% (0)	13% (3)	14% (3)	0% (0)
	Habit formation	8% (4)	0% (0)	13% (3)	5% (1)	0% (0)
Content of behaviour change messaging	Doable instructions with proper steps	24% (12)	0% (0)	12% (6)	27% (6)	0% (0)
	Messages that target motivation	10% (5)	0% (0)	8% (2)	14% (3)	0% (0)
Delivery channels	Visual reminders	31% (16)	0% (0)	12% (6)	36% (8)	40% (2)
	Mass communication	24% (12)	0% (0)	14% (7)	9% (2)	60% (3)
	Small group activities	18% (9)	0% (0)	12% (6)	9% (2)	20% (1)
	Interpersonal communication	10% (5)	0% (0)	17% (4)	0% (0)	20% (1)
Formative research	Identify determinants of target behaviour	8% (4)	0% (0)	4% (1)	0% (0)	60% (3)

[1] Using a typology of behaviour change techniques developed by Michie et al. (2013)[70]

For peer review only - http://bmjopen.bmj.com/site/about/guidelines.xhtml

Table 2. Parameters with gaps in recommendations (i.e., fewer than 10 recommendations, equivalent to less than 20% of guidelines providing a recommendation).

Parameters with gaps in recommendations[1]	Percentage (%) of guidelines that provide a recommendation (n)
**Effective hand hygiene**	
Definition of hand hygiene	4% (2)
Definition of safe/effective hand hygiene	6% (3)
Handwashing knowledge	16% (8)
Hand rubbing duration	6% (3)
**Minimum requirements for effective hand hygiene**	
**Soap and water requirements for handwashing**	
Quantity of soap	14% (7)
Quantity of water	18% (9)
**Hand hygiene stations**	
Supply chain for products and materials	6% (3)
Functionality	10% (5)
Cost and affordability	14% (7)
Durability	16% (8)
Operation and maintenance – responsibility	16% (8)
Operation and maintenance – actions	18% (9)
Spacing and number of users per handwashing station	18% (9)
**Behaviour and behaviour change**	
Frequency of behaviour change interventions	4% (2)
Behaviour change approaches	18% (9)
**Government measures**	
Sector policy and strategy	0% (0)
Sector financing	0% (0)
Capacity development	2% (1)
Equity	2% (1)
Planning, monitoring, and review	4% (2)
Institutional arrangements	10% (5)

[1] Parameters with less than 20% of guidelines that provide a recommendation

Alternative materials for hand hygiene

There are consistent recommendations for ABHR as an alternative material for hand hygiene. Of the guidelines that recommend ABHR, 35% (n=18) recommend ABHR with at least 60% alcohol, 22% (n=11) do not specify an alcohol percentage, and only 6% (n=3) recommend an alcohol percentage of at least 70%. In addition, 18% of guidelines recommend ABHR where soap and water are not available[37–39,41,42,52–54,71] and 10% (n=5) only if hands are not visibly soiled.[21,36,40,65,72]

There are discordant recommendations for the use of ash. 22% of guidelines recommend ash as an alternative material to soap for handwashing.[12,36,74,43,45,47,52,64,65,72,73] In addition, 14% recommend ash if ABHR or soap are not available.[12,43,45,47,52,64,74] However, 6% (n=3) of guidelines advise against the use of ash or other products, such as soil, sand, mud, or water alone.[45,47,62] In addition, while some guidelines (8%) recommend 0.05% chlorine solution,[48,53,54,63] one guideline advises against it.[13]

When disaggregated by setting, ABHR is most consistently recommended in public and institutional settings where it may meet larger and more frequent demand than handwashing stations. Similarly, recommendations for the conditional use of ABHR where soap and water are not available feature the most in the institutional setting, particularly in the workplace and schools. Ash is most commonly

For peer review only

recommended in the public setting, which includes low-resource and water-scarce settings where soap and water may not be available.

Hand hygiene stations

Overall, there are inconsistent recommendations on hand hygiene facilities and their location, as well as gaps. Across all settings, guidelines recommend washbasins (e.g., ceramic, cement, or plastic) (16%), a bucket or container connected to tap (14%), and tippy taps (12%) for handwashing stations (Table 1). Guidelines specify 16 different locations for handwashing stations, which include by the entrance and exit of public spaces and buildings (e.g., restaurants, shops, markets, places of worship, train and bus stations) (27%), in close proximity to toilets (27%), and next to food preparation and eating areas (18%). Guidelines also mention placing hand hygiene stations, such as ABHR dispensers, at key entry and exit points of public spaces and buildings (14%). There are inconsistent recommendations on the optimal spacing and number of users per handwashing station, whereas none for ABHR dispensers. Gaps in recommendations include those for hand hygiene materials and product supply chains, cost and affordability, functionality, durability, and hand hygiene station operation and maintenance responsibilities and actions (Table 2).

Recommendations on the location of handwashing stations vary slightly by setting. Close proximity to toilets is consistently recommended for both public and institutional settings (10% and 14%, respectively). By the entrance and exit of public spaces is most consistently recommended for public settings (24%). Next to food preparation and eating areas is most consistently recommended for institutional settings (12%), such as schools and the workplace.

Hand hygiene station access and adaptations

Guidelines recommend hand hygiene stations that are accessible for all and adapted for pandemic response. For example, 10% of guidelines recommend that the height of soap and water taps be appropriate for access by children, the elderly, and disabled (e.g., 500-700 mm basin height for children and 850 mm basin height for wheelchair access) (Table 1). 45% (n=23) of guidelines recommend COVID-19-related adaptations for hand hygiene stations, such as taps that limit cross contamination (16%), one to two meters spacing between stations (12%), regular tap disinfection (10%), and towels for opening and closing taps (8%). Other adaptations include theft resistance (e.g., attaching soap or other movable pieces to the station) (16%) and water-saving designs (e.g., low-flow faucets) (12%).

Recommendations for behaviour change

Overall, there are few recommendations related to hand hygiene behaviour change, though there are consistent recommendations on behaviour change messaging and delivery channels. For behaviour change messaging, some guidelines (10%) recommend messages that target “motives”[75] (Table 1).[20,45,54,64,72] In terms of delivery channels, 31% of guidelines recommend using visual reminders (e.g., signs, posters, or leaflets) and 24% mass communication (e.g., radio, social media, or mobile phone text messaging) to deliver behaviour change messages. Prompts, cues and habit formation are fairly consistently recommended as behaviour change techniques.[20,39,52,67,70,72,76–78] Formative research for behaviour change programmes is recommended by 10% (n=5) of guidelines,[46,47,49,73,76] though only 8% (n=4) specifically mention undertaking formative research to identify behavioural determinants among the target population.[46,47,49,73] Guidelines provide inconsistent recommendations on behaviour change approaches (18%), determinants of hand hygiene to target for interventions (22%), and behaviour change models or frameworks (33%). Lastly, there are gaps in recommendations on frequency of

For peer review only

behaviour change interventions (i.e., how often an intervention should be delivered) and behaviour change approaches (4%)[20,46] (Table 2).

Recommendations for government measures

Overall, few guidelines (22%, n=11) provide a recommendation on government measures. Nonetheless, recommendations for sector policy and strategy include promoting local soap production and fostering public-private partnerships for handwashing (4%).[47,49] For institutional arrangements, 4% of guidelines suggest identifying ways of cross-sectoral collaboration for hand hygiene,[20,49] while other recommendations centre on engaging communities, the private sector, and civil society for the delivery of water, sanitation, and hygiene (WASH) services.[12,65] On planning, monitoring, and review, 4% of guidelines recommend supporting or reinforcing existing monitoring systems or creating a government-led national monitoring system, in line with global hygiene indicators.[49,68]

Evidence-based recommendations

Of the 923 recommendations extracted from the 51 included guidelines, most (93%) do not have a citation for one of the five levels of evidence identified for the review (i.e., systematic review, randomised and quasi-randomised trial, guideline, observational study, or anecdotal information) (Figure 3). Of the remaining 7% of recommendations, less than 1% cite evidence from a systematic review.[46] In addition, less than 1% cite evidence from a randomised or quasi-randomised trial,[12,36] while over 1% cite evidence from observational studies, which are mainly recommendations for alternative hand hygiene materials, such as ash, sand, and soil, or alternative water sources for handwashing, such as cooking or laundry water, bathwater, and seawater.[12,36,47] Less than 1% of recommendations cite other guidelines developed from systematic reviews,[21,47,67,71] while 2.5% cite other guidelines not developed from systematic reviews, [51,56,57,65,72,78,79] such as WHO interim guidelines for hand hygiene in the context of COVID-19.[51,56,57,65,72,78,79] The other 2% of recommendations cite anecdotal information, such as programme documentation.[45,47,62,76]

DISCUSSION

We identified 51 guidelines published between 1999 and 2021 by various international agencies covering a range of community settings. Most guidelines target the public and institutional setting, while surprisingly none exclusively target the domestic setting. Overall, community settings are not clearly defined among the guidelines, which presents an opportunity for future normative guidelines to establish a clear and common definition, especially as it relates to hand hygiene. Overall, no guidelines comprehensively address hand hygiene across domestic, public, and institutional settings, and very few recommendations are evidence-based, highlighting a gap in global normative guidance on hand hygiene in community settings.

Effective hand hygiene

There is agreement among guidelines on what constitutes effective hand hygiene, however, inconsistencies and gaps remain as to when and how to practice hand hygiene. Current recommendations for handwashing with soap reflect findings from published literature, which show that handwashing with soap is an effective means for preventing the transmission of a range of diseases.[3–9] Regarding when to practice hand hygiene, future guidelines could focus key moments on those most likely to interrupt the transmission of infectious diseases in domestic, public, and institutional settings. These might include “after using the toilet” to reduce the transmission of

For peer review only

diarrhoea-causing pathogens,[9] and "after touching high-contact surfaces" or "after coughing or sneezing" to reduce the risk of respiratory infections.[3] Lastly, gaps in recommendations on handwashing and hand rubbing technique present an opportunity for future guidelines to recommend the optimal technique for reducing bacterial load on hands.

Minimum requirements

Consistent recommendations for the use of soap and water and ABHR suggest the widespread acceptability of these materials for effective hand hygiene across community settings. Current recommendations for the use of plain soap are consistent with findings from a systematic review that suggest that plain soap is more effective than antibacterial soap at removing or inactivating pathogens on hands in community settings.[80] Recommendations for running water are equally relevant as unreliable water supply negatively affects households' ability to perform hand hygiene.[81] Gaps in recommendations on minimum quantities of soap and water required for effective hand hygiene are also important for future guidelines to address. The recommended use of ABHR with at least 60% alcohol is in line with several studies which have found that ABHRs with an alcohol concentration between 60 and 80% are more effective at killing germs than those with a lower alcohol percentage, particularly in clinical settings.[82,83] Although ABHR can still inactivate many types of microbes when used correctly, evidence suggests that soap and running water are still more effective at removing certain types of pathogens that may be present on hands.[84] The WHO Guidelines on Hand Hygiene in Health Care, for example, recommend ABHR as the preferred method for routine hand hygiene in healthcare settings when hands are not visibly soiled, as it enables more frequent hand hygiene.[28] Similarly, in community settings, ABHR may be suitable in contexts where frequent hand hygiene is necessary, such as transport hubs and entrances or exits to public spaces and buildings.[85] ABHR may also be favourable where frequent hand hygiene is required, as the repeated use of soap can result in skin dryness and cause chronic irritant contact dermatitis.[28] However, in certain domestic, public, and institutional settings, such as the household or schools, where hands may become more soiled, ABHR may be less likely to effectively inactivate microbes.[82,83] As per current international guideline recommendations, handwashing with soap may therefore be prioritised in these settings, with ABHR as a suitable complement or alternative where frequent hand hygiene is required. Finally, because the transmission of germs is more likely to occur to and from wet hands, hand drying is an essential component of effective hand hygiene, especially for handwashing.[86] Current recommendations for hand drying in public and institutional settings are consistent with those in the WHO Guidelines on Hand Hygiene in Health Care, which recommend that hands should ideally be dried with individual paper towels, otherwise with air dryers.[28] While there is mixed evidence for the most effective hand drying method,[87] the WHO recommendations are based on findings that suggest that paper towels may effectively prevent re-contamination of hands, while also lowering the risk of spreading pathogens through the air compared to electric air dryers.[86]

Discordant recommendations for minimum requirements suggest the need to leverage further research to determine the optimal water quality for handwashing and effectiveness of alternative materials for hand hygiene where soap and ABHR are not widely available. Limited evidence suggests that the use of non-potable water with low-to-moderate levels of *E. coli* contamination may still be effective for handwashing,[88] which may be promising for areas where it is difficult to regularly treat water or where there is intermittent water supply that is prone to contamination. Similarly, two studies found that drinkable water may not be needed for handwashing with soap.[89,90] Nevertheless, the WHO Guidelines on Hand Hygiene in Health Care recommend washing hands with clean, running water whenever possible.[28] While there are discordant recommendations for the use of ash, there is uncertain evidence whether this stops or reduces the spread of pathogens compared to hand cleansing with soap, mud, soil, or no hand cleansing.[91] Future guidelines may consider the efficacy of hand hygiene products along with their availability and acceptability in domestic, public,

For peer review only

and institutional settings to make relevant recommendations, particularly in water-scarce regions or settings where there is limited access to soap or ABHR. For example, soapy water may be a promising low-cost and effective alternative to bar soap in settings where bar or liquid soap is unaffordable.[92] In addition, one interim guideline on hand hygiene practices in low-resource settings, for example, recommends the use of friction-generating materials where clean, running water, soap, or ABHR are not available.[36]

Current inconsistent recommendations for hand hygiene facilities and their placement may limit the practice of effective hand hygiene. Sustaining hand hygiene behaviour change requires consistent access to functional hand hygiene stations at key locations[93,94] and diverse infrastructure is recommended with varying costs. Nonetheless, the appropriateness of these recommendations is likely to depend on the local availability and affordability of materials. One guideline, for example, provides technical recommendations for permanent and semi-permanent handwashing facilities in public places and buildings, focusing on the sustainability and equitable access of these facilities.[20] Similarly, future guidelines may prioritise the accessibility, affordability, and sustainability of materials and infrastructure for hand hygiene across all community settings to address current inconsistent and discordant recommendations. The accessibility and sustainability of hand hygiene stations are particularly important to ensure that they are inclusive and kept functional and well-stocked beyond their installation.[20]

Behaviour change

The gaps in recommendations related to behaviour change suggest the need for guidance based on established behavioural theory and existing evidence. There are some recommendations for behaviour change, though without clear steps on how to develop, implement, and sustain hand hygiene behaviour change interventions. Future guidelines may benefit from leveraging well-established behavioural frameworks and theories[95–97] to make recommendations as to how to develop effective, locally-appropriate strategies beyond information-focused communication. In addition, while most behaviour change theories and frameworks recommended amongst the guidelines note the importance of formative research, very few guidelines recommend undertaking formative research. Yet, formative research plays a key role in adapting hand hygiene behaviour change programmes to high-risk populations and target settings.[98]

Government measures

The significant gaps in recommendations on government measures underscore the current lack of normative standards to guide national governments on the planning, delivery, financing, and monitoring of effective hand hygiene. Indicators also suggest inadequate planning and insufficient funding for hand hygiene among national governments globally.[19] Future guidelines may therefore consider prioritising government measures to support countries in responding to and preventing public health crises, such as the COVID-19 pandemic. Future guidelines may also focus recommendations on hand hygiene monitoring and reporting to improve comparison of hand hygiene indicators within and between countries.

Strengths and limitations

This review has four main limitations. First, as a scoping review, we did not systematically assess the quality of the included guidelines, although we did, for example, consider aspects such as the extent to which recommendations were based on cited evidence. In addition, included guidelines covered WASH and public health topics beyond hand hygiene, so it was thus not necessarily relevant to assess the whole guidelines for quality, but rather focus on the robustness of the specific recommendations

For peer review only

for hand hygiene. Second, the search was limited to guidelines published in English or French and therefore may have excluded relevant guidelines published in other languages. Third, the lack of recommendations for government measures among current international guidelines may not reflect an absolute absence of guidance in this area. Guidance to governments on how to improve uptake of hand hygiene practices in community settings may be included in other sector documents or policy instruments. Fourth, while recommendations were summarised across domestic, public, and institutional settings, there were often too few recommendations for each setting to assess consistency and concordance. Still, with 51 guidelines providing over 900 recommendations for hand hygiene in community settings, findings from this review highlight significant gaps and inconsistencies across community settings that future guidelines may seek to prioritise.

CONCLUSION

This review identified 51 current international guidelines providing 923 recommendations for hand hygiene in community settings. Nonetheless, there are several important areas of discordance and significant gaps in the recommendations among these guidelines. Furthermore, very few recommendations are supported by any qualifying evidence. The COVID-19 pandemic led to numerous national, regional, and international efforts to improve effective hand hygiene in domestic, public, and institutional settings, such as households, public spaces, workplaces, and schools, but the lack of clear recommendations supported by cited evidence may limit progress in this important area of public health.

For peer review only

Figure legends

Figure 1. Conceptual framework for hand hygiene in community settings.
Figure 2. PRISMA flow diagram.
Figure 3. Percentage of recommendations cited by one of the five types of evidence or no evidence.

For peer review only

Contributors

CM, LB, BC, CC, KC, JC, RD, REN, BG, JEM, and OC informed the study protocol. CM carried out the database and grey literature search with input from OC and JEM. JEM, OC, BG, and CM carried out expert consultations. CM and LB screened the retrieved articles for inclusion and extracted the data with input from OC. CM led on data analysis and CM, LB, OC, and JEM led on the presentation of results with inputs from co-authors. CM, LB, JEM, and OC led on writing the manuscript with input from all co-authors. OC, JEM, and BG provided overall supervision, leadership, and advice. The first author (CM) accepts responsibility for the work.

Funding

This research was funded by the World Health Organization and the UK Foreign, Commonwealth, and Development Office.

Competing interests

The authors declare no competing interests.

Patient consent for publication

Not applicable.

Ethics approval

This research did not require institutional review board approval as the data were publicly available and collected from existing online databases and search engines. This research did not involve any human subjects.

Data availability statement

Data are available upon reasonable request.

For peer review only

References

1 Burton M, Cobb E, Donachie P, *et al.* The effect of handwashing with water or soap on bacterial contamination of hands. *Int J Environ Res Public Health* 2011;**8**:97–104. doi:10.3390/ijerph8010097

2 Cairncross S, Valdamis V. Water Supply, Sanitation, and Hygiene Promotion. In: *Disease Control Priorities in Developing Countries*. Washington: : World Bank 2006. 771–792.

3 Freeman M, Stocks M, Cumming O, *et al.* Hygiene and health: systematic review of handwashing practices worldwide and update of health effects. *Trop Med Int Health* 2014;**19**:906–16. doi:10.1111/TMI.12339

4 Rabie T, Curtis V. Handwashing and risk of respiratory infections: A quantitative systematic review. *Trop Med Int Heal* 2006;**11**:258–67. doi:10.1111/j.1365-3156.2006.01568.x

5 Aiello AE, Coulborn RM, Perez V, *et al.* Effect of hand hygiene on infectious disease risk in the community setting: A meta-analysis. *Am J Public Health* 2008;**98**:1372–81. doi:10.2105/AJPH.2007.124610

6 Prüss-Ustün A, Wolf J, Bartram J, *et al.* Burden of disease from inadequate water, sanitation and hygiene for selected adverse health outcomes: An updated analysis with a focus on lowand middle-income countries. *Int J Hyg Environ Health* 2019;**222**:765–77.

7 Mbakaya BC, Lee PH, Lee RLT. Hand hygiene intervention strategies to reduce diarrhoea and respiratory infections among schoolchildren in developing countries: A systematic review. *Int J Environ Res Public Health* 2017;**14**:1–14. doi:10.3390/ijerph14040371

8 Wolf J, Hubbard S, Brauer M, *et al.* Effectiveness of interventions to improve drinking water, sanitation, and handwashing with soap on risk of diarrhoeal disease in children in low-income and middle-income settings: a systematic review and meta-analysis. *Lancet* 2022;**400**:48–59. doi:10.1016/S0140-6736(22)00937-0

9 Ejemot-Nwadiaro R, Ehiri J, Arikpo D, *et al.* Hand washing promotion for preventing diarrhoea (review). *Cochrane Database Syst Rev* 2015;**2015**. doi:10.1002/14651858.CD004265.pub3

10 Stocks ME, Ogden S, Haddad D, *et al.* Effect of Water, Sanitation, and Hygiene on the Prevention of Trachoma: A Systematic Review and Meta-Analysis. *PLoS Med* 2014;**11**. doi:10.1371/journal.pmed.1001605

11 Strunz EC, Addiss DG, Stocks ME, *et al.* Water, Sanitation, Hygiene, and Soil-Transmitted Helminth Infection: A Systematic Review and Meta-Analysis. *PLoS Med* 2014;**11**. doi:10.1371/journal.pmed.1001620

12 WHO. Water, sanitation, hygiene, and waste management for SARS-CoV-2, the virus that causes COVID-19. Geneva, Switzerland: 2020. https://www.who.int/publications/i/item/water-sanitation-hygiene-and-waste-management-for-the-covid-19-virus-interim-guidance

13 WHO. Recommendations to member states to improve hand hygiene practices to help prevent the transmission of the COVID-19 virus. Geneva, Switzerland: 2020.

14 Talic S, Shah S, Wild H, *et al.* Effectiveness of public health measures in reducing the incidence of covid-19, SARS-CoV-2 transmission, and covid-19 mortality: systematic review and meta-analysis. *BMJ* 2021;:375. doi:https://doi.org/10.1136/bmj-2021-068302

15 WHO. The Ottawa Charter for Health Promotion. 1986.http://www.who.int/healthpromotion/conferences/previous/ottawa/en/index.html.

16 Brauer M, Zhao J, Bennitt F, *et al.* Global Access to Handwashing: Implications for COVID-19 Control in Low-Income Countries. *Environ Health Perspect* 2020;**128**. doi:https://doi.org/ 10.1289/EHP7200

17 WHO, UNICEF. Progress on household drinking water, sanitation and hygiene 2000-2020: five years into the SDGs. Geneva, Switzerland: 2020.

18 WHO, UNICEF. Progress on drinking water, sanitation and hygiene in schools: special focus on Covid-19. 2020.

For peer review only

19 WHO. Hygiene - UN-Water GLAAS findings on national policies, plans, targets and finance. World Heal. Organ. 2020.https://www.who.int/water_sanitation_health/publications/hygiene-glaas-findings/en/%0Ahttps://www.unwater.org/publications/hygiene-un-water-glaas-findings-on-national-policies-plans-targets-and-finance/
20 WaterAid. Technical Guide for handwashing facilities in public places and buildings. 2020. https://washmatters.wateraid.org/sites/g/files/jkxoof256/files/technical-guide-for-handwashing-facilities-in-public-places-and-buildings.pdf
21 ILO. Hand hygiene at the workplace: an essential occupational safety and health prevention and control measure against COVID-19. Geneva, Switzerland: 2020.
22 ICRC. Water, Sanitation, and Hygiene and Habitat in Prisons: Supplementary Guidance. Geneva, Switzerland: 2012. https://www.icrc.org/eng/assets/files/publications/icrc-002-4083.pdf
23 Arksey H, O'Malley L. Scoping studies: Towards a methodological framework. *Int J Soc Res Methodol* 2005;**8**:19–32. doi:10.1080/1364557032000119616
24 Levac D, Colquhoun H, O'Brien K. Scoping studies: advancing the methodology. *Implement Sci* 2010;**5**. doi:10.1017/cbo9780511814563.003
25 Westphaln KK, Regoeczi W, Masotya M, *et al.* From Arksey and O'Malley and Beyond: Customizations to enhance a team-based, mixed approach to scoping review methodology. *MethodsX* 2021;**8**:101375. doi:10.1016/j.mex.2021.101375
26 Tricco A, Lillie E, Zarin W, *et al.* PRISMA Extension for Scoping Reviews (PRISMA-ScR): Checklist and Explanation. *Ann Intern Med* 2018;**169**:467–73. doi:10.7326/M18-0850
27 MacLeod C, Braun L, Chipungu J, *et al.* Guideline recommendations for hand hygiene in community settings: scoping review protocol. 2021.https://osf.io/z2gvs/?view_only=95516bea2202437d8fbea11be17fcae7
28 WHO. WHO Guidelines on Hand Hygiene in Health Care. Geneva, Switzerland: 2009.
29 Sanitation and Water for All. Building Blocks. 2022.https://www.sanitationandwaterforall.org/about/our-work/priority-areas/building-blocks (accessed 15 Jun 2022).
30 MacQueen K, McLellan E, Metzger D, *et al.* What is Community? An Evidence-Based Definition for Participatory Public Health. *Am J Public Health* 2001;**91**.
31 WHO. WHO Guidelines. 2022.https://www.who.int/publications/who-guidelines#:~:text=A WHO guideline is defined,practice or public health policy. (accessed 15 Jun 2022).
32 Sphere Association. The Sphere Handbook: Humanitarian Charter and Minimum Standards in Humanitarian Response (fourth edition). 2018.www.spherestandards.org/handbook
33 Elsevier. Mendeley.
34 Microsoft. Microsoft Excel Spreadsheet Software.
35 Gugiu PC, Gugiu MR. A critical appraisal of standard guidelines for grading levels of evidence. *Eval Heal Prof* 2010;**33**:233–55. doi:10.1177/0163278710373980
36 WHO. Considerations for community hand hygiene practices in low-resource situations. 2020. http://iris.wpro.who.int/handle/10665.1/14533
37 CDC. Hand Hygiene at Work. 2021.https://www.cdc.gov/handwashing/handwashing-corporate.html (accessed 1 Nov 2021).
38 CDC. Interim Guidance on Management of Coronavirus Disease 2019 (COVID-19) in Correctional and Detention Facilities. 2021.https://www.cdc.gov/coronavirus/2019-ncov/community/correction-detention/guidance-correctional-detention.html (accessed 1 Nov 2022).
39 WASH4Work. Hand hygiene protocol for the workplace. 2020.
40 WHO. Preparedness, prevention and control of COVID-19 in prisons and other places of detention: interim guidance. Copenhagen, Denmark: 2021.
41 CDC. When & How to Wash Your Hands. 2021.https://www.cdc.gov/handwashing/when-

For peer review only

how-handwashing.html (accessed 1 Nov 2021).
42 CDC. Hand Hygiene in School and Early Care and Education. 2021.
43 Global WASH Cluster. Global WASH Cluster – COVID 19 Response Guidance Note # 02 – Update 15 April 2020. 2020. https://reliefweb.int/report/world/global-wash-cluster-covid-19-response-guidance-note-02-update-15-april-2020
44 Global WASH Cluster. Health and Hygiene Promotion Guidance Document for COVID-19 – 29 March 2020. 2020.
45 Oxfam. Handwashing technical briefing note.
46 WHO. Guidelines on sanitation and health. Geneva, Switzerland: 2018. http://www.who.int/water_sanitation_health/publications/guidelines-on-sanitation-and-health/en/
47 Malteser International. WASH Guidelines for Field Practitioners: Hygiene. Cologne, Germany: 2017.
48 UNHCR. Technical WASH Guidance for COVID-19 Preparedness and Response. 2020.
49 UNICEF. Understanding Hygiene promotion in the context of Risk Communication & Community Engagement (RCCE) and Infection Control and Prevention (IPC) for the COVID-19 outbreak. New York, N.Y.: 2020.
50 UNICEF, GIZ. Scaling up Group Handwashing in Schools: Compendium of Group Handwashing Facilities Across the Globe. 2016.
51 WHO. Considerations in adjusting public health and social measures in the context of COVID-19. Geneva, Switzerland: 2020. https://www.who.int/publications-detail/risk-
52 UNICEF, SNV, GIZ. Handwashing with soap in schools: a technical options manual. 2020.
53 WHO, IFRC, UNICEF. Key Messages and Actions for Prevention and Control in Schools. 2020.
54 UNICEF, WHO, IFRC. Interim Guidance for COVID-19 Prevention and Control in Schools. New York, N.Y.: 2020. https://www.unicef.org/reports/key-messages-and-actions-coronavirus-disease-covid-19-prevention-and-control-schools
55 WHO. Public Health for Mass Gatherings: Key Considerations. Geneva, Switzerland: 2015. https://apps.who.int/iris/bitstream/handle/10665/162109/WHO_HSE_GCR_2015.5_eng.pdf;jsessionid=20CDEF66BA427C7DB54004047BD811E4?sequence=1
56 WHO. Safe Eid Al Adha Practices In The Context Of COVID-19 Interim Guidance. Geneva, Switzerland: 2021.
57 WHO. Safe Ramadan practices in the context of COVID-19. Geneva, Switzerland: 2021.
58 ICRC. Water, Sanitation, Hygiene and Habitat in Prisons. Geneva, Switzerland: 2013. www.icrc.org
59 ICRC. Health care in detention. Geneva, Switzerland: 2015. https://www.icrc.org/en/publication/4213-health-care-detention-practical-guide
60 UNICEF. COVID-19 Handwashing with Soap (HWWS) Facilities: Compendium of Indicative Layouts, Designs and Cost Estimates. New York, N.Y.: 2020.
61 Water Mission. Handwashing station program: requirements and recommendations. North Charleston, South Carolina, U.S.: 2020.
62 SNV. Practical options for handwashing stations: a guide for promoters and producers. The Hague, Netherlands: https://snv.org/assets/explore/download/202012-practical-_options-for-handwashing-stations.pdf
63 UNICEF. COVID-19 Emergency Preparedness and Response WASH and Infection Prevention and Control Measures in Schools. New York, New York: 2020.
64 WHO. Water, sanitation and hygiene standards for schools in low-cost settings. Geneva, Switzerland: 2009. doi:10.1080/00207233.2014.913878
65 WHO. Actions for consideration in the care and protection of vulnerable population groups from COVID-19. Geneva, Switzerland: 2021. http://iris.wpro.who.int/handle/10665.1/14549%0Ahttps://apps.who.int/iris/handle/10665/333043

For peer review only

66 WHO. Guidelines for drinking-water quality, 4th edition. 2017.
67 UNICEF. Handwashing Stations and Supplies for the COVID-19 response. New York, New York: 2020.
68 GOAL. WASH and Infection Prevention and Control (IPC) Measures in Households and Public spaces. 2020.
69 UNICEF. WASH and Infection Prevention and Control (IPC) Measures in Households and Public Spaces. New York, N.Y.: 2020.
70 Michie S, Richardson M, Johnston M, *et al.* The behavior change technique taxonomy (v1) of 93 hierarchically clustered techniques: building an international consensus for the reporting of behavior change interventions. *Ann Behav Med* 2013;**46**:81–95. doi:10.1007/s12160-013-9486-6
71 ILO. Safe return to work: Guide for employers on COVID-19 prevention. Geneva, Switzerland: 2020.
72 WHO. Guidance on COVID-19 for the care of older people and people living in long-term care facilities, other non-acute care facilities and home care. 2020.
73 UNICEF. A Manual on Hygiene Promotion. New York, N.Y.: 1999. http://www.unicef.org/wash/files/hman.pdf
74 WHO. WHO recommended strategies for the prevention and control of communicable diseases. Geneva, Switzerland: 2001.
75 Curtis VA, Danquah LO, Aunger R V. Planned, motivated and habitual hygiene behaviour: An eleven country review. *Health Educ Res* 2009;**24**:655–73. doi:10.1093/her/cyp002
76 SNV. Behaviour Change Communication Guidelines. Thimphu, Bhutan: 2016. https://snv.org/cms/sites/default/files/explore/download/snv_behaviour_change_communication_guidelines_-_april_2016.pdf
77 UNHCR. UNHCR Hygiene Promotion Guidelines. 2017. https://wash.unhcr.org/download/hygiene-promotion-guidelines-unhcr-2017/
78 WHO, ILO. Preventing and mitigating COVID-19 at work. Geneva, Switzerland: 2021. https://www.who.int/publications/i/item/WHO-2019-nCoV-workplace-actions-policy-brief-2021-1
79 WHO. Key Planning Recommendations for Mass Gatherings in the Context of COVID-19. Geneva, Switzerland: 2020.
80 Aiello AE, Larson EL, Levy SB. Consumer antibacterial soaps: Effective or just risky? *Clin Infect Dis* 2007;**45**. doi:10.1086/519255
81 Kumpel E, Billava N, Nayak N, *et al.* Water use behaviors and water access in intermittent and continuous water supply areas during the COVID-19 pandemic. *J Water Health* 2022;**20**:139–48. doi:10.2166/WH.2021.184
82 Todd E, Michaels B, Holah J, *et al.* Outbreaks where food workers have been implicated in the spread of foodborne disease. Part 10. Alcohol-based antiseptics for hand disinfection and a comparison of their effectiveness with soaps. *J Food Prot* 2010;**73**:2128–40.
83 Charbonneau D, Ponte J, Kochanowski B. A method of assessing the efficacy of hand sanitizers: use of real soil encountered in the food service industry. *J Food Prot* 2000;**63**:495–501.
84 Burton M, Cobb E, Donachie P, *et al.* The effect of handwashing with water or soap on bacterial contamination of hands. *Int J Environ Res Public Health* 2011;**8**:97–104.
85 Howard G, Bartram J, Brocklehurst C, *et al.* COVID-19: Urgent actions, critical reflections and future relevance of 'WaSH': Lessons for the current and future pandemics. *J Water Health* 2020;**18**:613–30. doi:10.2166/wh.2020.162
86 Huang C, Ma W, Stack S. The hygienic efficacy of different hand-drying methods: A review of the evidence. *Mayo Clin Proc* 2012;**87**:791–8. doi:10.1016/j.mayocp.2012.02.019
87 Gustafson D, Vetter E, Larson D, *et al.* Effects of 4 hand-drying methods for removing bacteria from washed hands: a randomized trial. *Mayo Clin Proc* 2000;**75**:705–8.

For peer review only

88 Verbyla ME, Pitol AK, Navab-Daneshmand T, *et al.* Safely Managed Hygiene: A Risk-Based Assessment of Handwashing Water Quality. *Environ Sci Technol* 2019;**53**:2852–61. doi:10.1021/acs.est.8b06156

89 Hoque B. Handwashing practices and challenges in Bangladesh. *Int J Environ Health Res* 2004;**13**:S81–7. doi:https://doi.org/10.1080/0960312031000102831

90 Luby S, Agboatwalla M, Painter J, *et al.* Effect of intensive handwashing promotion on childhood diarrhea in high-risk communities in Pakistan: A randomized controlled trial. *J Am Med Assoc* 2004;**291**:2547–54. doi:10.1001/jama.291.21.2547

91 Paludan-Müller AS, Boesen K, Klerings I, Jørgensen KJ MK. Hand cleaning with ash for reducing the spread of viral and bacterial infections: a rapid review. *Cochrane Database Syst Rev* Published Online First: 2020. doi:10.1002/14651858.CD013597.www.cochranelibrary.com

92 Amin N, Pickering AJ, Ram PK, *et al.* Microbiological evaluation of the efficacy of soapy water to clean hands: A randomized, non-inferiority field trial. *Am J Trop Med Hyg* 2014;**91**:415–23. doi:10.4269/ajtmh.13-0475

93 United Nations Children's Fund (UNICEF), World Health Organization (WHO). *State of the World's Hand Hygiene: A global call to action to make hand hygiene a priority in policy and practice*. New York, New York: : UNICEF 2021.

94 White S, Thorseth AH, Dreibelbis R, *et al.* The determinants of handwashing behaviour in domestic settings: An integrative systematic review. *Int J Hyg Environ Health* 2020;**227**:113512. doi:10.1016/J.IJHEH.2020.113512

95 Michie S, Stralen M van, West R. The behaviour change wheel: A new method for characterising and designing behaviour change interventions. *Implement Sci* 2011;**6**. doi:10.1001/archderm.1985.01660070119033

96 Aunger R, Curtis V. Behaviour Centred Design: towards an applied science of behaviour change. *Health Psychol Rev* 2016;**10**:425–46. doi:10.1080/17437199.2016.1219673

97 West R, Michie S. A brief introduction to the COM-B Model of behaviour and the PRIME Theory of motivation. 2020.https://www.qeios.com/read/WW04E6.2

98 Curtis V, Kanki B, Cousens S, *et al.* Dirt and diarrhoea formative research in hygiene promotion programmes. *Health Policy Plan* 1997;**12**:122–31.

For peer review only

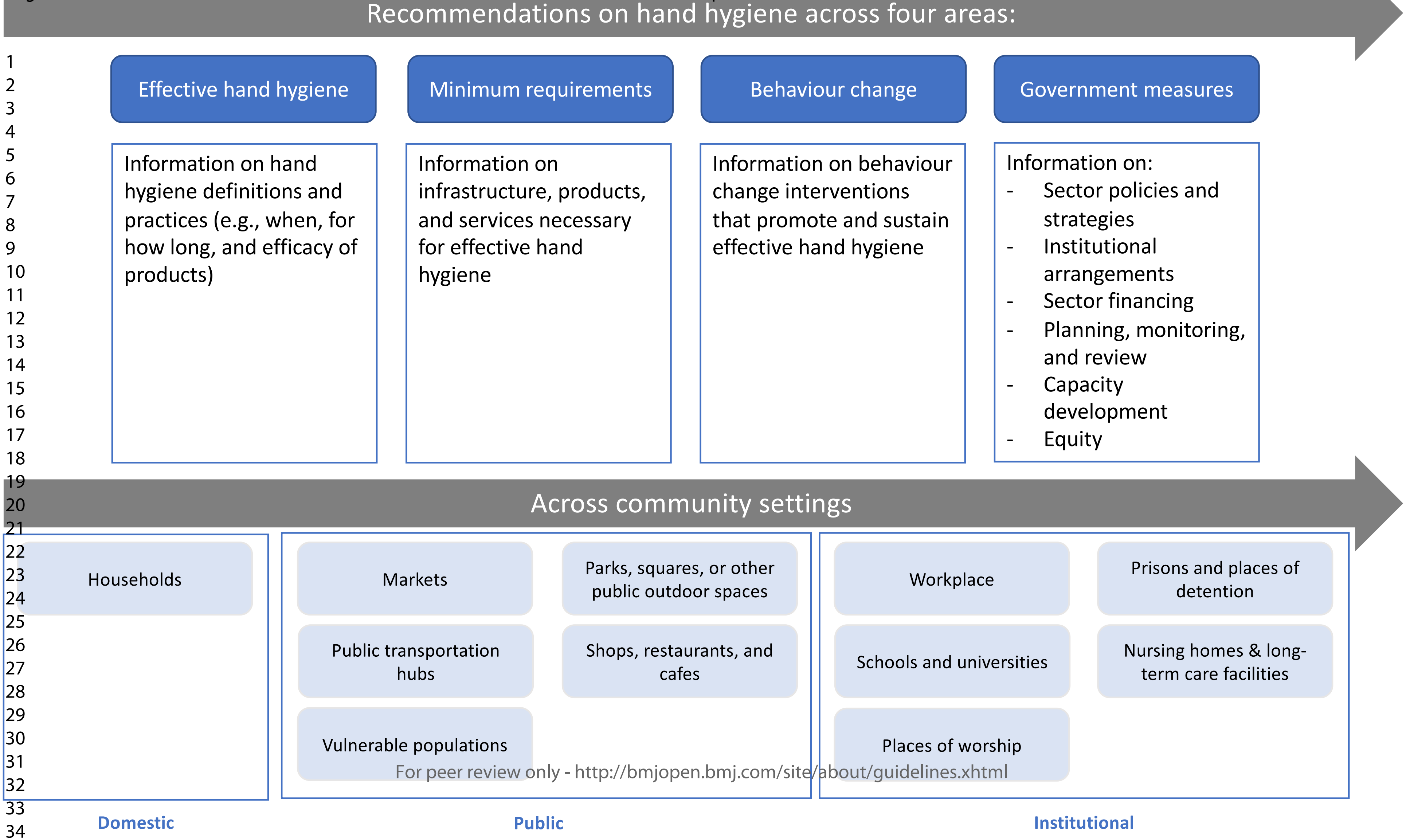

For peer review only - http://bmjopen.bmj.com/site/about/guidelines.xhtml

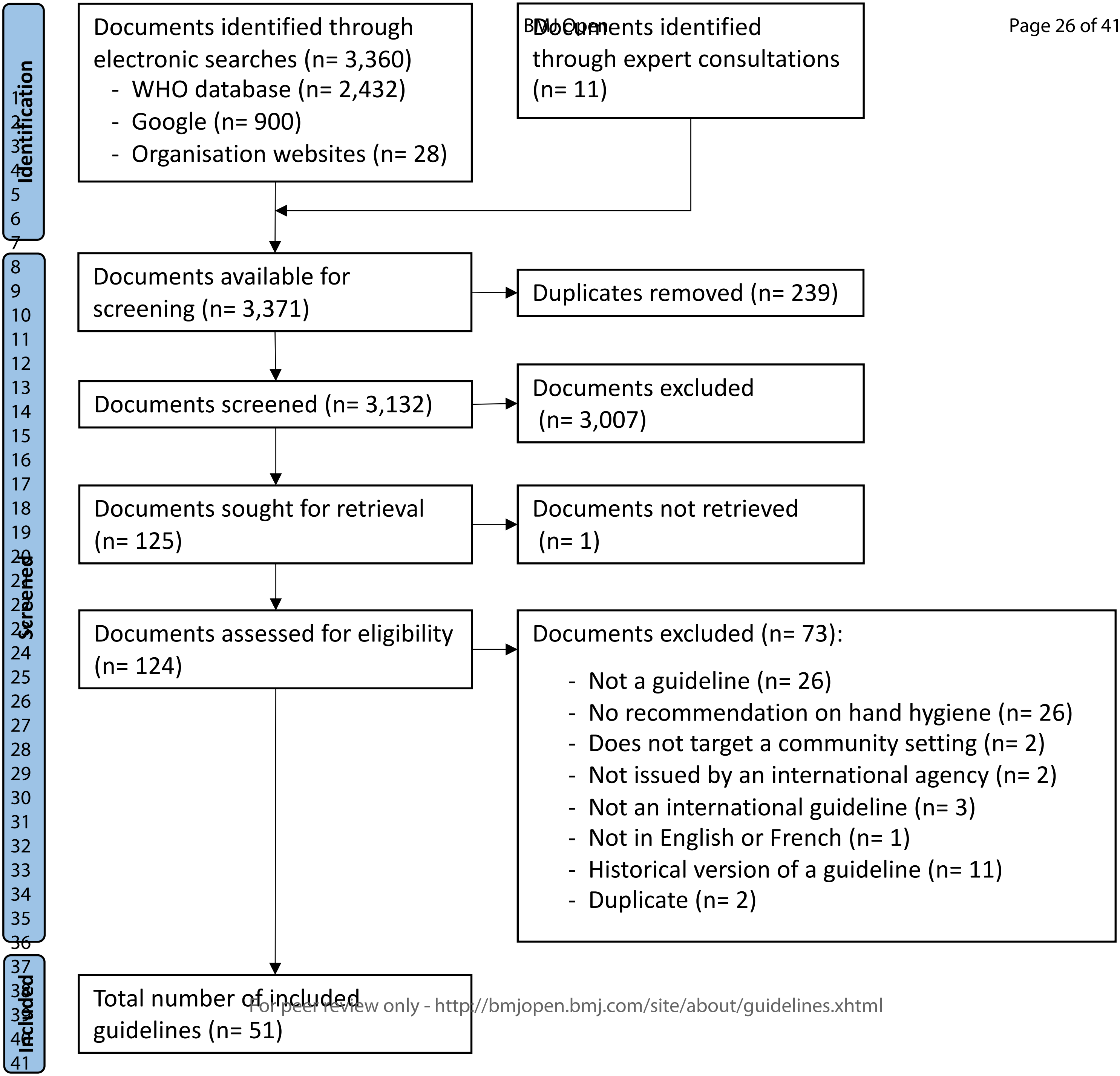

For peer review only - http://bmjopen.bmj.com/site/about/guidelines.xhtml

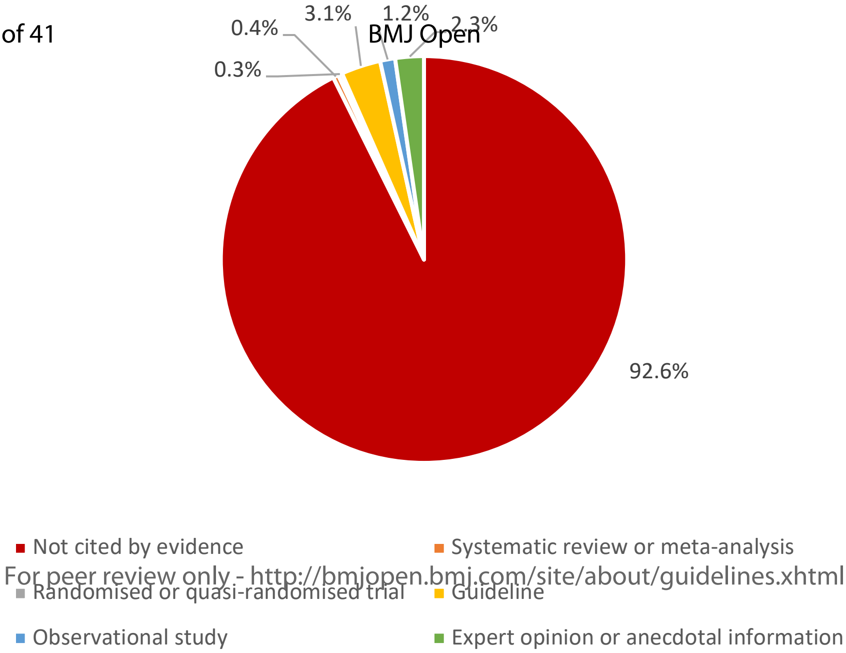

For peer review only - http://bmjopen.bmj.com/site/about/guidelines.xhtml

SUPPLEMENTAL MATERIALS

Table A1. The completed Preferred Reporting Items for Systematic reviews and Meta-Analyses extension for Scoping Reviews (PRISMA-ScR) checklist.[25]

SECTION	ITEM	PRISMA-ScR CHECKLIST ITEM	REPORTED ON PAGE #
**TITLE**			
Title	1	Identify the report as a scoping review.	2
**ABSTRACT**			
Structured summary	2	Provide a structured summary that includes (as applicable): background, objectives, eligibility criteria, sources of evidence, charting methods, results, and conclusions that relate to the review questions and objectives.	3
**INTRODUCTION**			
Rationale	3	Describe the rationale for the review in the context of what is already known. Explain why the review questions/objectives lend themselves to a scoping review approach.	5
Objectives	4	Provide an explicit statement of the questions and objectives being addressed with reference to their key elements (e.g., population or participants, concepts, and context) or other relevant key elements used to conceptualize the review questions and/or objectives.	5
**METHODS**			
Protocol and registration	5	Indicate whether a review protocol exists; state if and where it can be accessed (e.g., a Web address); and if available, provide registration information, including the registration number.	5
Eligibility criteria	6	Specify characteristics of the sources of evidence used as eligibility criteria (e.g., years considered, language, and publication status), and provide a rationale.	6
Information sources	7	Describe all information sources in the search (e.g., databases with dates of coverage and contact with authors to identify additional sources), as well as the date the most recent search was executed.	6
Search	8	Present the full electronic search strategy for at least 1 database, including any limits used, such that it could be repeated.	Supplemental Material – Table A3
Selection of sources of evidence	9	State the process for selecting sources of evidence (i.e., screening and eligibility) included in the scoping review.	6
Data charting process	10	Describe the methods of charting data from the included sources of evidence (e.g., calibrated forms or forms that have been tested by the team before their use, and whether data charting was done independently or in duplicate) and any processes for obtaining and confirming data from investigators.	7
Data items	11	List and define all variables for which data were sought and any assumptions and simplifications made.	7 and Supplemental Material – Table A5

For peer review only

SECTION	ITEM	PRISMA-ScR CHECKLIST ITEM	REPORTED ON PAGE #
Critical appraisal of individual sources of evidence	12	If done, provide a rationale for conducting a critical appraisal of included sources of evidence; describe the methods used and how this information was used in any data synthesis (if appropriate).	7
Synthesis of results	13	Describe the methods of handling and summarizing the data that were charted.	7
**RESULTS**			
Selection of sources of evidence	14	Give numbers of sources of evidence screened, assessed for eligibility, and included in the review, with reasons for exclusions at each stage, ideally using a flow diagram.	7 – 8
Characteristics of sources of evidence	15	For each source of evidence, present characteristics for which data were charted and provide the citations.	Supplemental material Table A7
Critical appraisal within sources of evidence	16	If done, present data on critical appraisal of included sources of evidence (see item 12).	15
Results of individual sources of evidence	17	For each included source of evidence, present the relevant data that were charted that relate to the review questions and objectives.	8 – 15
Synthesis of results	18	Summarize and/or present the charting results as they relate to the review questions and objectives.	8 – 15
**DISCUSSION**			
Summary of evidence	19	Summarize the main results (including an overview of concepts, themes, and types of evidence available), link to the review questions and objectives, and consider the relevance to key groups.	15 – 18
Limitations	20	Discuss the limitations of the scoping review process.	17 – 18
Conclusions	21	Provide a general interpretation of the results with respect to the review questions and objectives, as well as potential implications and/or next steps.	18
**FUNDING**			
Funding	22	Describe sources of funding for the included sources of evidence, as well as sources of funding for the scoping review. Describe the role of the funders of the scoping review.	20

For peer review only

Table A2. List of websites of international organisations known to work on hand hygiene searched.

Agency or resource hub	Website
Africa Centers for Disease Control and Prevention (CDC)	https://africacdc.org/
Global Handwashing Partnership	https://globalhandwashing.org/
Global WASH Cluster	www.washcluster.org
International Red Cross and Red Crescent (ICRC)	www.icrc.org
International Federation of the Red Cross (IFRC)	www.ifrc.org
International Labour Organization (ILO)	https://www.ilo.org
International Centre for Diarrhoeal Disease Research Bangladesh (ICDDR'B)	www.icddrb.org
Oxfam	www.oxfam.org.uk
Sanitation Learning Hub	https://sanitationlearninghub.org/
Save the Children	www.nrc.no
SNV Netherlands Development Organisation	https://snv.org/
Sustainable Sanitation Alliance	https://www.susana.org/en/
United Nations Human Settlement Programme (UN Habitat)	https://unhabitat.org/
United Nations High Commissioner for Refugees (UNHCR)	www.unhcr.org
United Nations Children's Fund (UNICEF)	www.unicef.org
US Centers for Disease Control and Prevention (CDC)	www.cdc.gov
WASH'Em	https://www.washem.info/
WaterAid	https://www.wateraid.org/us/
World Bank	https://www.worldbank.org/en/home
Water, Education and Development Centre (WEDC)	www.wedc.lboro.ac.uk
Water and Sanitation for the Urban Poor (WSUP)	https://www.wsup.com/
Deutsche Gesellschaft für Internationale Zusammenarbeit (GIZ)	https://www.giz.de
United States Agency for International Development (USAID)	https://www.usaid.gov/

Table A3. Search terms.

Concept	Search terms
Hand hygiene	Handwash* OR hygiene OR hand wash* OR with soap OR hand hygiene* OR clean hands OR WASH
Community settings	community OR household OR domestic OR public space OR institution* OR work* OR occupation* OR school OR prison OR market* OR relig* OR education* OR detention*
Guideline	guideline OR guide OR handbook OR manual OR toolkit

For peer review only - http://bmjopen.bmj.com/site/about/guidelines.xhtml

Table A4. Hand Hygiene for All Initiative core partners**.**

United Nations Children's Fund
International Labour Organization
World Bank
United Nations High Commission for Refugees
COVID-19 Hygiene Hub
Sanitation and Water for All
Global Handwashing Partnership
International Federation of Red Cross
WaterAid

Table A5. Data extraction template.

#	Hand hygiene parameter	Guideline title 1 (author, year)		Guideline title 2 (author, year)		Guideline title 3 (author, year)	
		Recommendation	Evidence level[1]	Recommendation	Evidence level	Recommendation	Evidence level
**General**							
1	Community setting(s)						
2	Other guideline specification (e.g., COVID-19)						
**Effective hand hygiene**							
3	Hand hygiene definition						
4	Effective/safe hand hygiene definition						
5	Terminology for defining when to practice hand hygiene						
6	Key moments						
7	Basic handwashing knowledge						
8	Basic hand hygiene knowledge						
**Minimum requirements for effective hand hygiene**							
**Handwashing with soap**							
10	Soap						
11	Soap quantity						
12	Handwashing services						
13	Handwashing with soap duration						

For peer review only

14	Water quality						
15	Water quantity						
16	Alternative water sources for handwashing						
17	Wastewater management						
18	Location of handwashing stations						
19	Spacing and number of users per handwashing station						
**Hand drying**							
20	Hand drying materials						
21	Disposal of hand drying materials						
22	Conditional recommendations						
**Handwashing with soap alternatives**							
23	Alcohol-based hand rub and alcohol content						
24	Hand hygiene duration						
25	Other materials for hand hygiene						
26	Conditional recommendations						
27	Alternatives not recommended						
28	Location of hand hygiene stations						
**Hand hygiene station design considerations**							
29	Design principles						
30	Technical considerations						
31	Accessibility						
32	Local procurement and production						
33	Supply chain						
34	Cost and affordability						
35	Durability						
36	COVID-19 adaptations						
37	Other adaptations						

For peer review only

38	Operation and maintenance – responsibility						
39	Operation and maintenance – actions						
**Behaviour change**							
40	Behaviour change techniques						
41	Content of behaviour change messaging						
42	Behaviour change delivery channel						
43	Person or group tasked with delivering behaviour change intervention						
44	Frequency of behaviour change interventions						
45	Formative research						
46	Behaviour change models or frameworks						
47	Behaviour change approaches						
48	Hand hygiene determinants						
**Government measures – at-scale interventions**							
49	Strengthening markets for hand hygiene products and services						
50	Hygiene promotion practices						
51	At-scale behaviour change interventions						
**Government measures – SWA framework**							
52	Sector policy and strategy						
53	Institutional arrangements						
54	Sector financing						
55	Planning, monitoring, and review						
56	Capacity development						
57	Equity						

For peer review only

[1] One of the five levels of evidence used for the review: 1) systematic review or meta-analysis, 2) randomised controlled trial or controlled trial without randomisation (e.g., quasi-experimental), 3) guideline (either developed from systematic reviews or not developed from systematic reviews), 4) observational studies (e.g., cohort, cross-sectional, case-control studies), and 5) expert opinion or anecdotal information (e.g., programme documentation).

Table A6. Excluded documents with reasons.

Author	Guideline title	Year	Reason for exclusion
ICRC	Water, sanitation, and hygiene, and habitat in prisons	2005	Historical version of guideline
World Bank	The Handwashing Handbook: a guide for developing a hygiene promotion program to increase handwashing with soap	2005	Historical version of guideline
IRC	Strengthening Water, Sanitation and Hygiene in schools: a WASH guidance manual with a focus on South Asia	2010	Not a guideline
ICRC	Water, sanitation, and hygiene, and habitat in prisons: supplementary guidance	2012	No recommendation on hand hygiene
GIZ	Field guide: hardware for handwashing in schools	2013	Not a guideline
WEDC	Guidelines for handwashing with soap	2013	Not issued by an international agency
NRC	Water, sanitation and hygiene manual: WASH training for hygiene promotion staff	2015	Not a guideline
ILO	WASH@Work: self-training handbook	2016	Historical version of guideline
WSUP	School hygiene manual	2016	Not for international audience
Rotary	A guide to WASH in schools	2017	Not a guideline
Global Handwashing Partnership	Clean hands for all: a toolkit for hygiene advocacy	2018	Not a guideline
COOPI	WASH handbook for teachers and facilitators	2019	Not for international audience
WASH'Em	How to design handwashing facilities that change behaviour	2019	Does not target a community setting
Africa CDC	Hand washing facility options for resource limited settings	2020	Not a guideline
British Psychological Society	Behavioural science and disease prevention psychological guidance: encouraging hand hygiene in the community	2020	Not a guideline

Coultas et al.	Handwashing compendium for low-resource settings: a living document, 3rd edition	2020	Not a guideline
Global Handwashing Partnership	The Handwashing Handbook	2020	Not a guideline
Smart Centre Group	Smart hygiene solutions: affordable options for household level	2020	Not a guideline
UN Habitat	UN-Habitat guidance on COVID-19 and public spaces	2020	No recommendation on hand hygiene
UNHCR	COVID-19 refugees return to schooling guidelines	2020	No recommendation on hand hygiene
UNICEF et al.	Panduan Cuci Tangan Pakai Sabun	2020	Not in English or French
WaterAid	COVID-19 guidance: prioritising hygiene for workforce health and business resilience	2020	No recommendation on hand hygiene
WaterAid Nepal	Technical brief on contactless handwashing stations	2020	Not a guideline
Bolton, L.	WASH in schools for student return during the COVID-19 pandemic	2021	Not issued by an international agency
ILO	WASH@Work: self-training handbook (revised)	2021	Not a guideline
Government of Vietnam	A guide to integrated hand washing with soap communication	n/d	Not for international audience
WaterAid	Technical manual on community water supply, hygiene and sanitation facilities	n/d	Not a guideline
WaterAid Bangladesh	Handwashing stations: an easy-to-use technological and context-based handwashing stations manual	n/d	Not a guideline

For peer review only

Table A7. List of included guidelines.

Author	Guideline	Year	Community setting	COVID-19 response
UNICEF	A manual on hygiene promotion	1999	Public	No
WHO	WHO recommended strategies for the prevention and control of communicable diseases	2001	Domestic & public	No
WHO	Health villages: a guide for communities and community health workers	2002	Public	No
WHO	Water, sanitation, and hygiene standards for schools in low-cost settings	2009	Institutional	No
ICRC	Water, sanitation, hygiene, and habitat in prisons	2013	Institutional	No
ICRC	Health care in detention	2015	Institutional	No
WHO	Public health for mass gatherings: key considerations	2015	Public	No
SNV	Behaviour change communication guidelines	2016	Public	No
UNICEF, GIZ	Scaling up group handwashing in schools: compendium of group washing facilities across the globe	2016	Schools	No
Malteser	WASH guidelines for field practitioners	2017	Multiple settings	No
Oxfam	Handwashing technical briefing note	2017	Public	No
UNHCR	UNHCR hygiene promotion guidelines	2017	Public	No
WHO	Guidelines on sanitation and health	2018	Domestic & public	No
WHO	Recommendations to Member States to improve hand hygiene practices to help prevent COVID-19	2020	Public	Yes
UNICEF	Handwashing stations and supplies for the COVID-19 response	2020	Domestic & public	Yes
ILO	Hand hygiene at the workplace: an essential occupational safety and health prevention and control measure against COVID-19	2020	Workplace	Yes
GOAL	WASH and IPC measures in households and public spaces	2020	Domestic & public	No
WHO	Considerations for community hand hygiene practices in low-resource situations	2020	Public	No
WHO	Overview of public health and social measures in the context of COVID-19	2020	Public	Yes
GWC	COVID-19 response guidance note - 15 April 2020	2020	Domestic & public	Yes
GWC	Health and hygiene promotion guidance document for COVID-19	2020	Public	Yes
ILO	Safe return to work: guide for employers on COVID-19 prevention	2020	Institutional	Yes
SNV	Practical options for hand-washing stations: a guide for promoters and producers	2020	Multiple settings	No
UNHCR	Technical WASH guidance for COVID-19 preparedness and response	2020	Public	Yes
UNICEF	COVID-19 emergency preparedness and response: WASH and infection prevention and control measures in schools	2020	Schools	Yes
UNICEF	COVID-10 handwashing with soap (HWWS) facilities: compendium of indicative layouts, designs, and cost estimates	2020	Public	Yes

For peer review only

UNICEF et al.	Handwashing with soap in schools: a technical options manual	2020	Schools	No
UNICEF, WHO, IFRC	Key messages and actions for COVID-19 prevention and control in schools	2020	Schools	Yes
UNICEF	Understanding Hygiene promotion in the context of Risk Communication & Community Engagement (RCCE) and Infection Control and Prevention (IPC) for the COVID-19 outbreak	2020	Domestic & public	Yes
UNICEF	WASH and Infection Prevention and Control (IPC) measures in households and public spaces	2020	Domestic & public	Yes
UNICEF, WHO, IFRC	Interim guidance for COVID-19 prevention and control in schools	2020	Institutional	Yes
WASH4WORK	Hand hygiene protocol for the workplace	2020	Institutional	Yes
Water Mission	Handwashing station program: requirements and recommendations	2020	Public	Yes
WaterAid	Technical guide for handwashing facilities in public spaces and buildings	2020	Public	Yes
WHO	Actions for consideration in the case and protection of vulnerable populations from COVID-19	2020	Vulnerable populations	Yes
WHO	Considerations for public health and social measures in the workplace in the context of COVID-19: Annex to Considerations in adjusting public health and social measures in the context of COVID-19	2020	Workplace	Yes
WHO	Considerations for school-related public health measures in the context of COVID-19: Annex to Considerations in adjusting public health and social measures in the context of COVID-19	2020	Institutional	Yes
WHO	Guidance on COVID-19 for the care of older people and people living in long-term care facilities, other non- acute care facilities and home care	2020	Institutional	Yes
WHO	Interim recommendations on obligatory hand hygiene against transmission of COVID-19	2020	Public spaces	Yes
WHO	Practical considerations and recommendations for religious leaders and faith-based communities in the context of COVID-19	2020	Public spaces	Yes
WHO	Water, sanitation, hygiene, and waste management for SARS-CoV-2, the virus that causes COVID-19: interim guidance	2020	Domestic & public	Yes
CDC	When and how to wash your hands	2021	Public	No
CDC	Hand hygiene at school	2021	Institutional	No
CDC	Hand hygiene at work	2021	Institutional	No
CDC	Interim guidance on management of coronavirus disease 2019 (COVID-19) in correctional and detention facilities	2021	Institutional	Yes
CDC	Interim guidance on people experiencing unsheltered homelessness	2021	Vulnerable populations	Yes
WHO	Key planning recommendations for mass gatherings in the context of COVID-19: interim guidance	2021	Public	Yes

WHO	Preparedness, prevention, and control of COVID-19 in prisons and other places of detention: interim guidance	2021	Institutional	Yes
WHO, ILO	Preventing and mitigating COVID-19 at work	2021	Institutional	Yes
WHO	Safe Eid al Adha practices in the context of COVID-19: interim guidance	2021	Public	Yes
WHO	Safe Ramadan practices in the context of COVID-19: interim guidance	2021	Public	Yes

For peer review only

Table A8. Number of recommendations per parameter across all community settings.

Parameters	Number of recommendations	Number of guidelines with recommendations
**Effective hand hygiene**		
Guidelines that define hand hygiene	3	3
Guidelines that define safe hand hygiene	2	2
Terminology for defining when to practice safe hand hygiene	38	38
Critical control points	145	38
Basic handwashing knowledge	8	8
Basic hand hygiene knowledge	0	0
**Handwashing with soap & hand drying**		
Handwashing infrastructure	33	24
Handwashing materials	68	46
Amount of soap	7	6
Handwashing services	37	30
Handwashing with soap duration	17	17
Water quality	19	16
Water quantity	9	10
Alternative water sources for handwashing	15	16
Wastewater management	23	13
Location of handwashing stations	59	30
Spacing and number of users per handwashing station	9	8
Hand drying materials	36	19
Disposal of hand drying materials	9	9
Conditional recommendations	7	7
**Handwashing with soap alternatives**		
Alcohol-based hand rub	32	32
Hand rubbing duration	3	3
Other materials for hand hygiene	39	17
Conditional recommendations	29	22
Alternatives not recommended	11	7
Location of hand hygiene stations	21	12
**Hand hygiene station design considerations**		
Design principles	10	6
Technical considerations	5	4
Accessibility	19	14
Local procurement and production	11	9
Supply chain	3	3
Cost and affordability	7	7
Durability	8	6
COVID-19 adaptations	26	16
Other adaptations	17	10
Operation and maintenance – responsibility	8	6
Operation and maintenance – actions	9	8
**Changing behaviour**		
Behaviour change techniques	29	14
Content of behaviour change messaging	27	25
Behaviour change delivery channels	42	25
Person or group tasked with delivery behaviour change intervention	13	10
Frequency of behaviour change interventions	2	2
Formative research	5	4

For peer review only

Behaviour change models or frameworks	15	3
Behaviour change approaches	9	2
Hand hygiene determinants	11	3
**Government interventions**		
Strengthening markets for hand hygiene products and services	2	2
Hygiene promotion practices	0	0
At-scale behaviour change interventions	0	0
Sector policy and strategy	0	0
Institutional arrangements	5	5
Sector financing	0	0
Planning, monitoring, and review	2	2
Capacity development	1	1
Equity	1	1

For peer review only

Table 1. The completed Preferred Reporting Items for Systematic reviews and Meta-Analyses extension for Scoping Reviews (PRISMA-ScR) checklist.

SECTION	ITEM	PRISMA-ScR CHECKLIST ITEM	REPORTED ON PAGE #
**TITLE**			
Title	1	Identify the report as a scoping review.	2
**ABSTRACT**			
Structured summary	2	Provide a structured summary that includes (as applicable): background, objectives, eligibility criteria, sources of evidence, charting methods, results, and conclusions that relate to the review questions and objectives.	3
**INTRODUCTION**			
Rationale	3	Describe the rationale for the review in the context of what is already known. Explain why the review questions/objectives lend themselves to a scoping review approach.	5
Objectives	4	Provide an explicit statement of the questions and objectives being addressed with reference to their key elements (e.g., population or participants, concepts, and context) or other relevant key elements used to conceptualize the review questions and/or objectives.	5
**METHODS**			
Protocol and registration	5	Indicate whether a review protocol exists; state if and where it can be accessed (e.g., a Web address); and if available, provide registration information, including the registration number.	5
Eligibility criteria	6	Specify characteristics of the sources of evidence used as eligibility criteria (e.g., years considered, language, and publication status), and provide a rationale.	6
Information sources	7	Describe all information sources in the search (e.g., databases with dates of coverage and contact with authors to identify additional sources), as well as the date the most recent search was executed.	6
Search	8	Present the full electronic search strategy for at least 1 database, including any limits used, such that it could be repeated.	Supplemental Material – Table A3
Selection of sources of evidence	9	State the process for selecting sources of evidence (i.e., screening and eligibility) included in the scoping review.	6
Data charting process	10	Describe the methods of charting data from the included sources of evidence (e.g., calibrated forms or forms that have been tested by the team before their use, and whether data charting was done independently or in duplicate) and any processes for obtaining and confirming data from investigators.	7
Data items	11	List and define all variables for which data were sought and any assumptions and simplifications made.	7 and Supplemental Material – Table A5
Critical appraisal of individual sources of evidence	12	If done, provide a rationale for conducting a critical appraisal of included sources of evidence; describe the methods used and how this information was used in any data synthesis (if appropriate).	7

For peer review only

SECTION	ITEM	PRISMA-ScR CHECKLIST ITEM	REPORTED ON PAGE #
Synthesis of results	13	Describe the methods of handling and summarizing the data that were charted.	7
**RESULTS**			
Selection of sources of evidence	14	Give numbers of sources of evidence screened, assessed for eligibility, and included in the review, with reasons for exclusions at each stage, ideally using a flow diagram.	7 – 8
Characteristics of sources of evidence	15	For each source of evidence, present characteristics for which data were charted and provide the citations.	Supplemental material Table A7
Critical appraisal within sources of evidence	16	If done, present data on critical appraisal of included sources of evidence (see item 12).	15
Results of individual sources of evidence	17	For each included source of evidence, present the relevant data that were charted that relate to the review questions and objectives.	8 – 15
Synthesis of results	18	Summarize and/or present the charting results as they relate to the review questions and objectives.	8 – 15
**DISCUSSION**			
Summary of evidence	19	Summarize the main results (including an overview of concepts, themes, and types of evidence available), link to the review questions and objectives, and consider the relevance to key groups.	15 – 18
Limitations	20	Discuss the limitations of the scoping review process.	17 – 18
Conclusions	21	Provide a general interpretation of the results with respect to the review questions and objectives, as well as potential implications and/or next steps.	18
**FUNDING**			
Funding	22	Describe sources of funding for the included sources of evidence, as well as sources of funding for the scoping review. Describe the role of the funders of the scoping review.	20

For peer review only
